# 6-hour Training in click-based echolocation changes practice in visual impairment professionals

**DOI:** 10.3389/fresc.2023.1098624

**Published:** 2023-05-22

**Authors:** Lore Thaler, Giammarco Di Gregorio, Denise Foresteire

**Affiliations:** Department of Psychology, Durham University Durham, United Kingdom

**Keywords:** content analysis, quantitative, qualitative, blindness, intervention

## Abstract

Click-based echolocation can support mobility and orientation in people with vision impairments (VI) when used alongside other mobility methods. Only a small number of people with VI use click-based echolocation. Previous research about echolocation addresses the skill of echolocation *per se* to understand how echolocation works, and its brain basis. Our report is the first to address the question of professional practice for people with VI, i.e., a very different focus. VI professionals are well placed to affect how a person with VI might learn about, experience or use click-based echolocation. Thus, we here investigated if training in click-based echolocation for VI professionals might lead to a change in their professional practice. The training was delivered via 6-h workshops throughout the UK. It was free to attend, and people signed up via a publicly available website. We received follow-up feedback in the form of yes/no answers and free text comments. Yes/no answers showed that 98% of participants had changed their professional practice as a consequence of the training. Free text responses were analysed using content analysis, and we found that 32%, 11.7% and 46.6% of responses indicated a change in information processing, verbal influencing or instruction and practice, respectively. This attests to the potential of VI professionals to act as multipliers of training in click-based echolocation with the potential to improve the lives of people with VI. The training we evaluated here could feasibly be integrated into VI Rehabilitation or VI Habilitation training as implemented at higher education institutions (HEIs) or continuing professional development (CPD).

## Introduction

Echolocation is the ability to obtain spatial information from sound echoes. Once a sound is generated, it travels through the air until it hits a surface and reflects back. The sound reflection, or echo, carries information about the nature and arrangement of the surface it bounced back from. Echolocation is probably best known from bats, but people can echolocate as well (1–3). Echolocation can be based on echoes from ambient sound, and it can be based on echoes from self emitted sounds like cane taps, footsteps, whistling, speech or mouth-clicks. Click-based echolocation refers to the use of active echolocation using mouth-clicks.

Research has shown that click-based echolocation provides spatial sensing advantages to people in low vision conditions e.g., people with vision impairments (VI) or people who are normally sighted using a blind-fold. It can be used to perceive a silent object's position in space as well as its shape, material, and whether it is in motion, i.e., properties that would not be available without vision (1–3). Click-based echolocation can also support successful avoidance of obstacles and adaptive walking (4, 5). Research has also shown that click-based echolocation can be learned by people who are normally sighted as well as by people who are blind (e.g., 6, 7). Use of and training in click-based echolocation, alongside other mobility methods such as long cane or guide dog for example, is associated with increased mobility, independence and wellbeing in people with VI (6, 8). In summary, click-based echolocation can be understood as a tool that supports spatial sensing, and that can support mobility and orientation in people with VI when used alongside other mobility methods.

Despite its relative rarity, click-based echolocation is gaining traction as a tool to improve independent mobility, including in low-income settings (6, 9). Currently, training in click-based echolocation is limited to self-training by trial and error and/or training with another echolocator. Thus, there is room for increasing access to and usage of this skill in people with VI.

People with VI in the UK receive sensory support and training in orientation and mobility by visual impairment professionals, e.g., rehabilitation or habilitation workers. Thus, VI professionals are well placed to affect how a person with VI might learn about, experience or use click-based echolocation. Thus, we here investigated if a 6-h training in click-based echolocation for VI professionals might lead to a change in their professional practice with respect to click-based echolocation.

Notably, all previous research about echolocation addresses the skill of echolocation *per se* and how it is learned in order to understand how echolocation works, and its brain basis. In contrast, here we address the question how training in echolocation changes the way that VI professionals address/use echolocation with their clients. Thus, our report has a different focus compared to previous work.

The training was delivered via 6-h workshops throughout the UK. It was free to attend, and people signed up via a publicly available website. From 166 people who had opted in to be contacted via e-mail for follow-up feedback we received 106 responses in the form of yes/no answers and free text comments. Using numerical as well as content analysis we found that 98% of respondents had changed their professional practice as a consequence of the training, with 32%, 11.7% and 46.6% of these indicating changes in information processing, changes in verbal influencing or changes in instruction and training, respectively. This attests to the potential of VI professionals to act as multipliers of training in click-based echolocation with the potential to improve the lives of people with VI.

The training we evaluated here, i.e., 6-h workshop, split into lecture, Q&A and practical exercises, could feasibly be integrated into VI training as implemented at higher education institutions (HEIs) or in continuing professional development (CPD). We advise that future studies should investigate the effects that changes in professional practice have on clients, i.e., people with VI.

## Data access statement

All data are available in the article and Supplementary Material. Further inquiries can be directed to the corresponding author/s.

## Method

### Training workshops

The training was delivered by the first author (LT), in the time period between 1/2018 and 12/2018 in the form of 18 echolocation workshops throughout the UK. Workshop dates and locations were advertised on a public website at Durham University. Attendees signed up via e-mail. Workshops were free to attend. Each workshop lasted 6-h and consisted of a lecture about click-based echolocation, followed by a Q&A session, followed by practical echolocation exercises. Attendees received copies of all materials used during the workshop (ppt slides). All materials are available as Supplementary Materials S1.

### Participants

**Figure 1:**
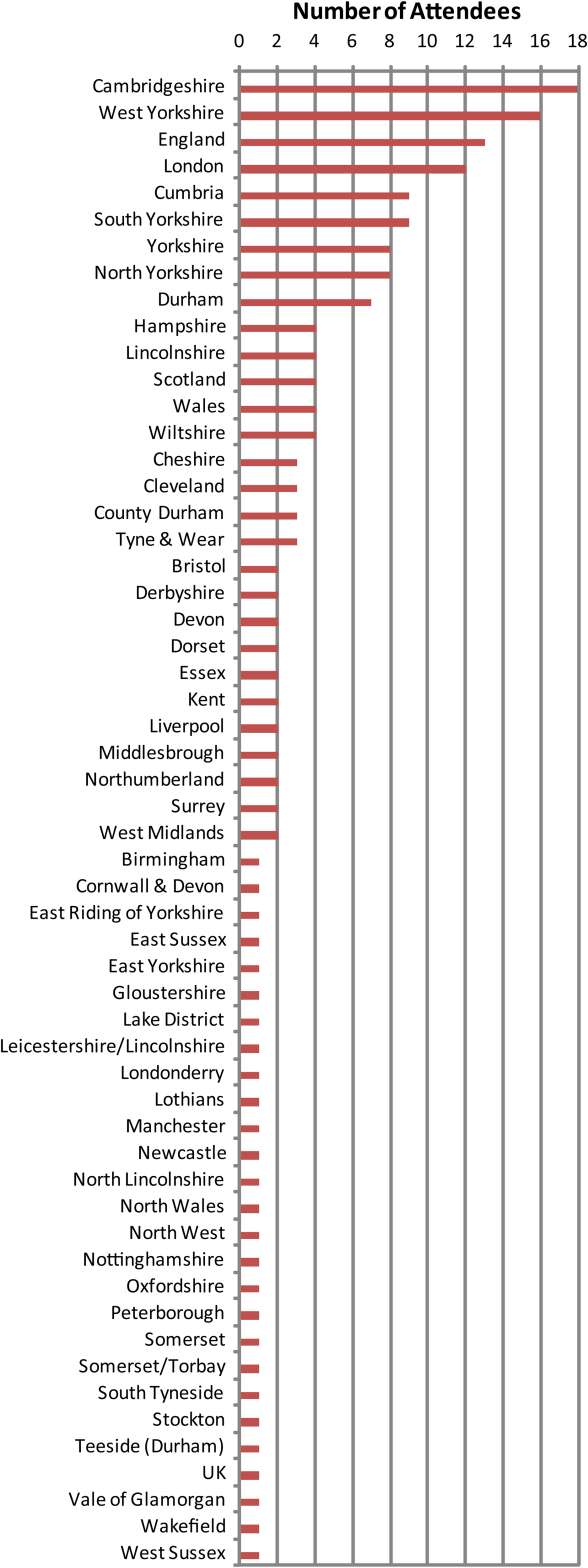
Geographical distribution of workshop attendees (181 out of 201 reported via opt-in questionnaire) sorted by number of attendees (larger groups first) and labelled by region that attendees reported coming from. Participants came from all over the UK, incl. Wales and Scotland, and England North to South, West through East.

**Figure 2:**
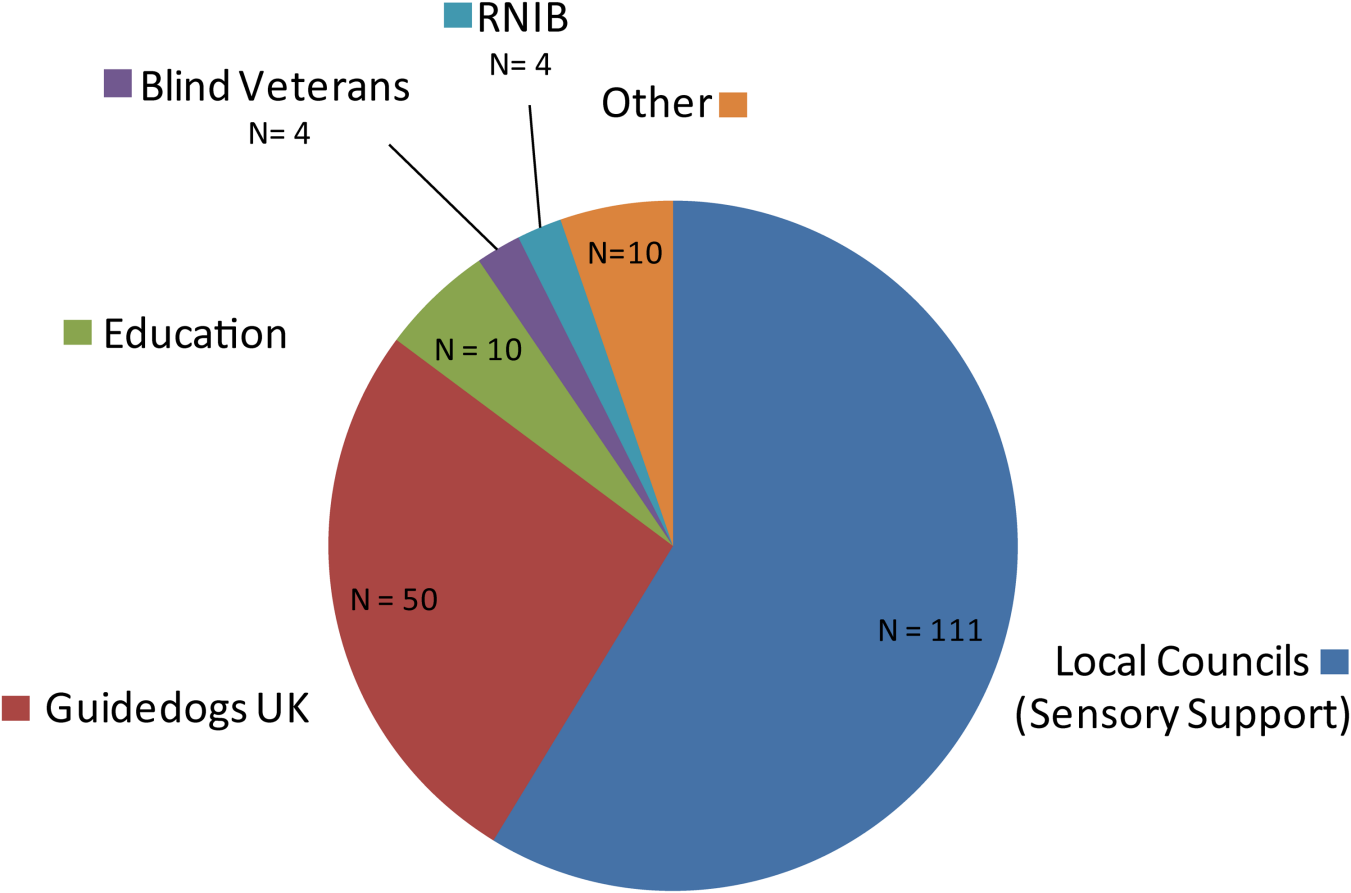
Organizational distribution of workshop attendees (189 out of 201 reported via opt in questionnaire). RNIB, royal national institute of blind people. “Education” comprises schools and universities. Majority of attendees were from Local Councils (i.e. sensory support teams) and Guidedogs UK.

Overall, 201 VI professionals attended the workshops. Using an opt-in questionnaire, 181 of the attendees reported to come from throughout the UK (Figure 1), and 189 of the attendees reported to come from a variety of organizations (Figure 2).

### Data collection

We collected participant feedback via e-mail four months after any workshop. 166 out of 201 (i.e., 82.6%) participants had given us permission to contact them for follow-up feedback via an opt-in list distributed at workshops. Out of those 166 we successfully contacted 154 (i.e., 92.8%) for follow-up (for the remaining 12 our message bounced e.g., because e-mail addresses had become invalid or we could not correctly decipher e-mail addresses from the opt-in list). Out of those 154 we received feedback from 106 (i.e., 68.8%). Out of those 106, one response was identified as a duplicate, and therefore one of these responses was removed from analysis. Thus, we had a total of 105 responses for analysis.

The e-mail contained two questions. The first question was a yes/no question. It read “Has what you learnt during the training affected your professional practice (e.g., better understanding, better awareness, making recommendations, instruction, etc.)? YES/NO”. The second question was a free text question. It read “If yes, in your own words how has it affected your professional practice?”.

### Data coding

Answers to yes/no Question 1 were counted.

Answers to free text Question 2 were analysed using content analysis (10). Specifically, a numerical code (0, 1, 2, 3) was assigned to each free text answer, based on the content of the text. Subsequently, we quantified how many responses had been coded as 0, 1, 2 or 3. Codes were assigned to responses using the coding scheme shown in Table 1. A code of “1” was applied to responses that indicated a change in information processing without any explicit element of verbal influencing or training. A code of “2” was applied to responses that indicated a change in verbal influencing, without any explicit element of training. A code of “3” was applied to responses that indicated a change in instruction and training. A code of “0” was applied if a response did not provide evidence for any of the other codes. For example, if comments were phrased hypothetically or relating to activities planned for the future, or if no free text comment had been provided, this would have been coded as “0”. Using this coding scheme, three coders independently coded participants' answers in two separate sessions seven days apart. The order of participant answers was randomized for each coding session. One of the coders was the first author (LT). The other two coders had not been involved in training or data collection.

**Table 1:** Coding scheme applied to free text Comments in response to question 2.

Coding scheme
Each code must refer to things that are described as happening/has happened (i.e., not hypothetical, not a desire or an intention). Anything that is being expressed as hypothetical cannot be coded as 1–3.
Code 0:
No response or any response that cannot be coded as 1–3
Code 1:
This code should be applied to responses indicating that participants have changed the way they process information with respect to echolocation e.g. when being with clients, client carers/friends, or colleagues, or when going over materials related to their professional practice/work. This can manifest in changes in e.g. perception, awareness, echolocation skill, knowledge, confidence, interpretation, assessment. Responses do not contain an explicit element of influencing or training others.
Code 2:
This code should be applied to responses indicating that participants have changed the way they influence others verbally with respect to echolocation (e.g. clients, client carers/friends, colleagues), e.g. during interactions, conversations, assessments. This can manifest itself in changes in e.g. advice, recommendation, discussion, questioning, referral (to other people or sources of information) regarding the use of echolocation/sound. Responses do not contain an explicit element of training others (e.g. clients, client carers/friends, colleagues).
NOTE: If someone states “…I have become more confident advising/discussing…” this would be coded as 2, rather than 1 because this refers to a change in the way they influence.
Code 3:
This code should be applied to responses indicating that participants have changed the way they provide instruction and training to others with respect to echolocation (e.g. clients, client carers/friends, colleagues). This can manifest itself in changes in e.g. lesson planning, instruction, communication behaviour during instruction, mobility, orientation, examples on how to use sound (e.g., now listen to that, what does it tell you?).
NOTE: If someone states “…I have become more confident training…” this would be coded as 3, rather than 1 because this refers to a change in the way they provide training.

Consistency of coding was assessed using Cohen's Kappa (11, 12). Intra-coder reliability, i.e., consistency for each coder across the two separate coding sessions, was high (kappa for each of the three coders were .93, .94 and .92). Inter-coder reliability, i.e., consistency across coders, was high as well (average: .82, median: .81, min: .77, max: .88, SD: .03). Thus, overall, there was excellent consistency. In cases where all three coders had assigned the same code (*n* = 83; 80.6%), that code was assigned to a response. In cases where coders disagreed the code chosen was either the most frequently assigned code (*n* = 15; 14.6%), or (in case of ties), the lowest code that had been assigned by any coder (*n* = 5; 4.9%).

All answers and codes that had been assigned by each coder for each session, as well as the final assigned code are provided in the Supplementary Materials S2.

## Results

**Figure 3:**
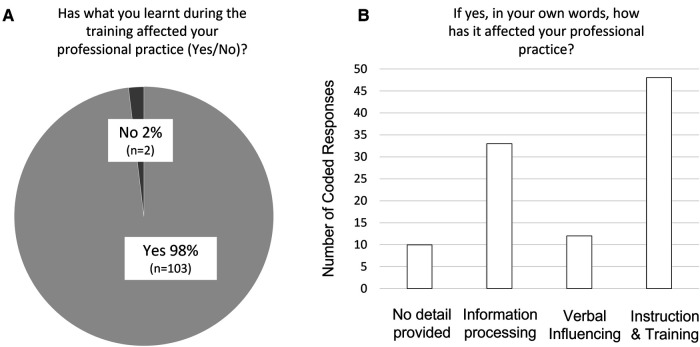
Visual summary of responses to question 1 (**A**) and coded responses to question 2 (**B**).

In response to Question 1, 103 out of 105 respondents (i.e., 98.1%) had answered with yes, reporting that what they learnt during the workshop has affected their professional practice (see Figure 3A).

Out of those 103, in response to Question 2, 10 responses (i.e., 9.7%) were assigned a code of “0”, meaning that even though the respondent had indicated a change in their professional practice in answer to question 1, their free text comment did not allow a coding into any of the change categories 1–3. A further 33 responses (i.e., 32%) were coded as 1, i.e., 32% of responses indicated that the respondent had changed the way they process information with respect to echolocation e.g., when being with clients, client carers/friends, or colleagues, or when going over materials related to their professional practice/work, without any change in the way they may verbally influence or train others. Examples of responses coded as 1 are below. All responses are available in Supplementary Material S2.

*“It has given me a better understanding of echolocation which has helped me with one of my learners. I now understand how she uses this to navigate her surroundings. The day was very informative and I have come away with lots of useful information. The practical activities really gave me a clear understanding of how to use echolocation and the difficulties with this. Thoroughly enjoyed the day.”*

“*I now understand about echolocation and how it could utilised by the students I work with alongside other mobility techniques and with a long cane. I am now open to the use of echolocation to aid mobility.”*

*“The training has provided an insight into the world of my 1:1. It has helped me understand what his world might be like using echolocation. Having the course provider teach me was a fantastic experience and skill to begin understand and learn. I had a fantastic training session.”*

*“A greater awareness of Echo-location, what it is and how it can be used and developed by CYPVI (note by author: CYPVI—Children and Young People with Vision Impairment). A general understanding of how to develop a CYPVI's harnessing of this skill.”*

Another 12 responses were coded as 2, i.e., 11.7% of responses indicated that the respondent had changed the way they verbally influence others with respect to echolocation (e.g., clients, client carers/friends, colleagues), e.g., during interactions, conversations, assessments, without indication for any change in the way they may train others (e.g., clients, client carers/friends, colleagues). Examples of responses coded as 2 are below. All responses are available in Supplementary Material S2.

“*I feel confident in encouraging children I work with to use/understand echolocation more than I did before.”*

“*I can now make an effective click. I had to work hard to achieve it so feel I can empathise with those that don't find it easy to produce a click. I feel more confident in explaining the pro's of echolocating and it's use. I would be confident to encourage a child to learn to use it and promote it with others around them. I thought the training was well delivered and interesting. It would be nice to offer more advanced techniques and further training to solidify learning.”*

*“Thank you so much for the training, it has been beneficial for me as a newly qualified professional as a Rehabilitation Worker for Visually impaired people (ROVI) absolutely. It has improved my knowledge and I have been able to impart that knowledge with staff and visually impaired people for them to consider Echolocation as an option. Since the training, I have discussed with staff how we could incorporate the discussion about Echolocation into our working practice.”*

*“Although my role does not involve working directly with our clients I have spoken to other organizations regarding Echolocation. I am exploring how we may work with yourselves and another organization to promote Echolocation to our clients.”*

Finally, 48 responses were coded as 3, i.e., 46.6% of responses indicated that the respondent had changed the way they provide instruction and training to others with respect to echolocation (e.g., clients, client carers/friends, colleagues). Examples of responses coded as 3 are below. All responses are available in Supplementary Material S2.

“*The training has given me the confidence to work with and develop students’ confidence with echolocation. It has enabled me to lay building blocks with younger children that I can build upon as they develop and mature. The training has also provided me with training materials which are invaluable, I now have a clear structure to work through when supporting students. I now factor the training into lessons and slowly introduce echolocation skills when working with young students. I can talk more confidently about echolocation with older students to get them thinking about how it can benefit them and compliment existing mobility skills. Training of this kind is very very rare in this line of work and to work with such inspirational people like X has been a privilege. They are passionate about their work and this is obvious during training sessions. Having this point of contact to develop and up-skill in order to teach people about echolocation is absolutely fantastic.”*

“*It has given me a significant new aspect upon which to draw and which to provide for my clients for instruction. It is such a valuable tool which is, and will continue to be, so useful from now on. This is because your course gave me the confidence to try the techniques out myself. When I saw the success from them in such a short time this also gave confidence by which to pass the techniques on to clients. Thank you.”*

*“I am currently working with a child with almost total vision loss and have started introducing the concept of echo location, this term some unexpected issues have arisen which have put the training back but she has shown some great results with early sessions. It has also raised my awareness again of echo location as a transferrable skill into other areas of life for the VI children we work with.”*

*“I have now for some years worked with some pupils who can do this skill already. But been told not to practice it nor write about it on my mobility reports. However, since doing the short course I now practice this skill with my VI students. I really enjoyed the course and found it very useful as it enlightened me to the fact that it should be delivered as part of a mobility lesson if a student wishes to do this skill or learn how to do it.”*

*“I would say the training has impacted already on my professional work. I have several young people who are blind and severely visually impaired. My awareness of gauging space is different now that I have received the training and it is easy to get the very young children to hear my click and cross a room successfully. They seem to know where the wall is and reach out to the wall within arms reach—even though they have no sight. They cannot make the click yet (this could be my training!) however they are only 4 years old so maybe this is too young anyway? So I make the click for them when I stand next to them. It would be good to do some follow up training maybe next year or in xxxx to further improve my skills. By this time the younger children I am currently working with will need to be clicking themselves—and therefore a refresher and further information would be very useful. Thank you again for the training—it is an excellent part of my toolkit I now use each week.”*

*“The training helped with ideas for activities to develop the echo location skills. It gave me more confidence to incorporate the techniques during my mobility sessions.”*

The group data are visually summarized in Figure 3B.

## Discussion

Previous research has investigated echolocation in its own right in order to understand how echolocation works and its neural underpinnings [for reviews see (1–3, 13)]. Previous research has also shown that use of and training in click-based echolocation, alongside other mobility methods such as long cane or guide dog for example, is associated with increased mobility, independence and wellbeing in people with VI (6, 8).

Even though use of click-based echolocation is currently still scarce, it is gaining traction as a tool to improve independent mobility, including in low-income settings (6, 9), but there is room for increasing access to and usage of this skill in people with VI. VI professionals are well placed to affect how a person with VI might learn about, experience or use click-based echolocation. Consequently, our report investigated if training in click-based echolocation for VI professionals might lead to a change in their professional practice, i.e., our report has a different focus from previous work in click-based echolocation.

We found that participation in a 6-h workshop consisting of lecture and practical exercises in click-based echolocation led to a reported change in professional practice in nearly the entire group of VI professionals who responded to our feedback questions, i.e., 98%. Free text comments further indicated that the training had led to changes in the way people provide instruction and training in almost half of all respondents (46.6%), whilst changes in the way people process information was observed in 32% and changes in verbal influencing were observed in 11.7%. One may speculate that with a longer workshop, or more than one workshop, an even higher proportion of respondents may report changes in instruction and training.

The training we provided was in echolocation, covering both theory and practical exercises. We had asked attendees how the training had affected their professional practice. That is, the e-mail we had sent contained two specific questions. The first question read “Has what you learnt during the training affected your professional practice (e.g., better understanding, better awareness, making recommendations, instruction, etc.)? YES/NO”. The second question read “If yes, in your own words how has it affected your professional practice?”. Thus, we asked people specifically about the effects of having attended our training on their practice. Importantly, these questions did not mention echolocation specifically. On the other hand, the coding scheme we applied was specific to changes in professional practice for echolocation (see Table 1). Thus, the results we obtained are specific to the training we had provided and specific to changes in professional practice with respect to echolocation.

The overall number of people who attended our workshops was 201 VI professionals. At the workshop, people opted in to be contacted for follow-up, and there was no pressure or incentive to respond. Looking at numbers of people who opted in to be contacted (*n* = 166), as well as response numbers (*n* = 105), there is no indication that people felt pressure to respond. But, it is likely that self-selection bias, or selective attrition, are relevant, as in any type of research that collects data in this way. Thus, it is possible that people who did not respond to our feedback or did not opt-in to be contacted, would have all answered “no” to the question if there had been a change in their professional practice. Thus, the overall effectiveness of 6-h workshops might be lower, and it is possible that it would only affect 103 out of 201 or 51% of attendees.

The change in professional practice that we found in VI professionals, in particular the reported changes with respect to training and instruction is expected to translate into benefits for people with VI. We suggest that research investigating the effects that changes in professional practice have on clients, i.e., people with VI, would be a fruitful avenue for further research.

## Conclusion

The training we evaluated here, i.e., 6-h workshop, split into lecture, Q&A and practical exercises, could feasibly be integrated into VI training as implemented at HEIs or CPD. We advise that future studies should investigate the effects that changes in professional practice have on clients, i.e., people with VI.

## Data Availability

The original contributions presented in the study are included in the article/**Supplementary Material**, further inquiries can be directed to the corresponding author/s.
