# Supplementary material for: 6-hour Training in click-based echolocation changes practice in visual impairment professionals
Source: Front Rehabil Sci. 2023 May 22;4:1098624. doi: 10.3389/fresc.2023.1098624 (PMC10239887; doi:10.3389/fresc.2023.1098624)
Supplement: Supplementary file 2 [file Presentation1.pptx]

## Slide 1
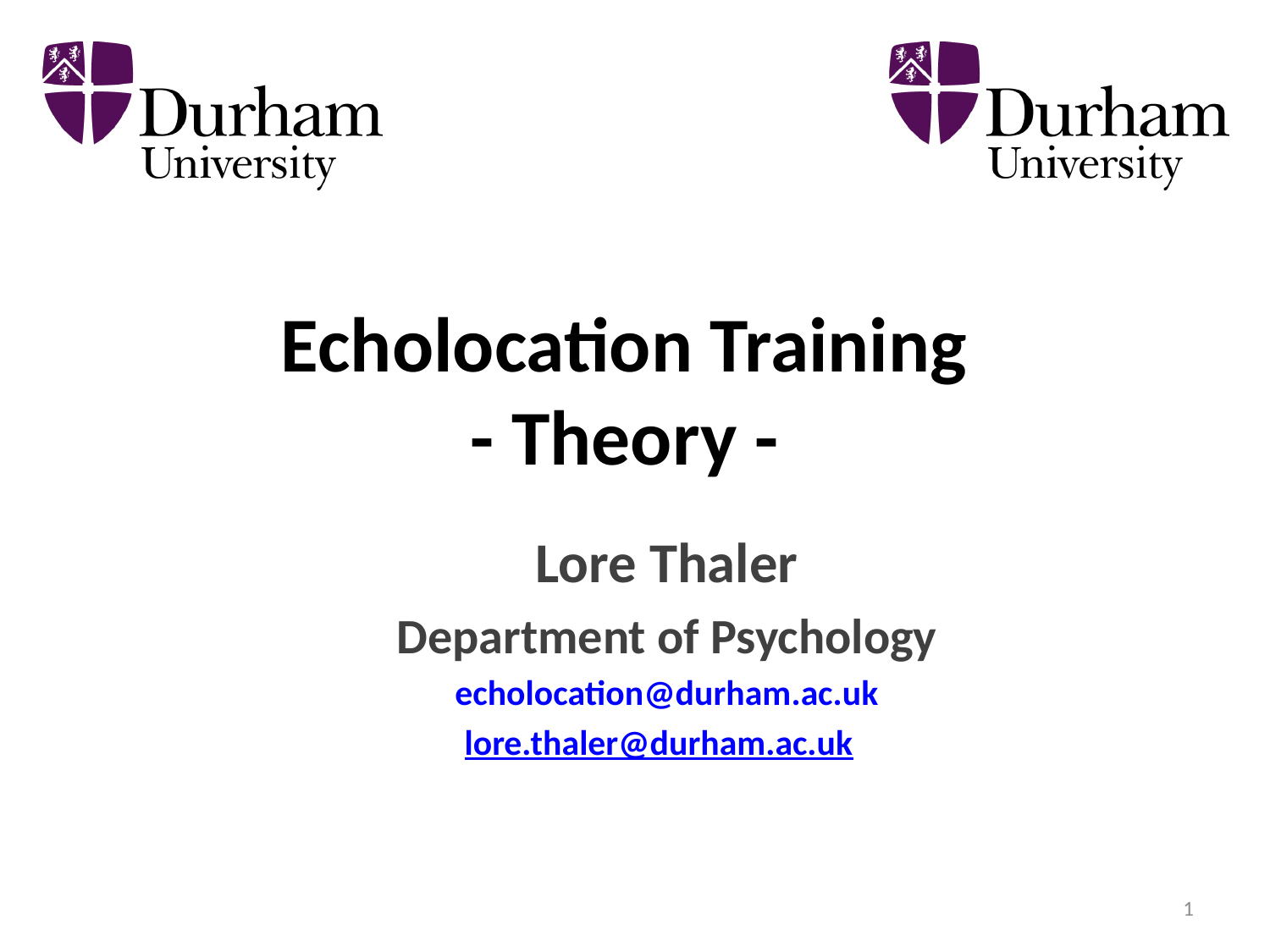

# Echolocation Training - Theory -
Lore Thaler
Department of Psychology
echolocation@durham.ac.uk
lore.thaler@durham.ac.uk
1

## Slide 2
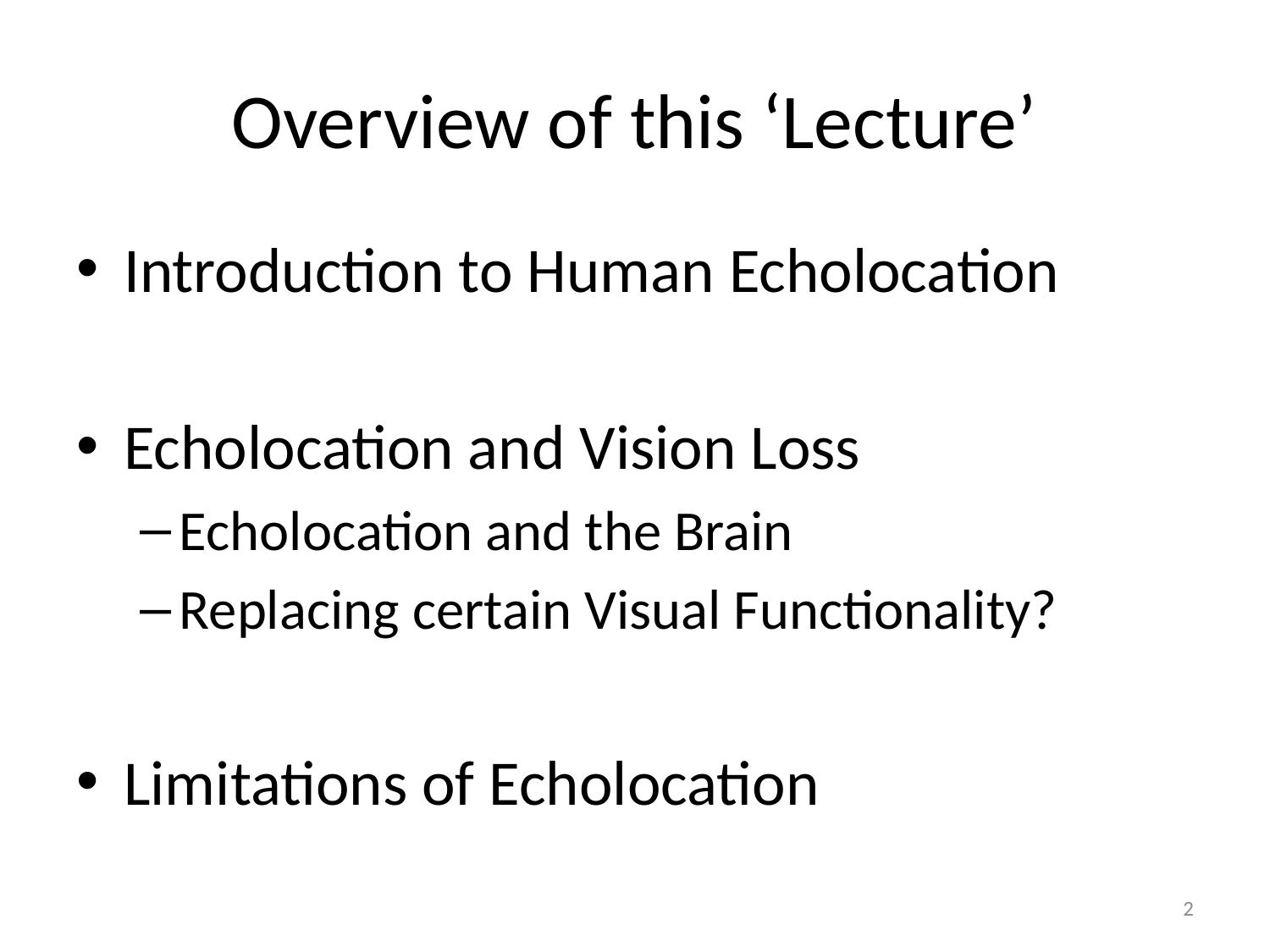

# Overview of this ‘Lecture’
Introduction to Human Echolocation
Echolocation and Vision Loss
Echolocation and the Brain
Replacing certain Visual Functionality?
Limitations of Echolocation
2

## Slide 3
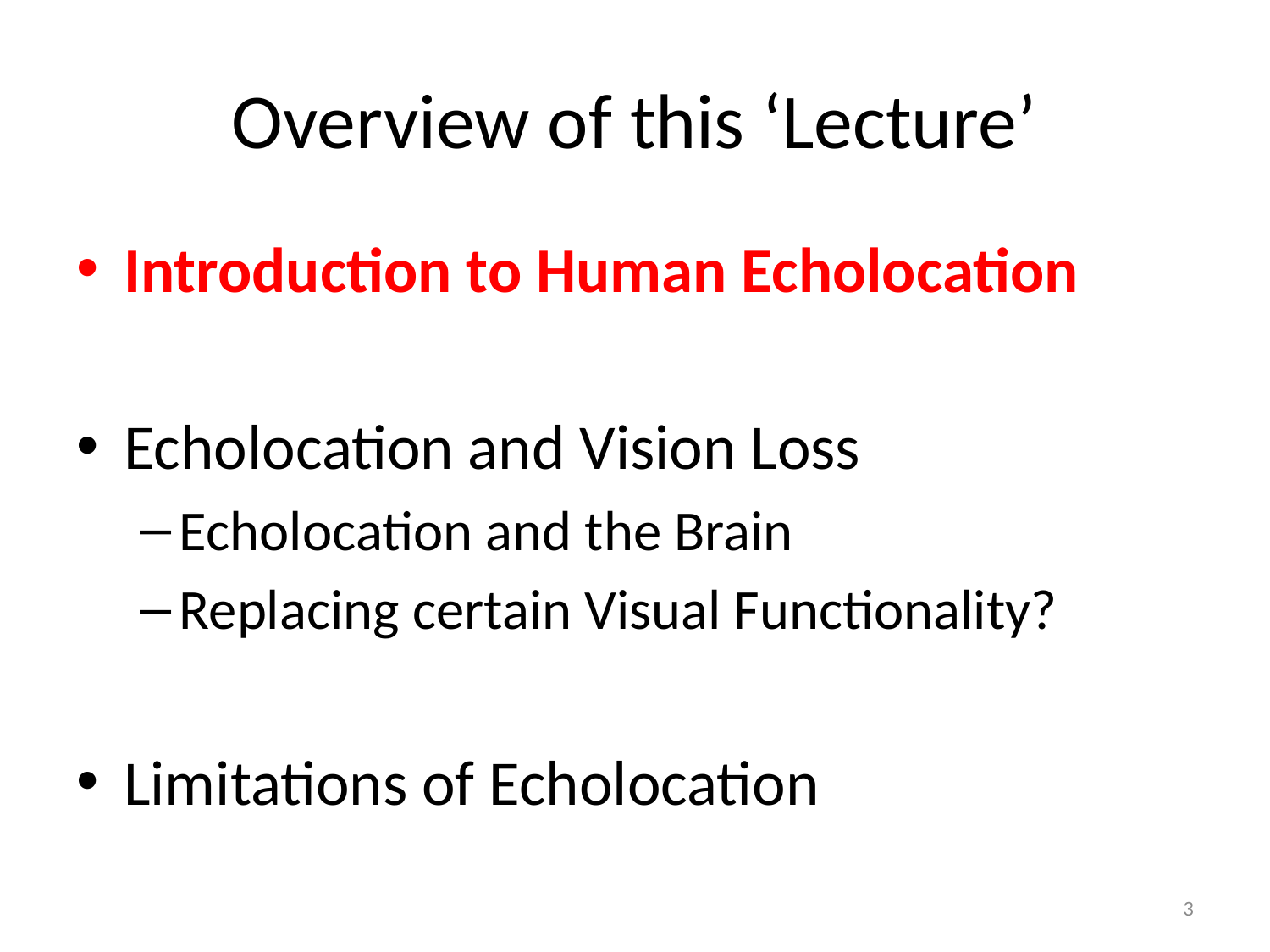

# Overview of this ‘Lecture’
Introduction to Human Echolocation
Echolocation and Vision Loss
Echolocation and the Brain
Replacing certain Visual Functionality?
Limitations of Echolocation
3

## Slide 4
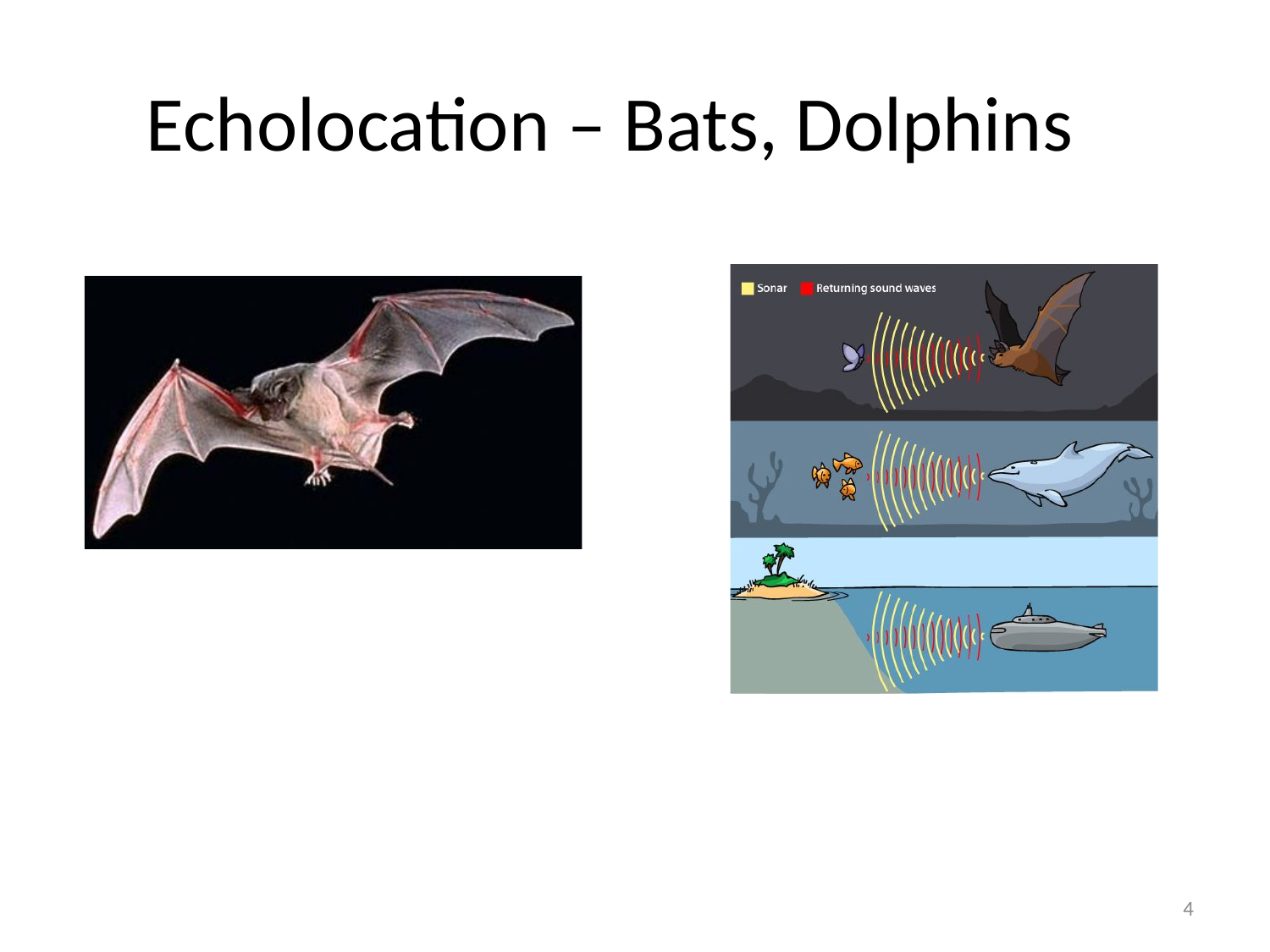

Echolocation – Bats, Dolphins … People
#
4

## Slide 5
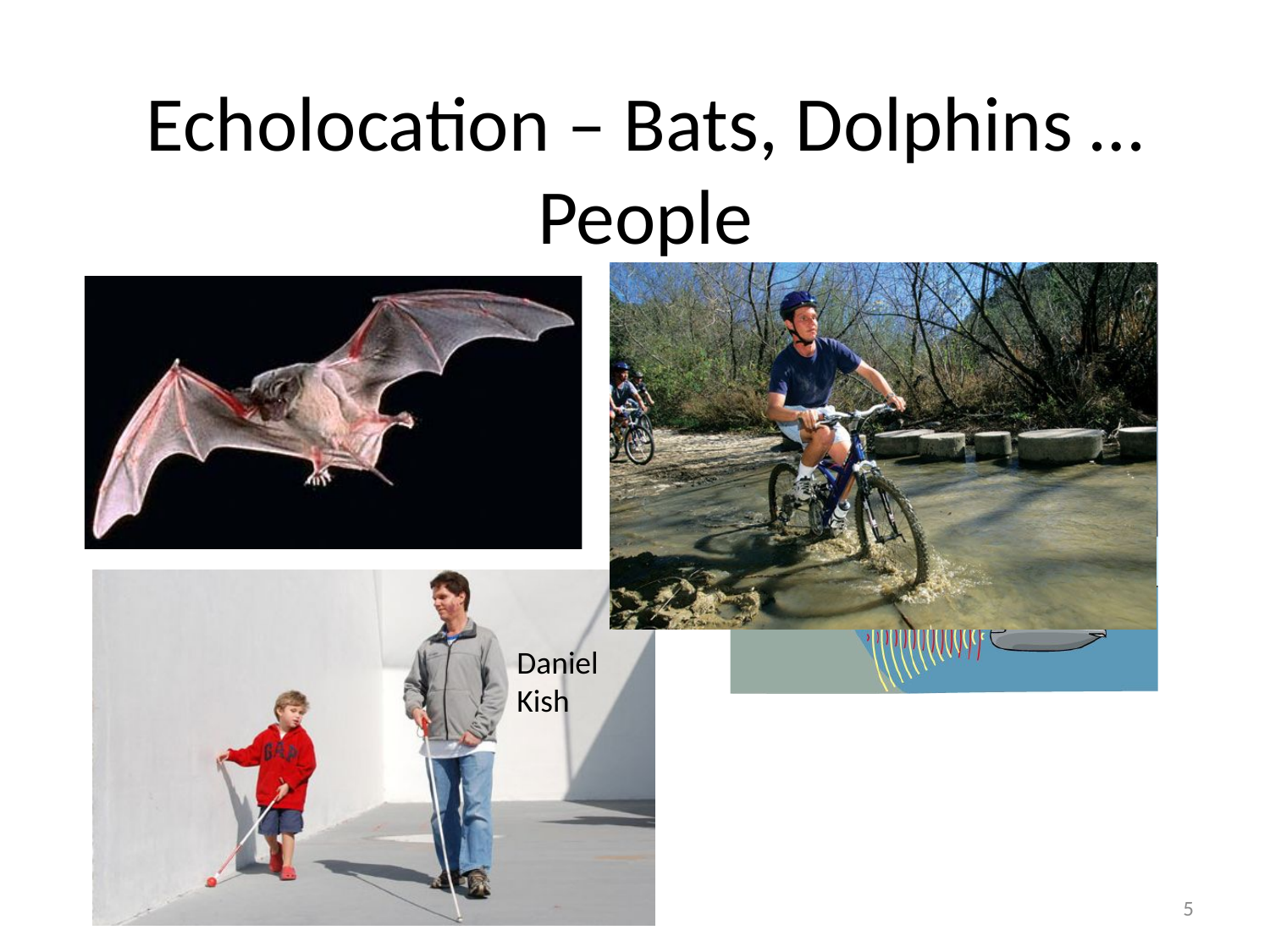

Echolocation – Bats, Dolphins … People
#
Daniel Kish
5

## Slide 6
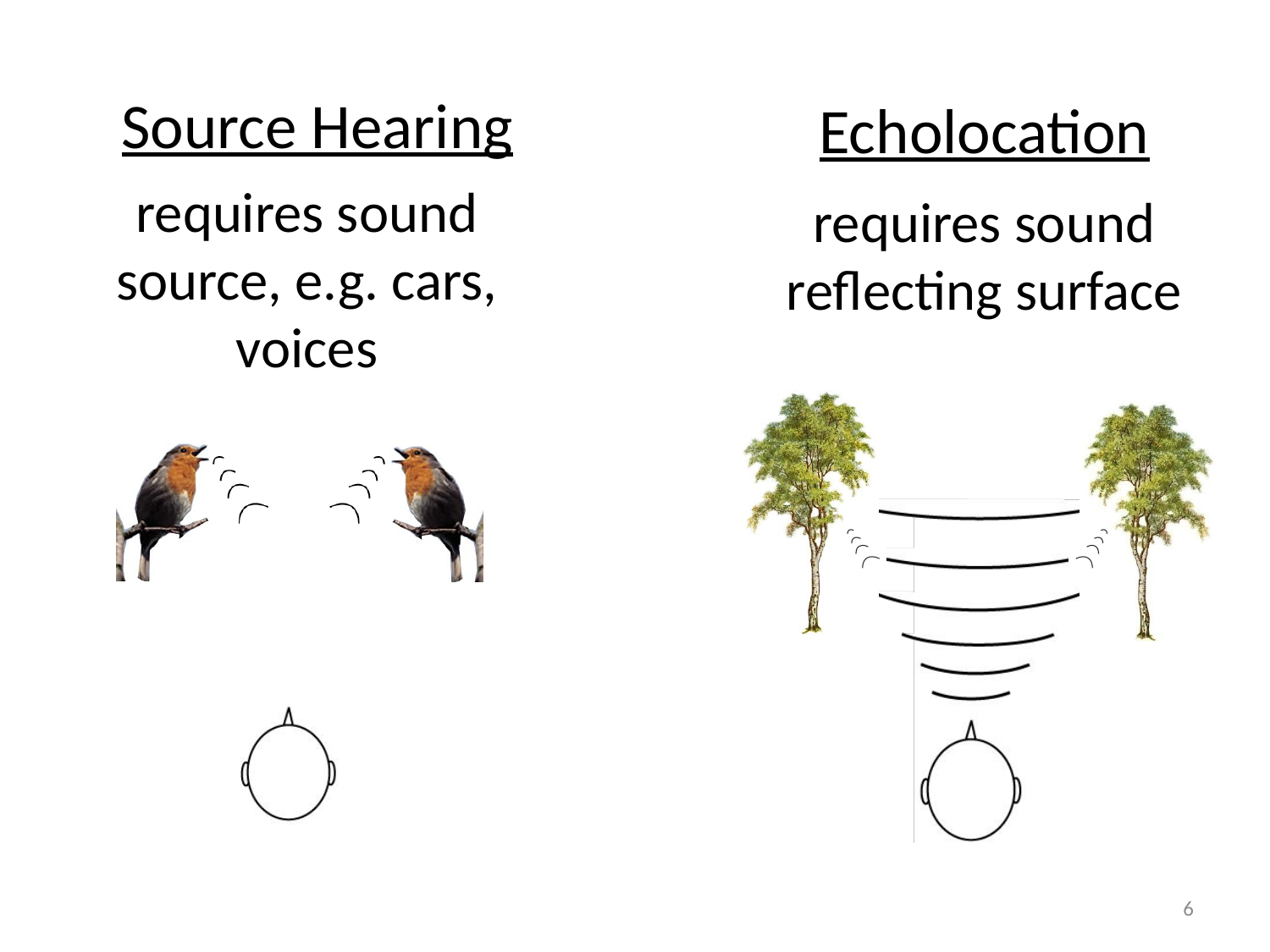

# Source Hearing
Echolocation
requires sound source, e.g. cars, voices
requires sound reflecting surface
6

## Slide 7
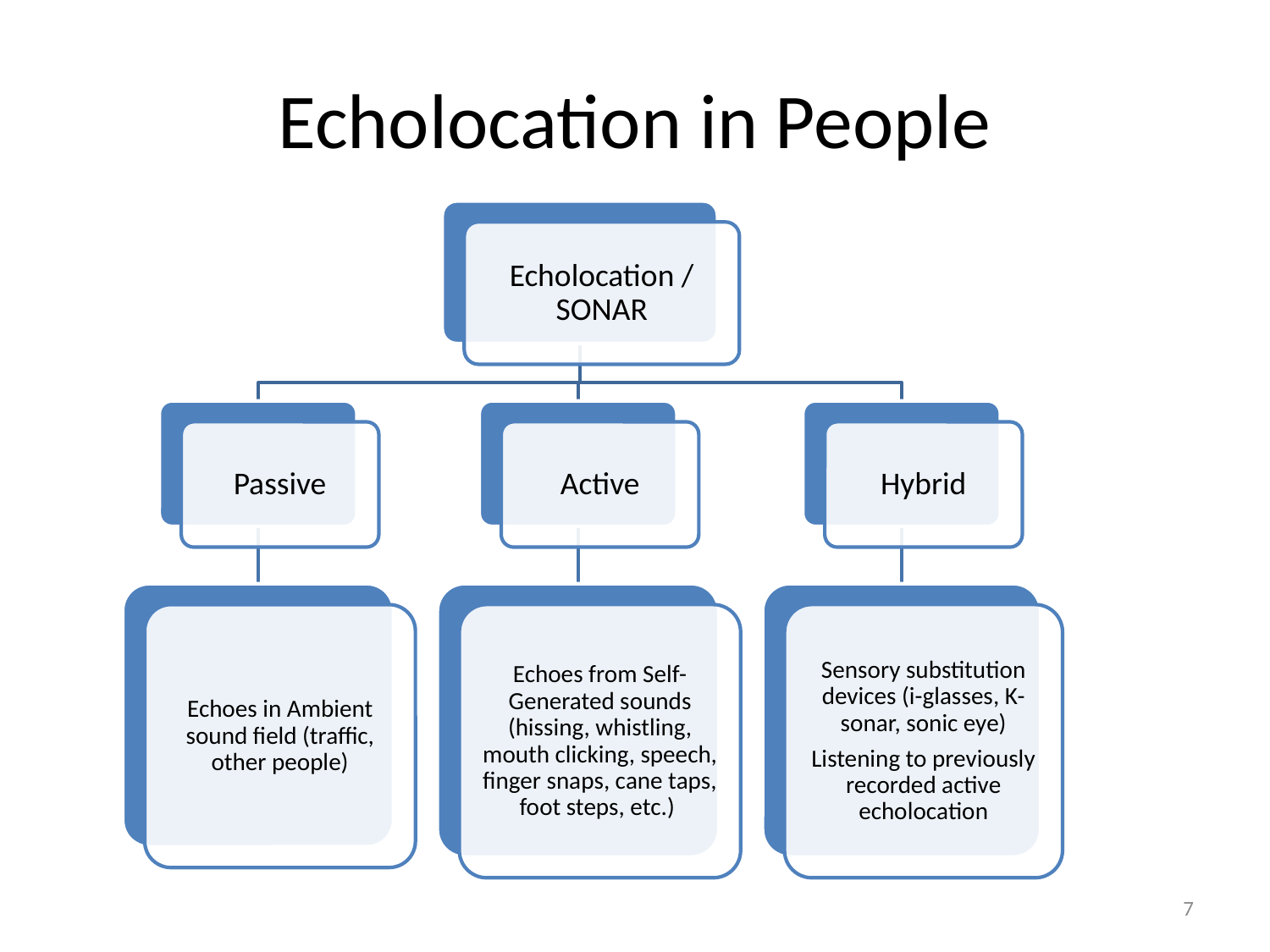

# Echolocation in People
7

## Slide 8
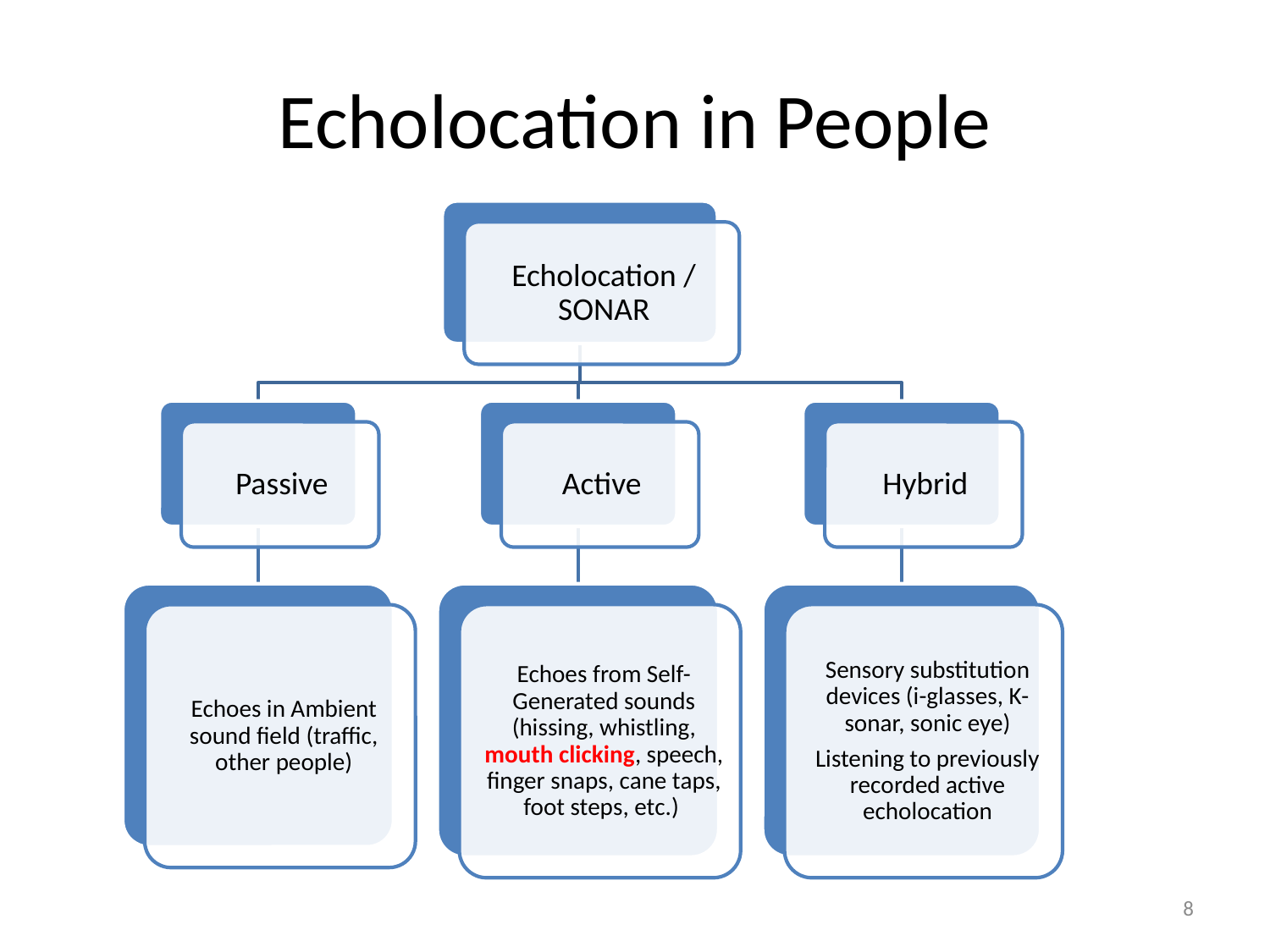

# Echolocation in People
8

## Slide 9
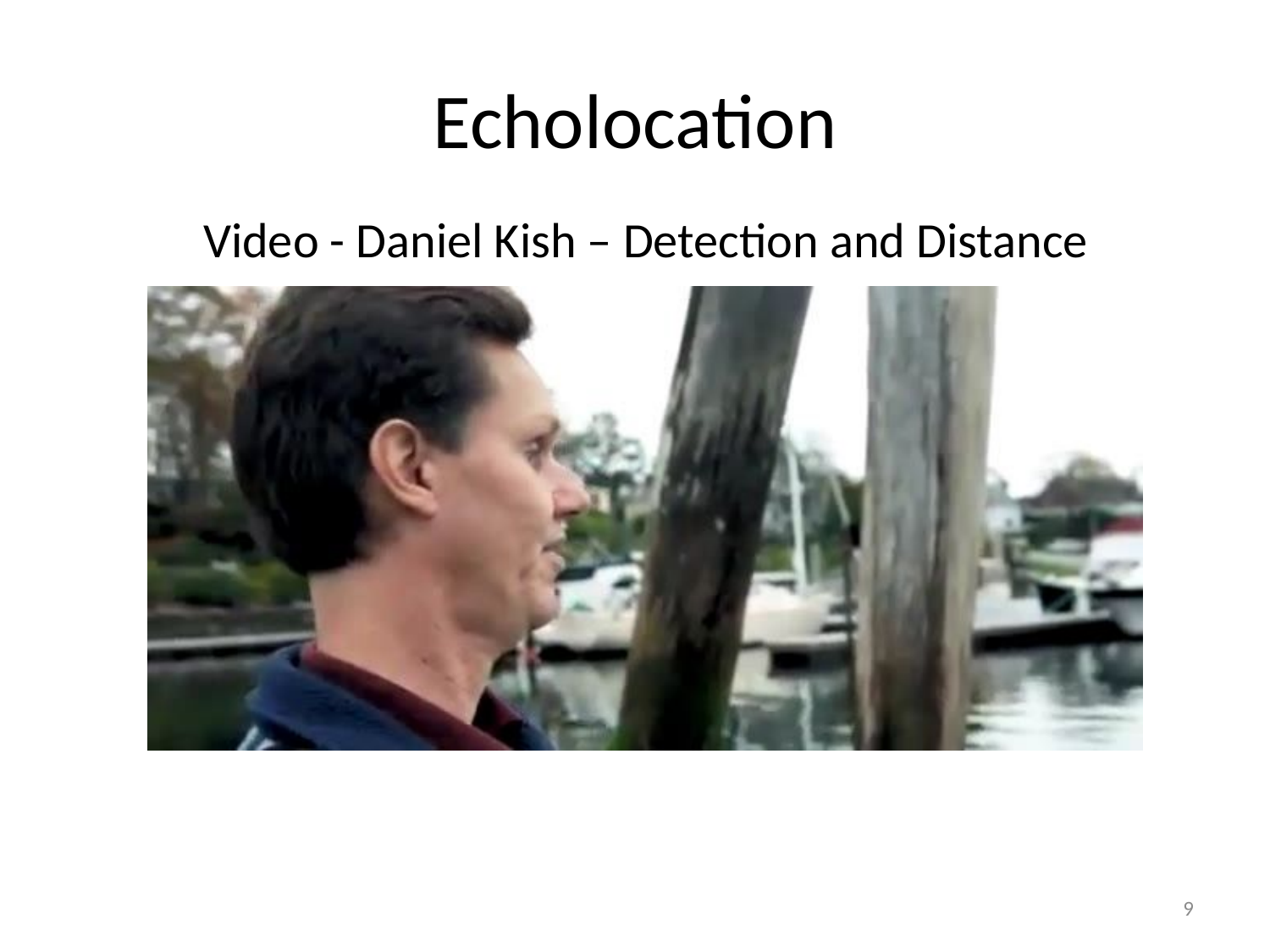

# Echolocation
Video - Daniel Kish – Detection and Distance
9

## Slide 10
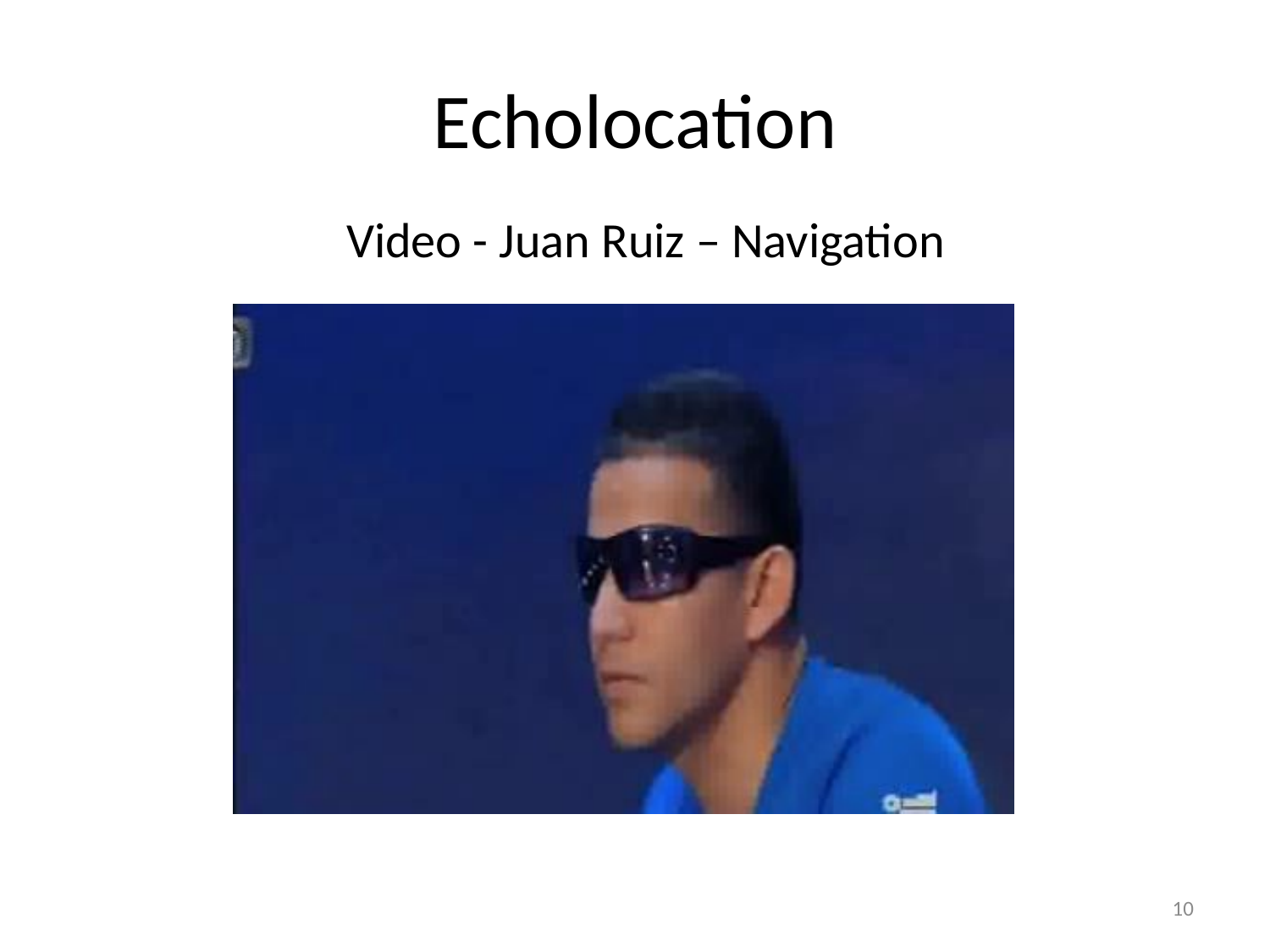

#
Echolocation
Video - Juan Ruiz – Navigation
10

## Slide 11
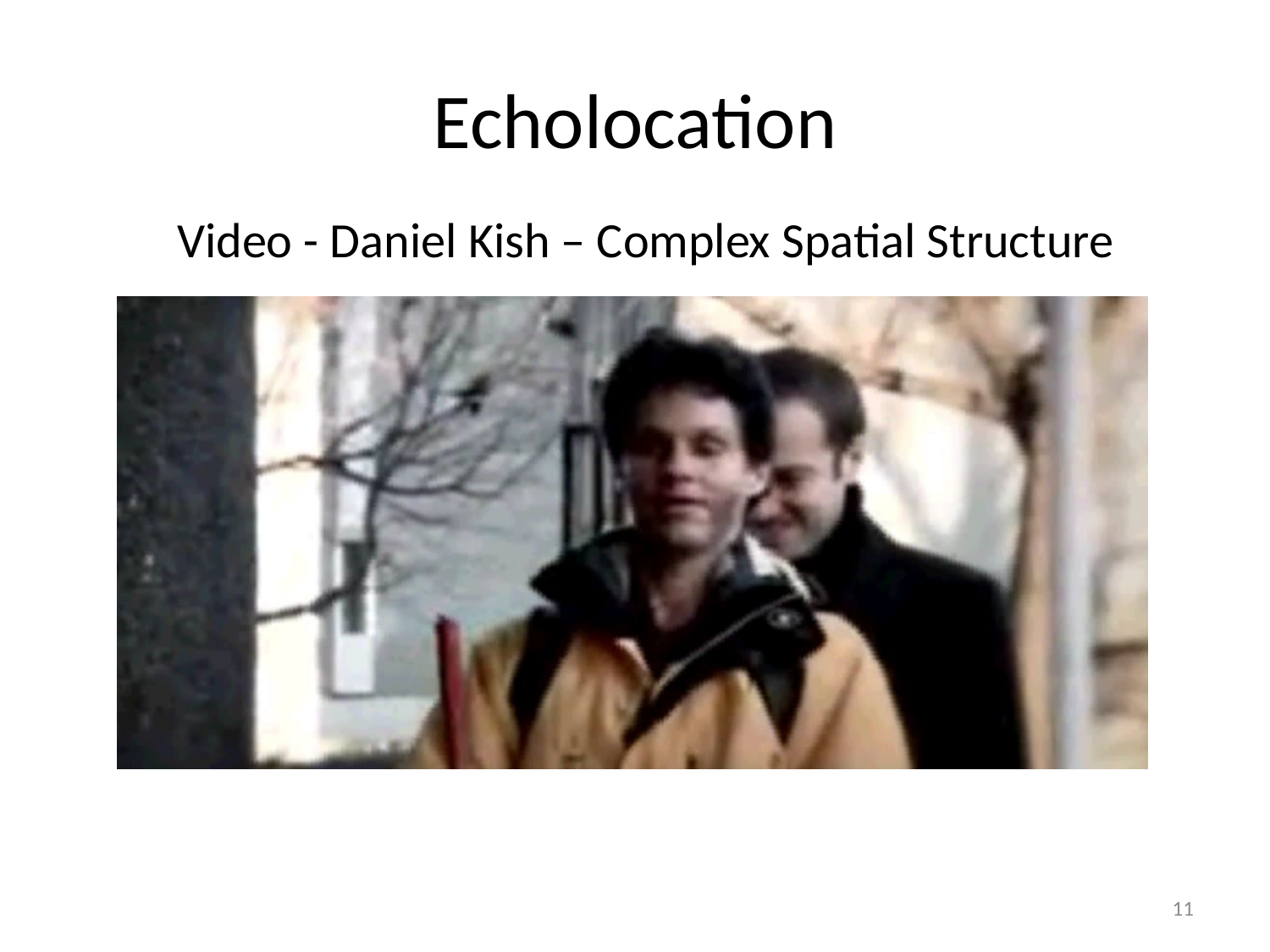

# Echolocation
Video - Daniel Kish – Complex Spatial Structure
11

## Slide 12
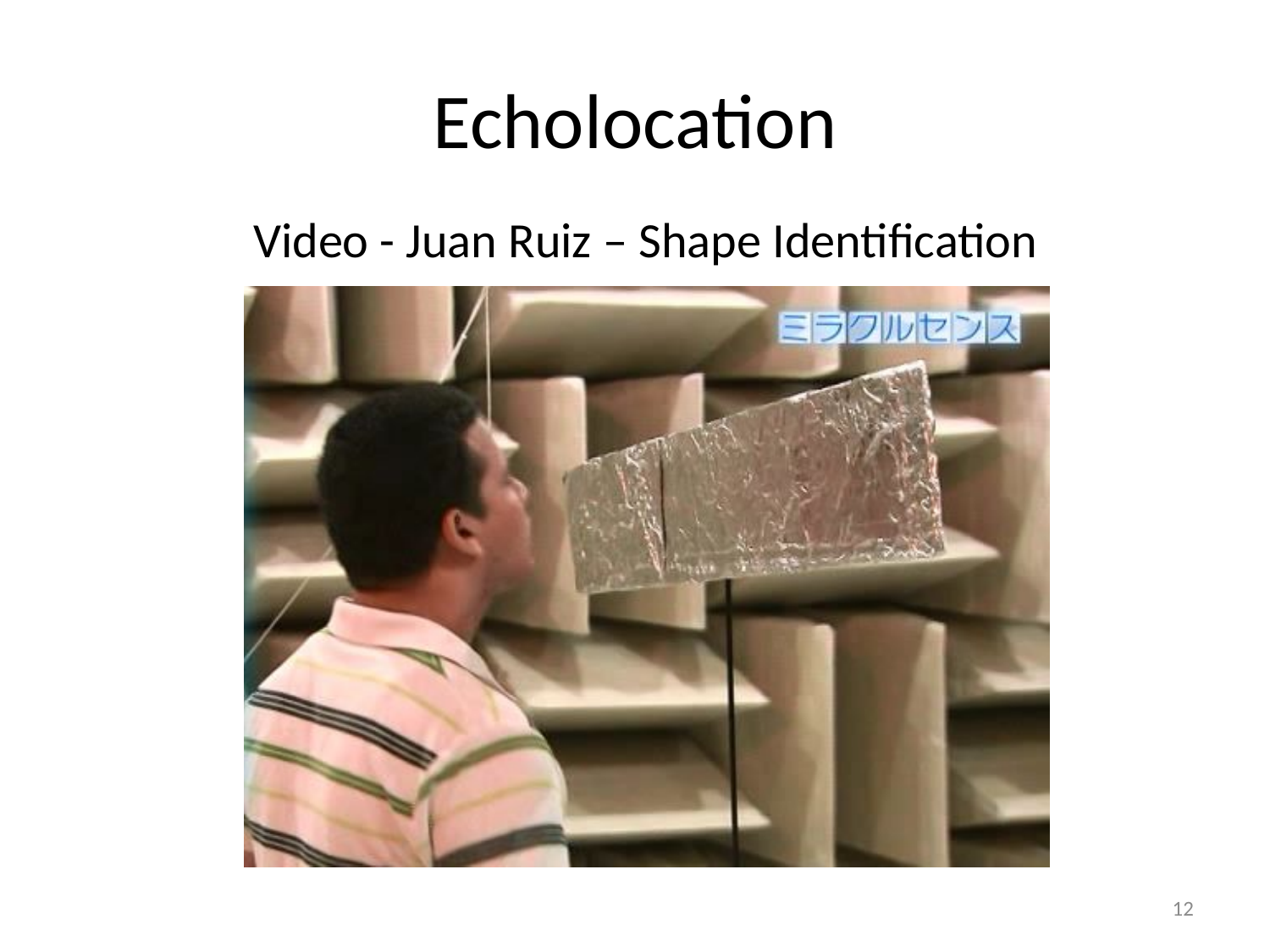

# Echolocation
Video - Juan Ruiz – Shape Identification
12

## Slide 13
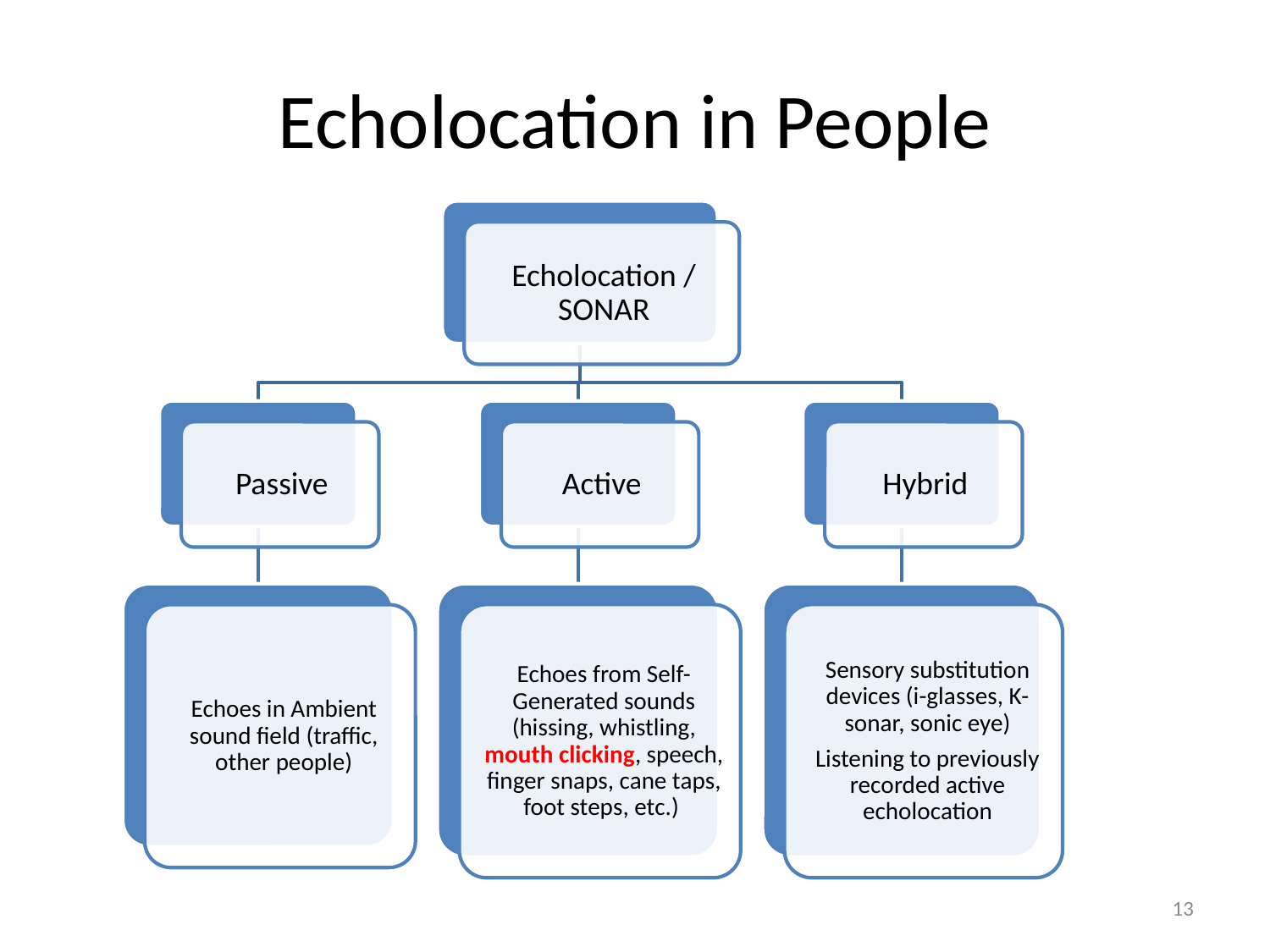

# Echolocation in People
13

## Slide 14
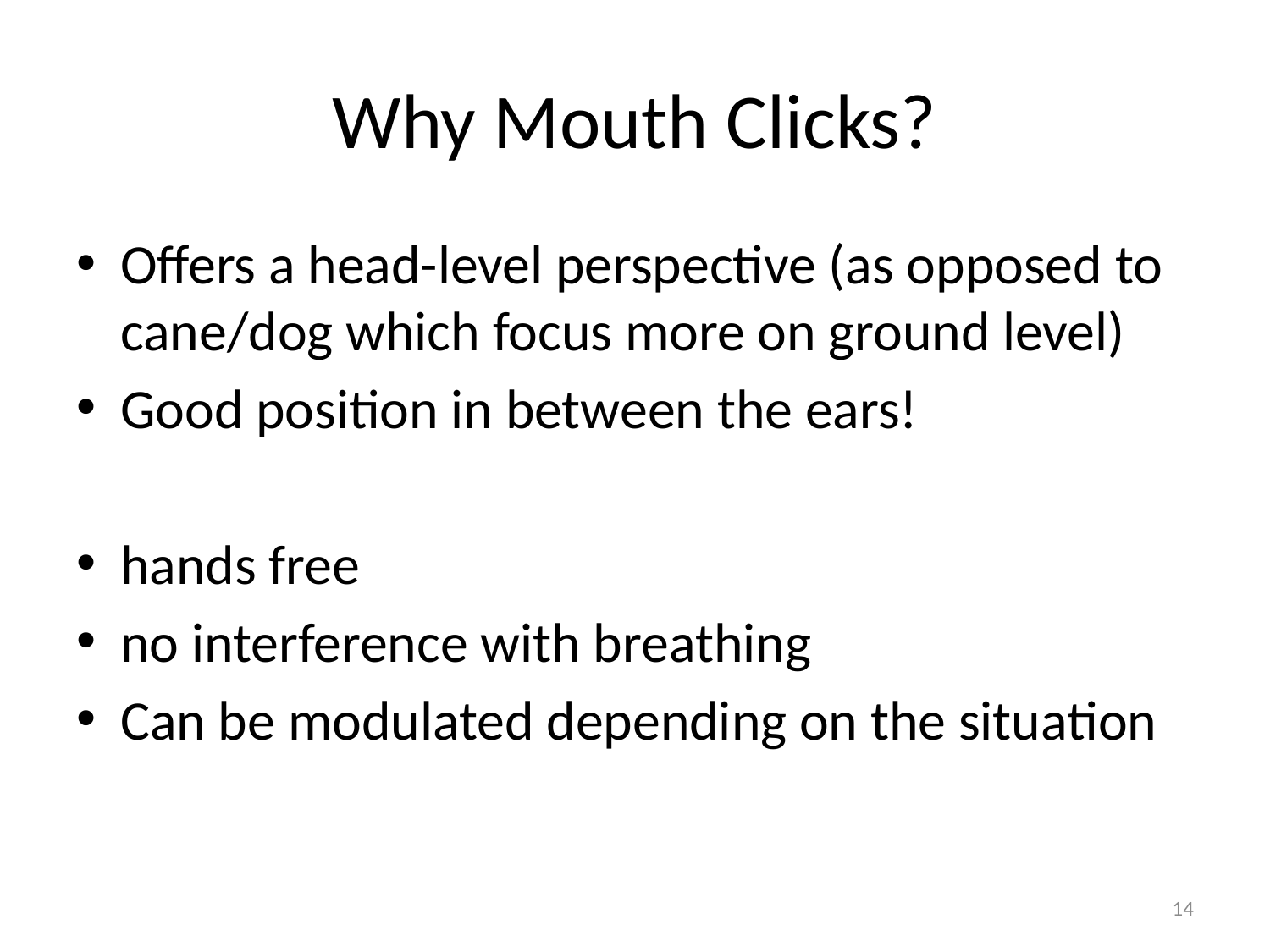

# Why Mouth Clicks?
Offers a head-level perspective (as opposed to cane/dog which focus more on ground level)
Good position in between the ears!
hands free
no interference with breathing
Can be modulated depending on the situation
14

## Slide 15
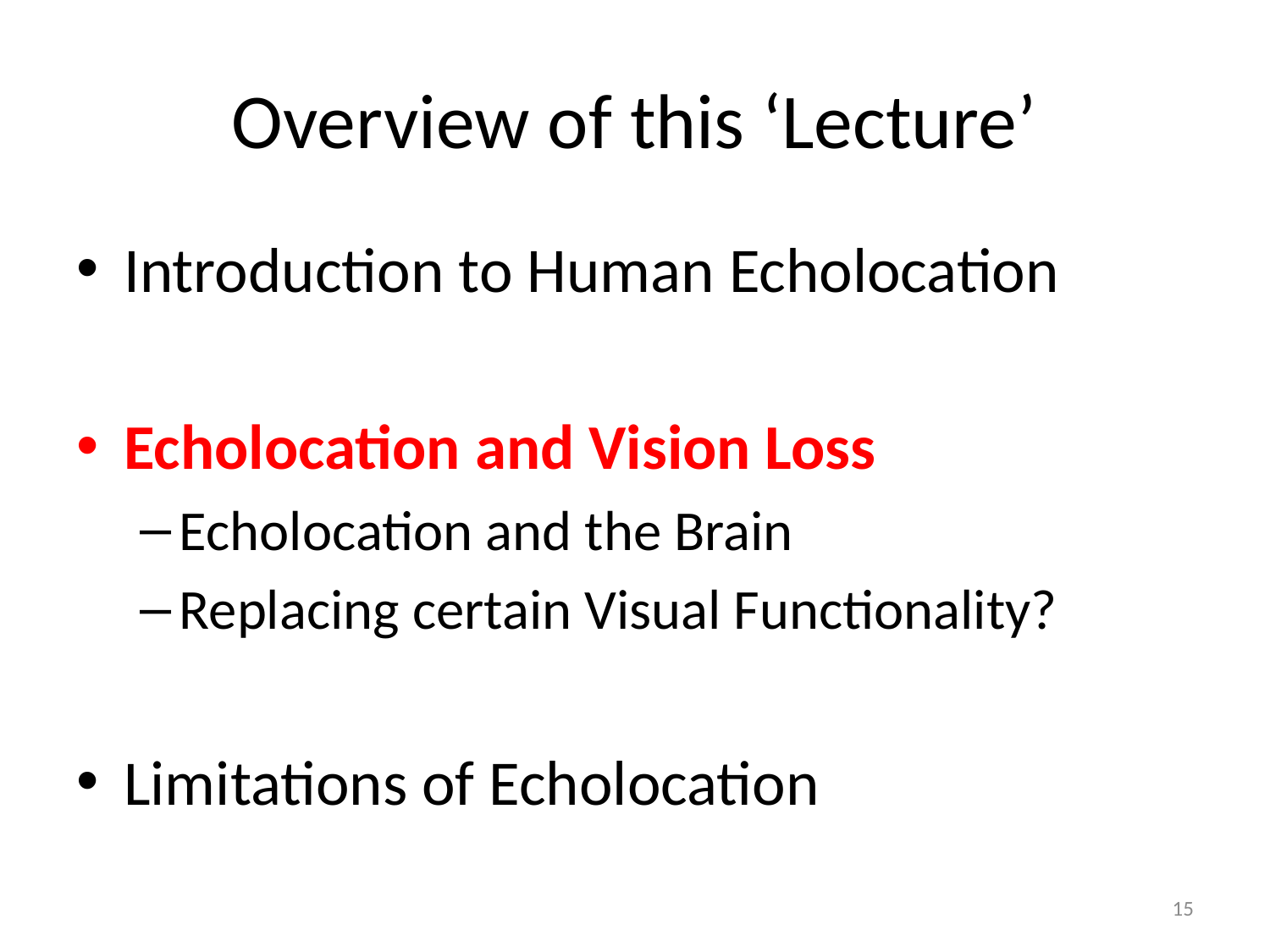

# Overview of this ‘Lecture’
Introduction to Human Echolocation
Echolocation and Vision Loss
Echolocation and the Brain
Replacing certain Visual Functionality?
Limitations of Echolocation
15

## Slide 16
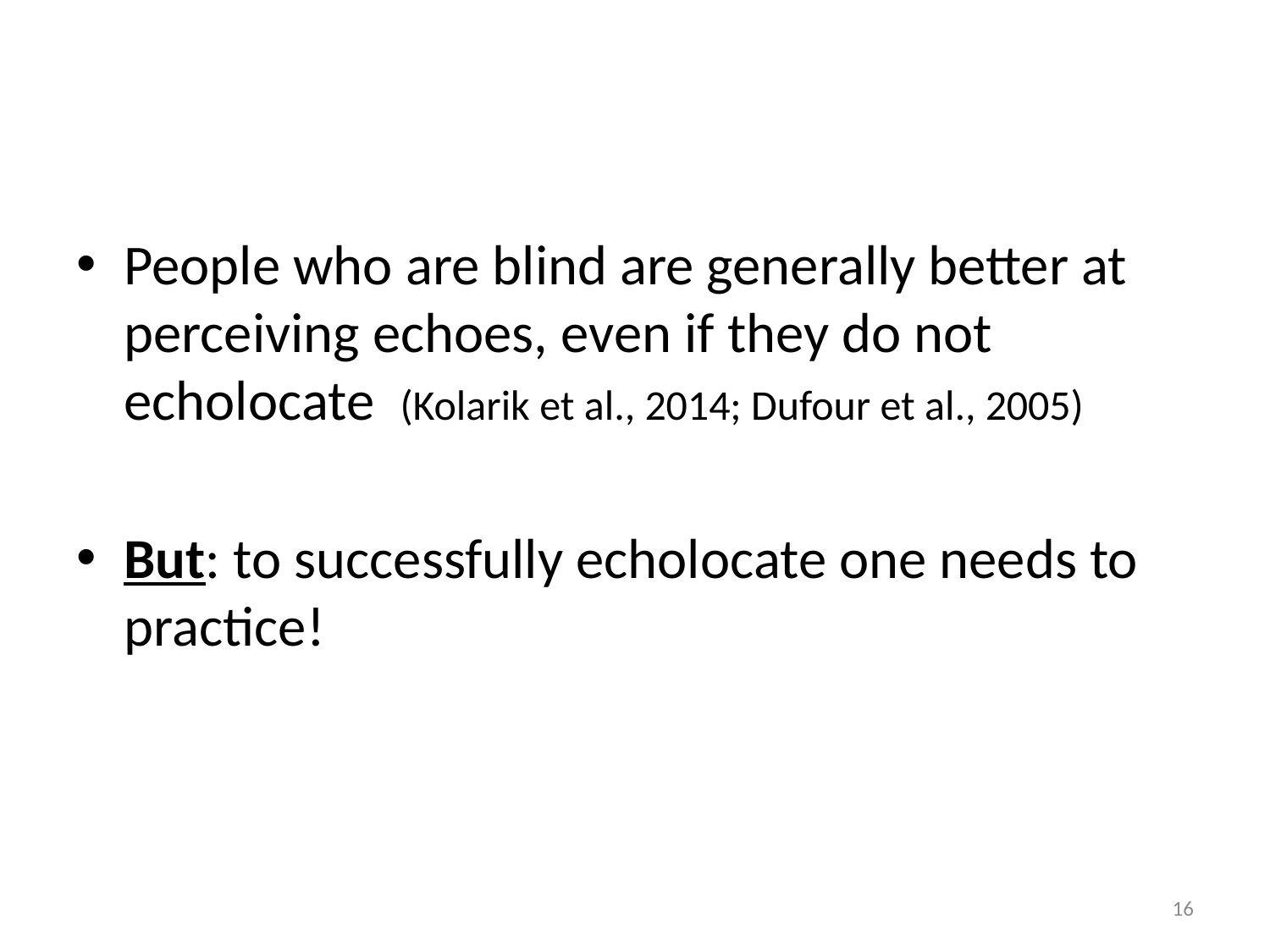

#
People who are blind are generally better at perceiving echoes, even if they do not echolocate (Kolarik et al., 2014; Dufour et al., 2005)
But: to successfully echolocate one needs to practice!
16

## Slide 17
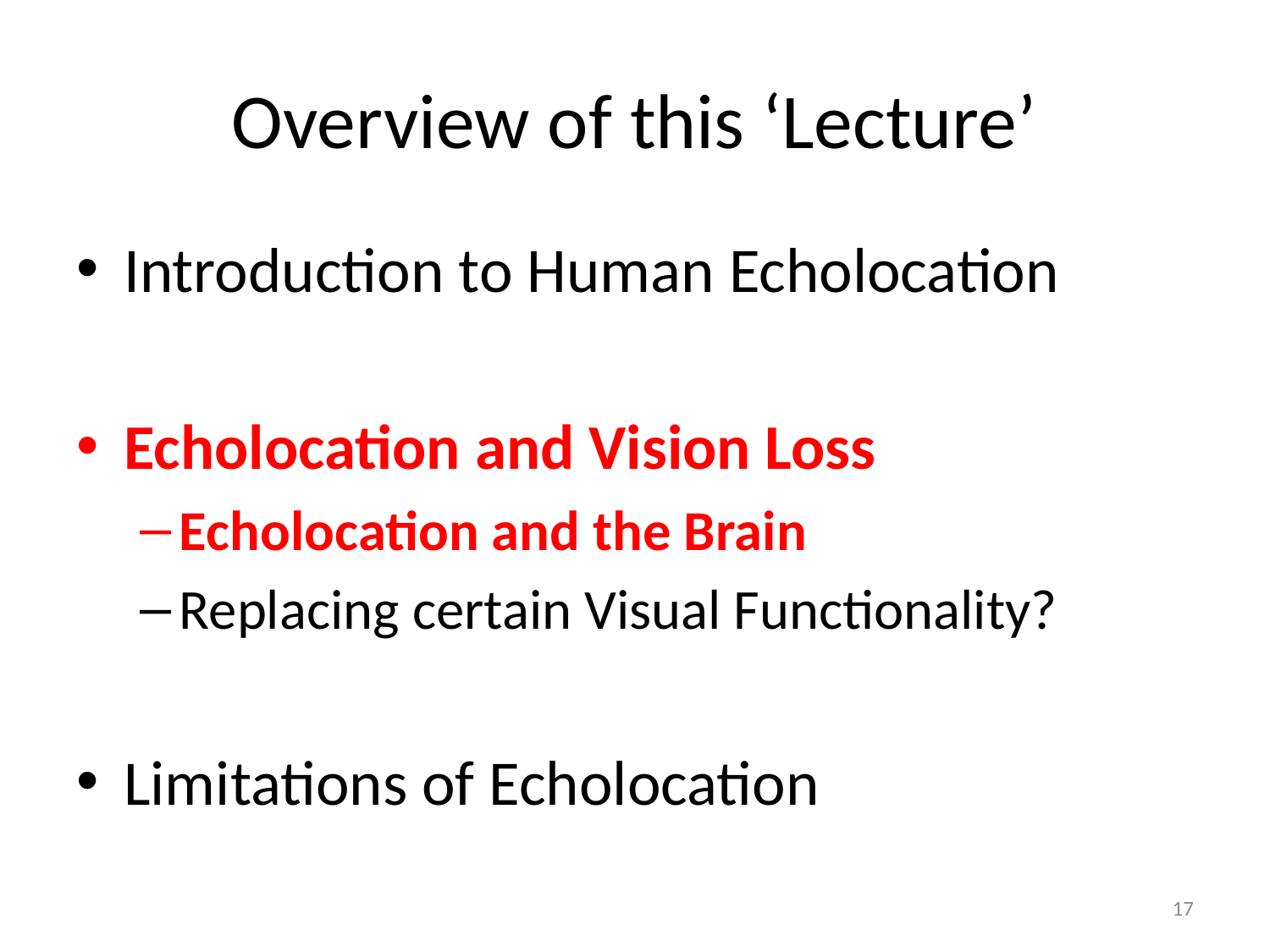

# Overview of this ‘Lecture’
Introduction to Human Echolocation
Echolocation and Vision Loss
Echolocation and the Brain
Replacing certain Visual Functionality?
Limitations of Echolocation
17

## Slide 18
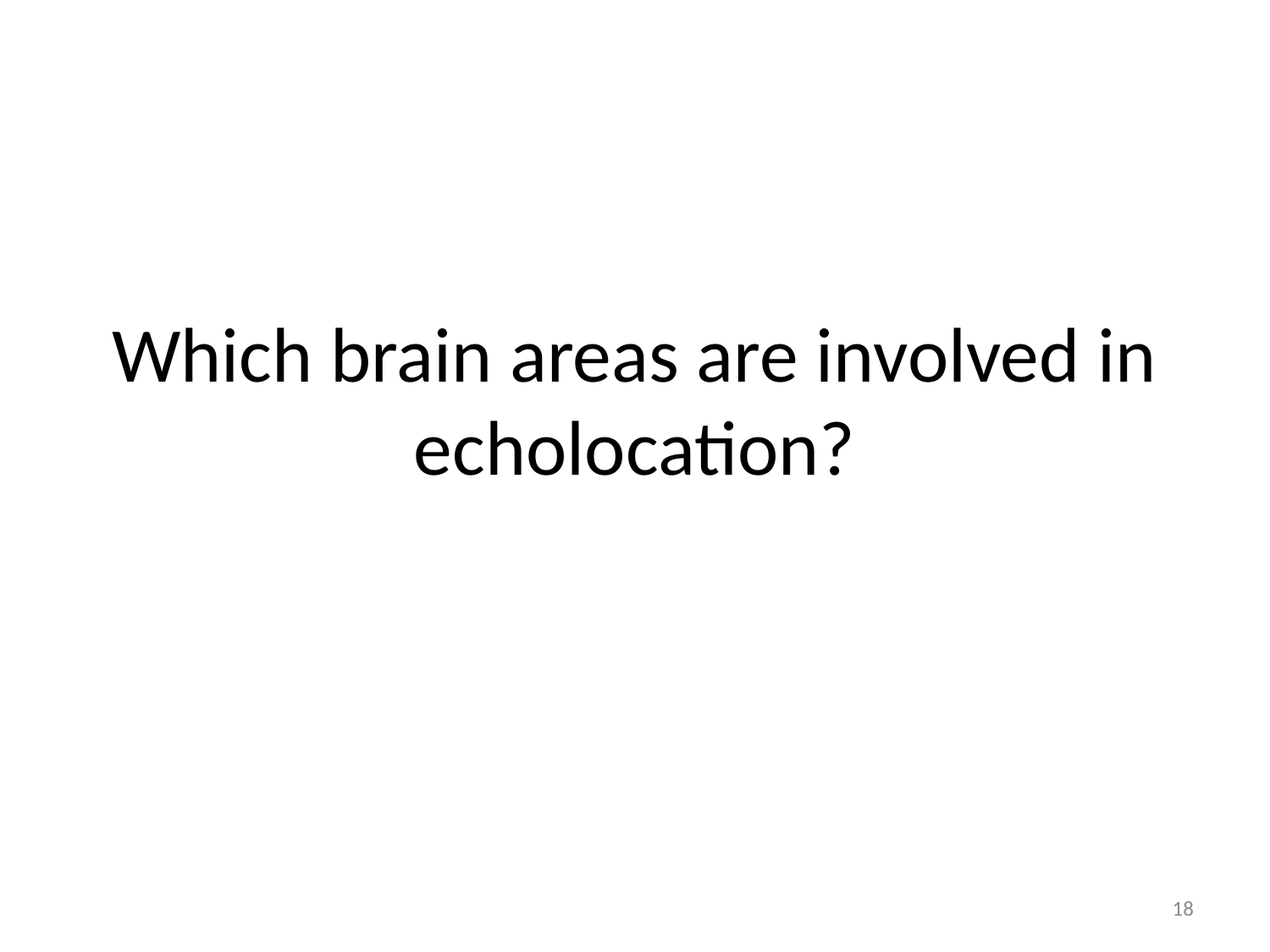

# Which brain areas are involved in echolocation?
18

## Slide 19
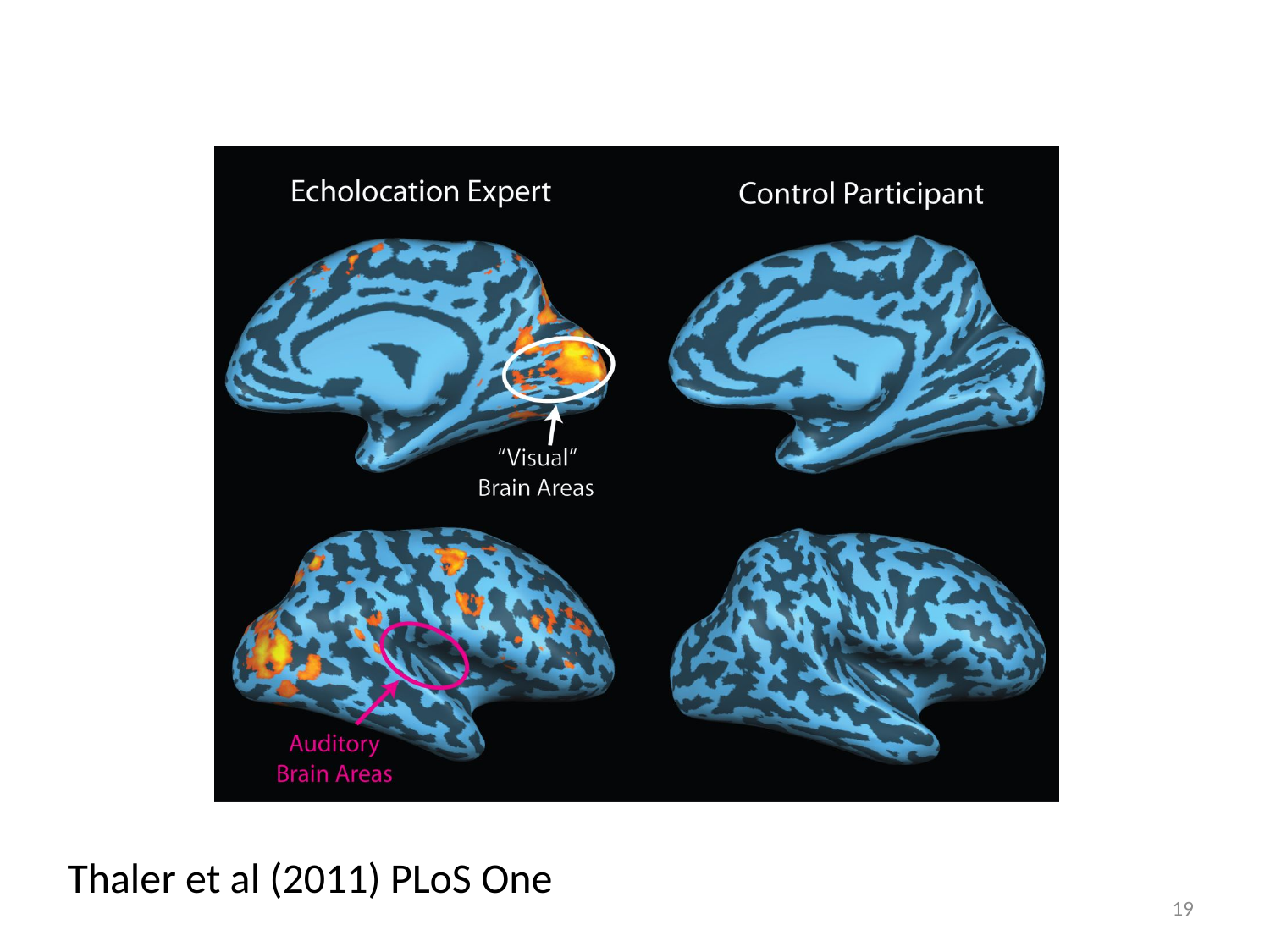

Thaler et al (2011) PLoS One
19

## Slide 20
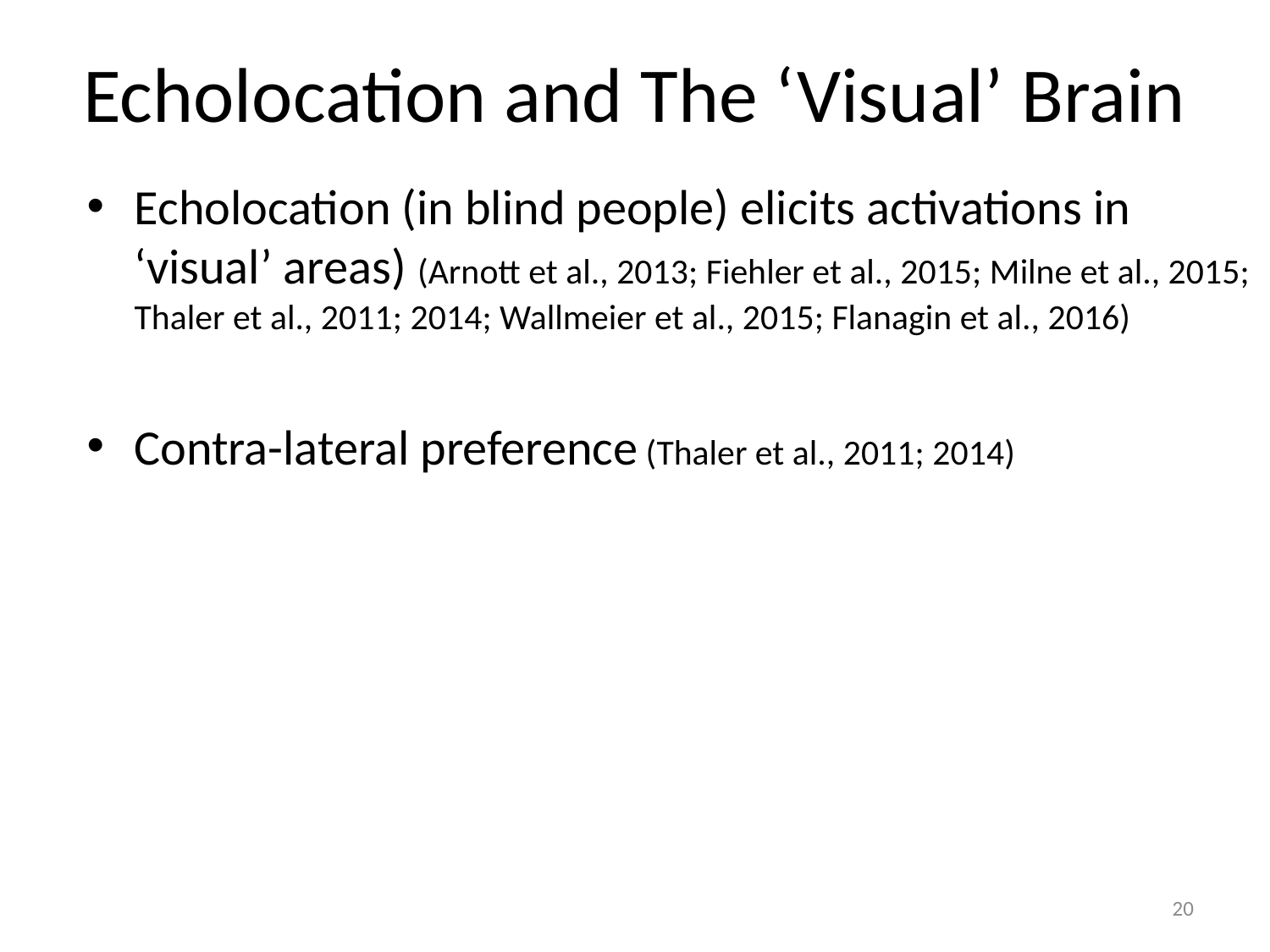

Echolocation and The ‘Visual’ Brain
Echolocation (in blind people) elicits activations in ‘visual’ areas) (Arnott et al., 2013; Fiehler et al., 2015; Milne et al., 2015; Thaler et al., 2011; 2014; Wallmeier et al., 2015; Flanagin et al., 2016)
Contra-lateral preference (Thaler et al., 2011; 2014)
20

## Slide 21
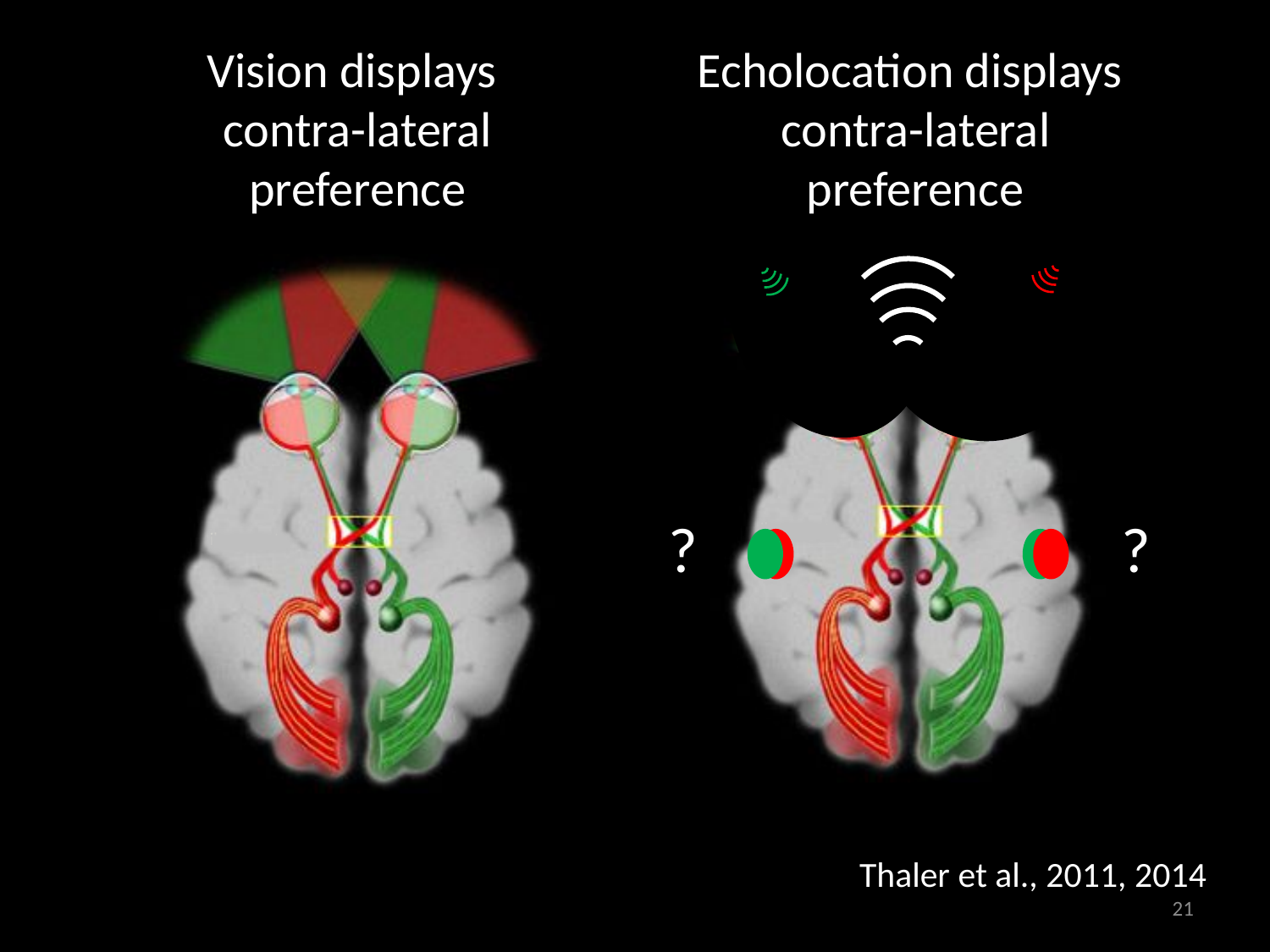

Vision displays
contra-lateral preference
Echolocation displays
contra-lateral preference
iknow.net
iknow.net
?
?
Thaler et al., 2011, 2014
21

## Slide 22
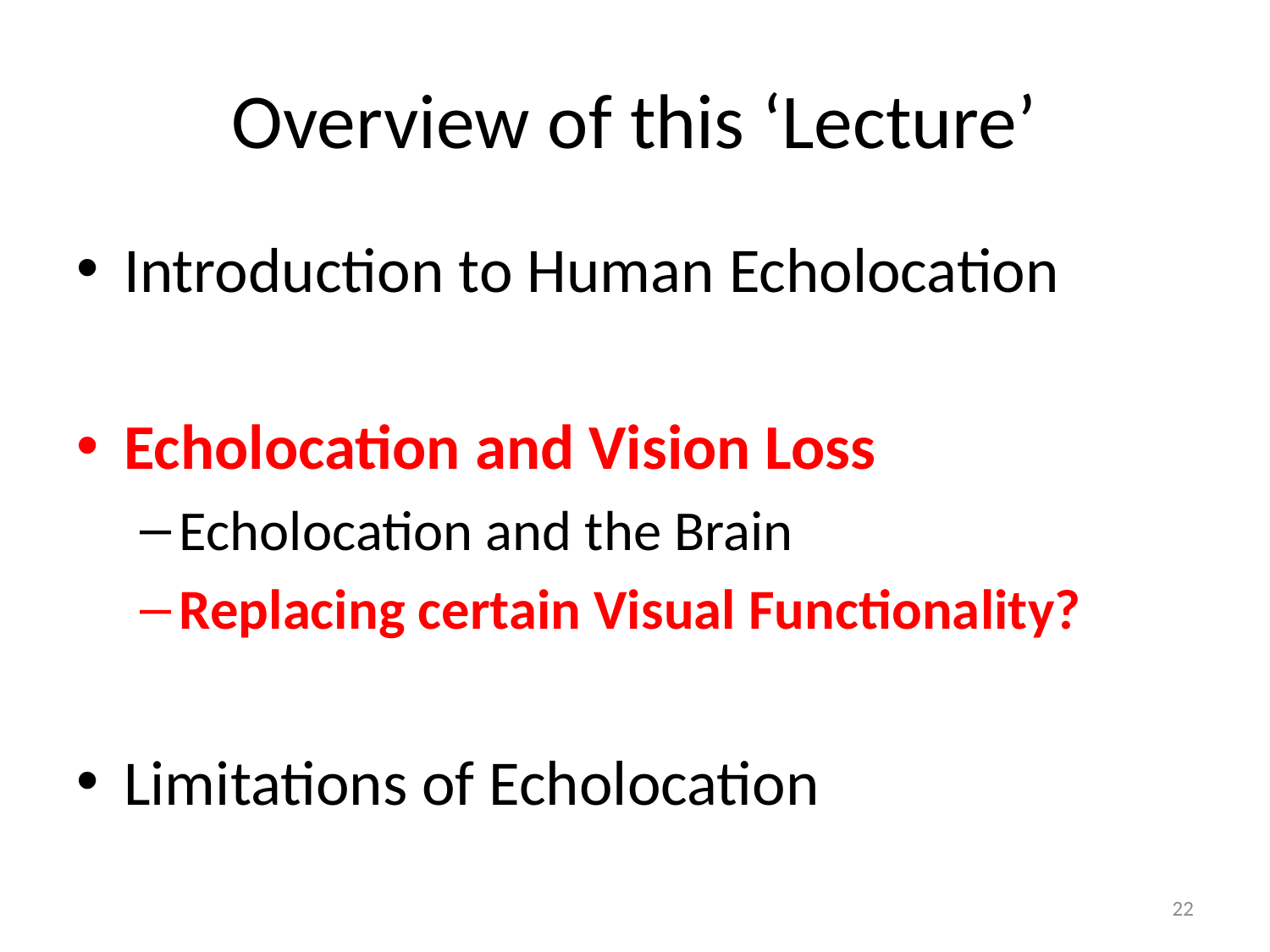

# Overview of this ‘Lecture’
Introduction to Human Echolocation
Echolocation and Vision Loss
Echolocation and the Brain
Replacing certain Visual Functionality?
Limitations of Echolocation
22

## Slide 23
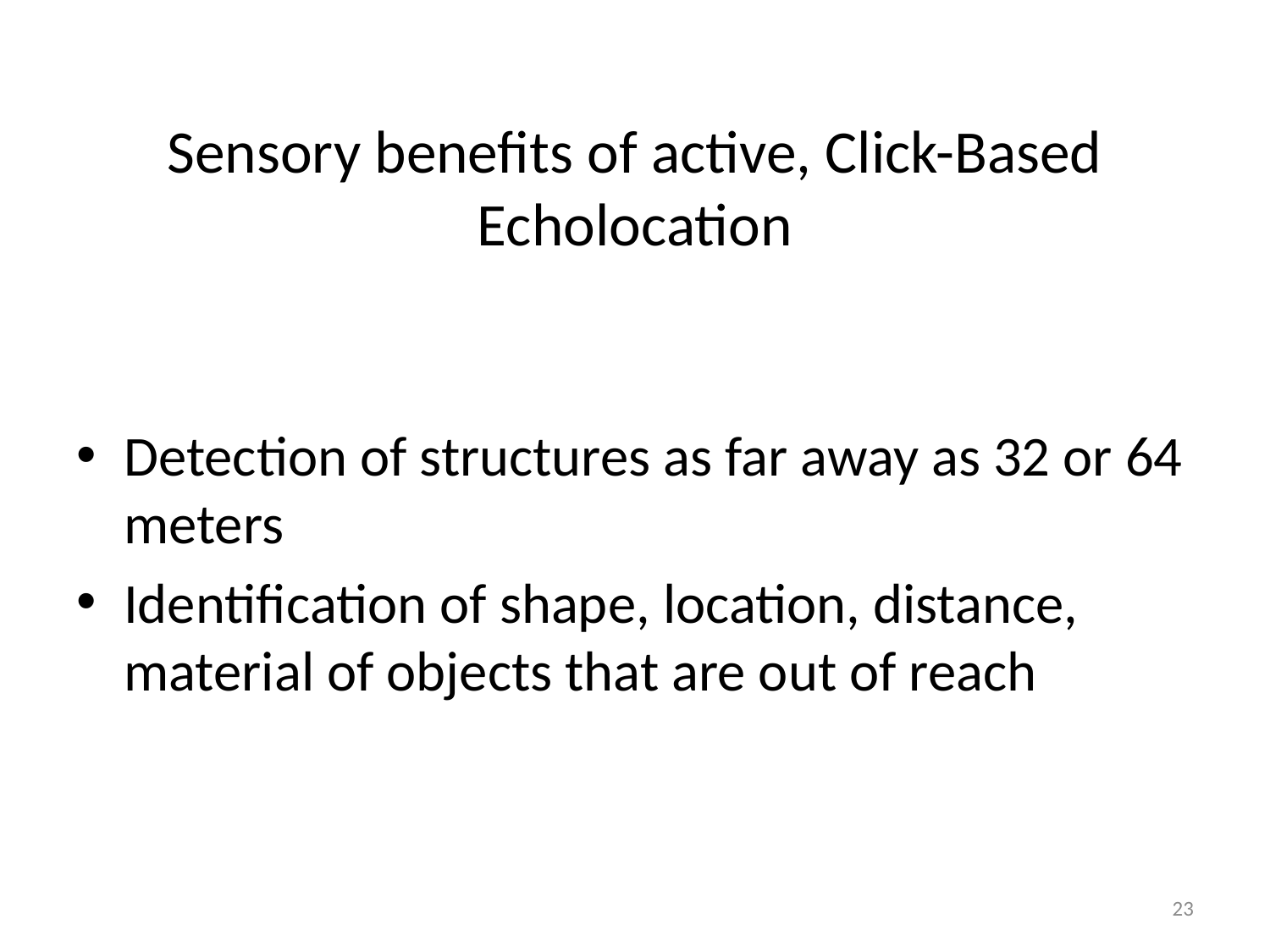

# Sensory benefits of active, Click-Based Echolocation
Detection of structures as far away as 32 or 64 meters
Identification of shape, location, distance, material of objects that are out of reach
23

## Slide 24
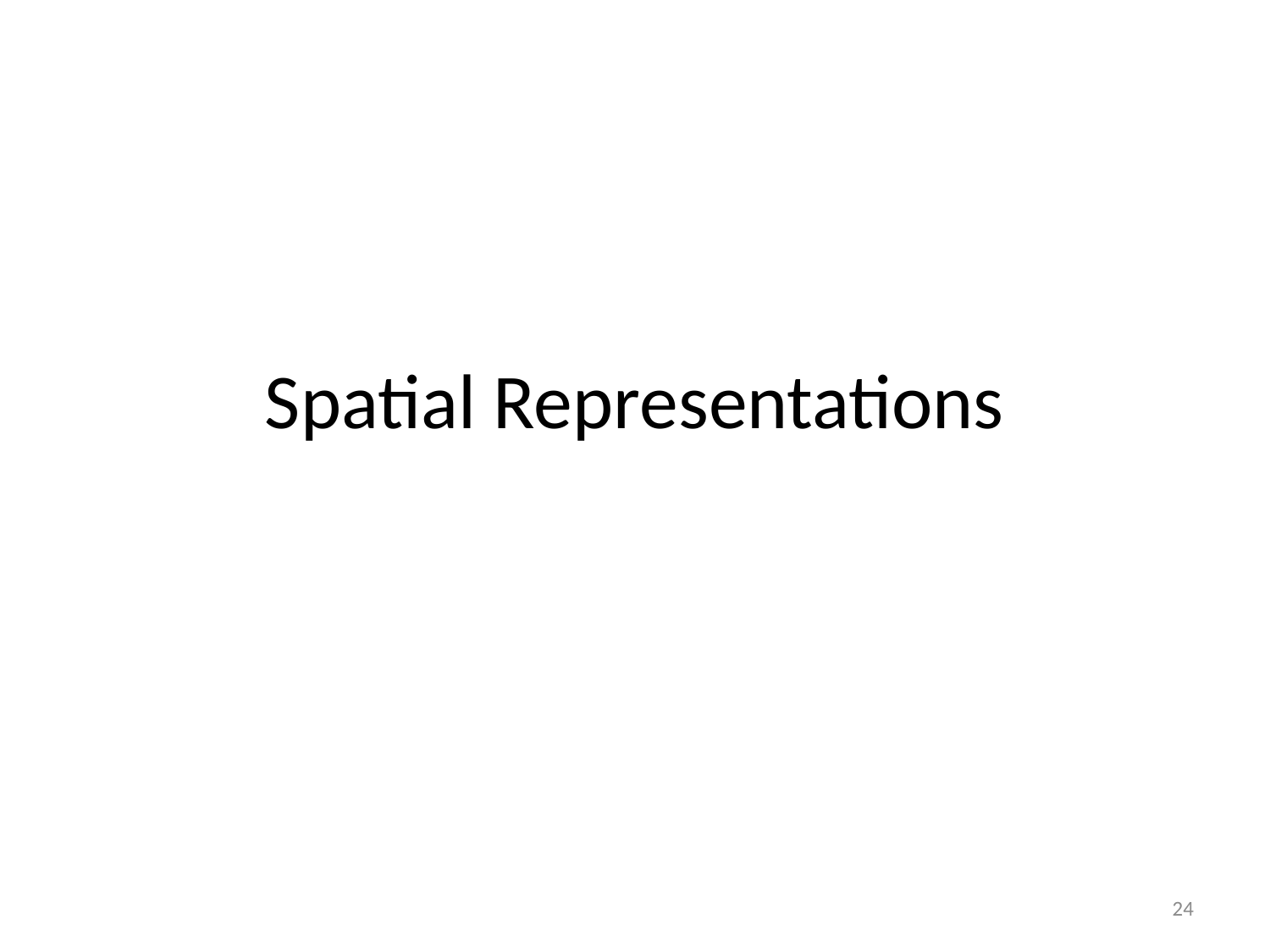

# Spatial Representations
24

## Slide 25
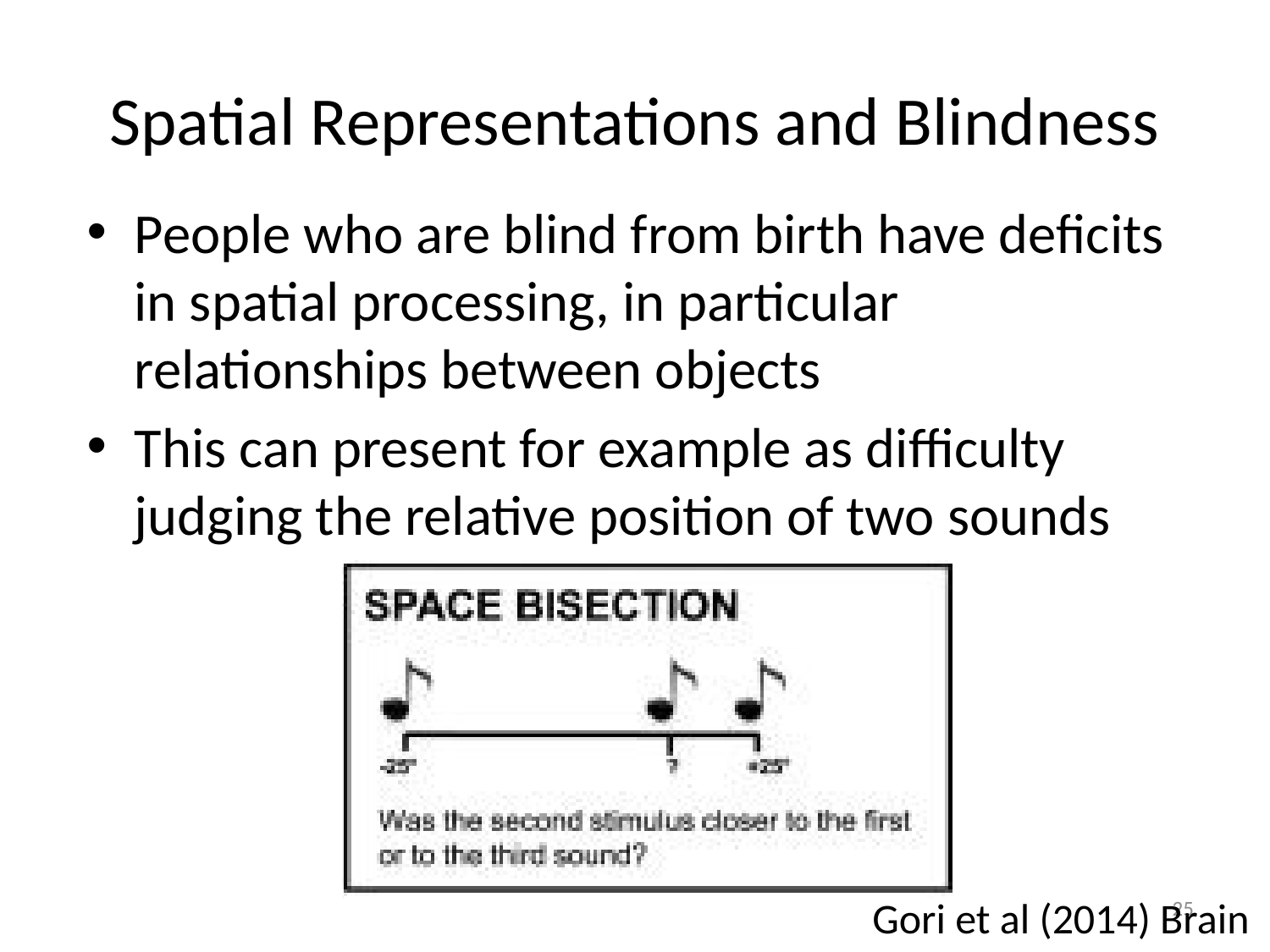

# Spatial Representations and Blindness
People who are blind from birth have deficits in spatial processing, in particular relationships between objects
This can present for example as difficulty judging the relative position of two sounds
25
Gori et al (2014) Brain

## Slide 26
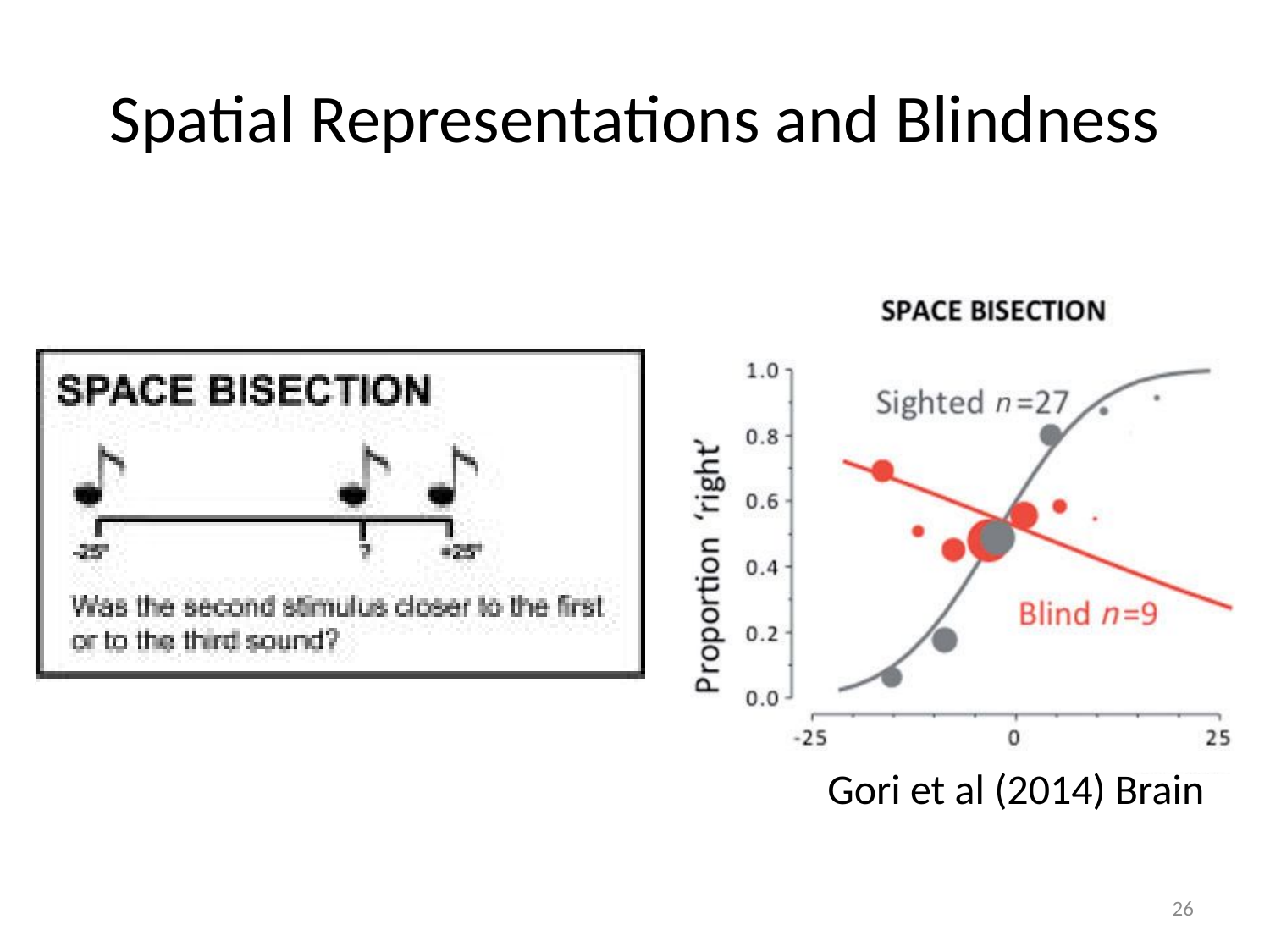

# Spatial Representations and Blindness
Gori et al (2014) Brain
26

## Slide 27
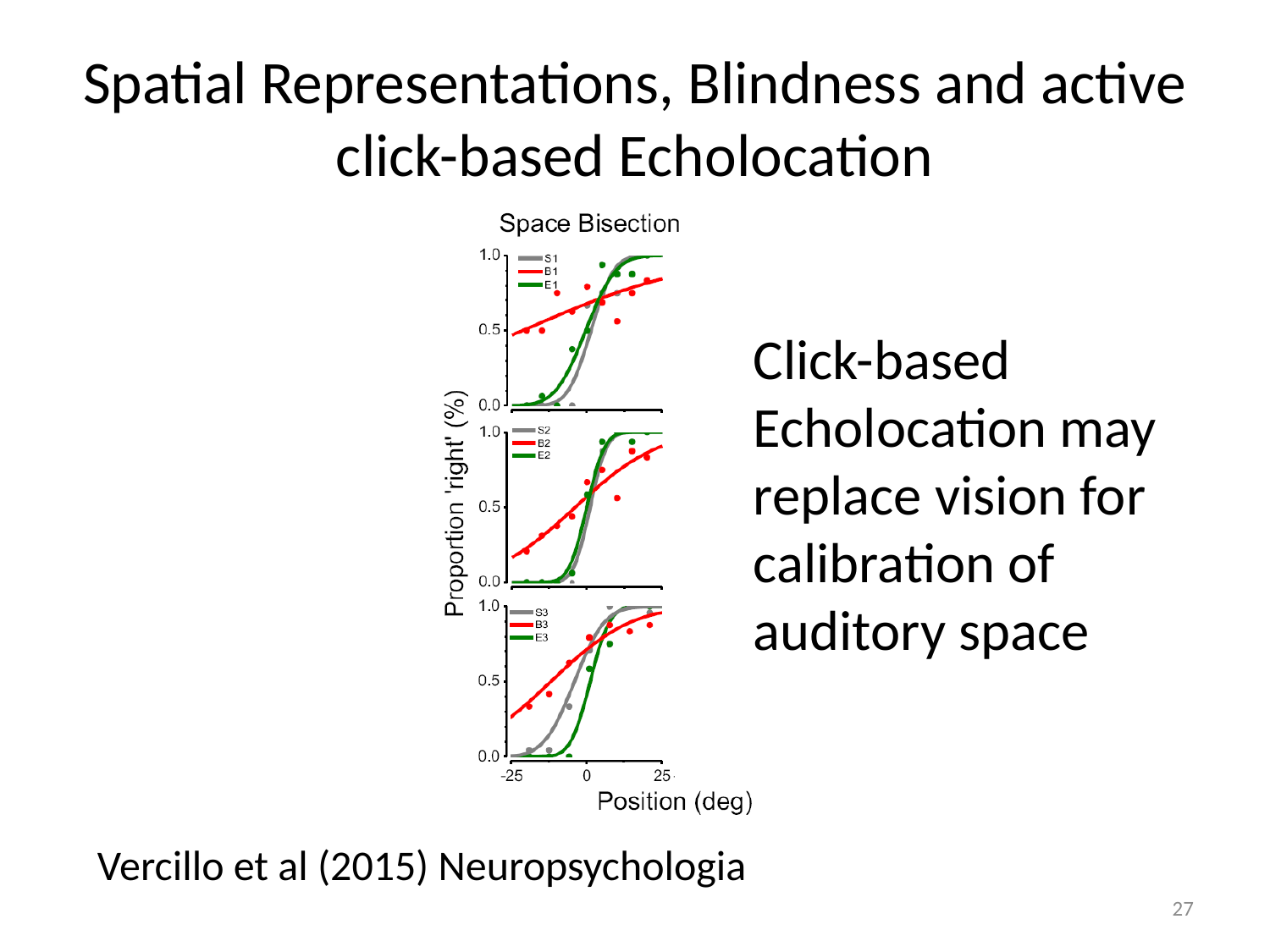

# Spatial Representations, Blindness and active click-based Echolocation
Click-based Echolocation may replace vision for calibration of auditory space
Vercillo et al (2015) Neuropsychologia
27

## Slide 28
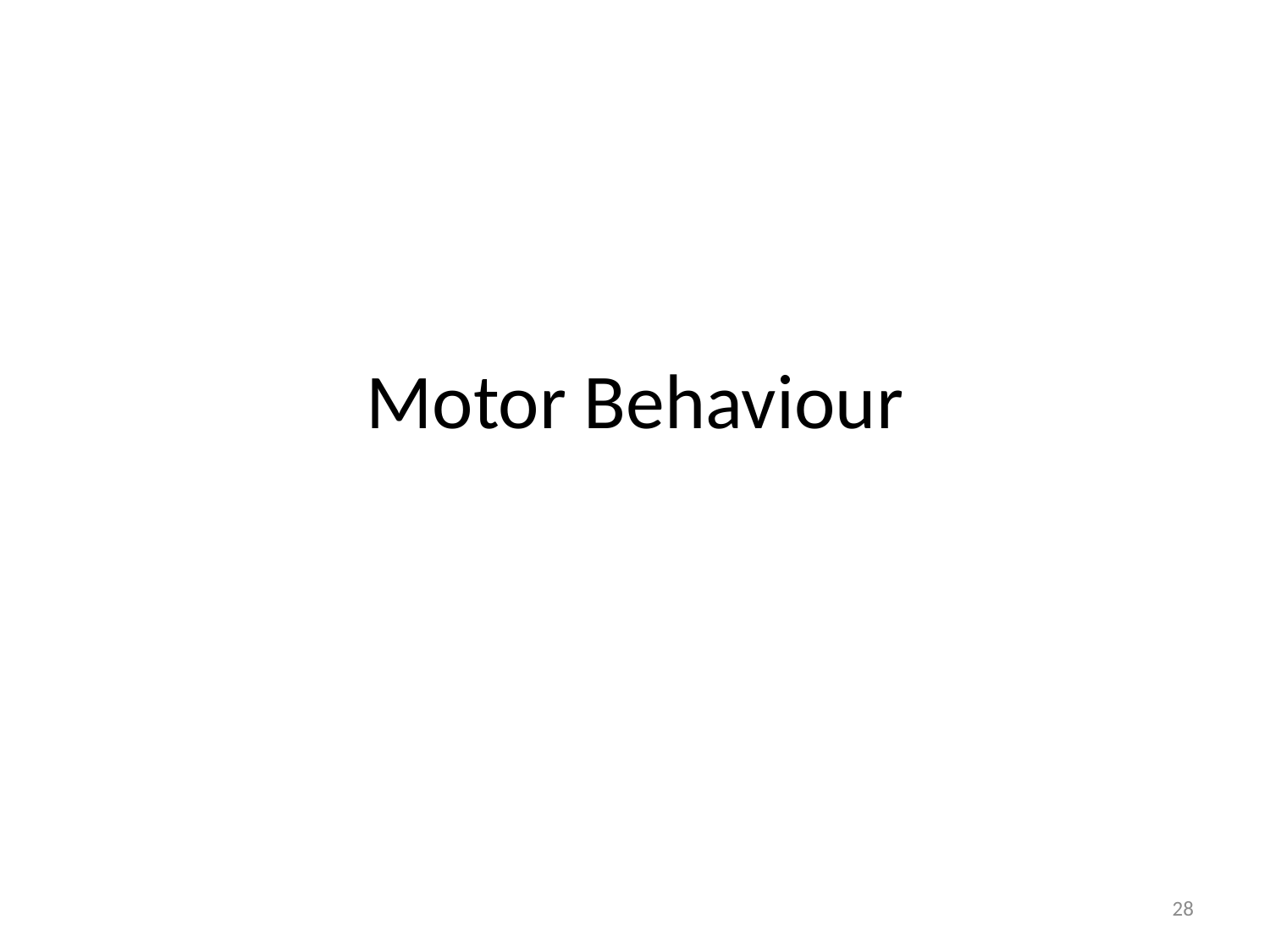

# Motor Behaviour
28

## Slide 29
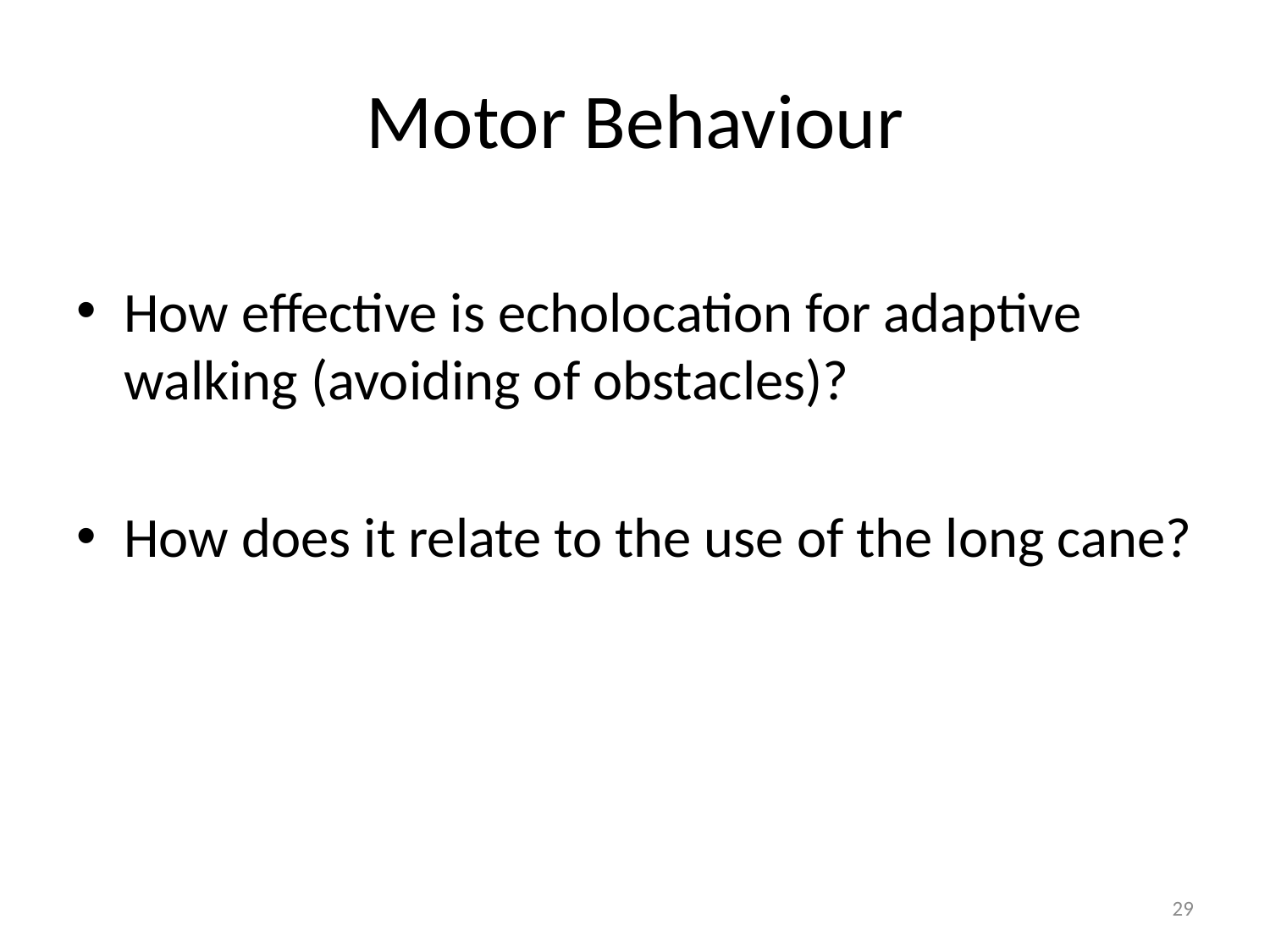

# Motor Behaviour
How effective is echolocation for adaptive walking (avoiding of obstacles)?
How does it relate to the use of the long cane?
29

## Slide 30
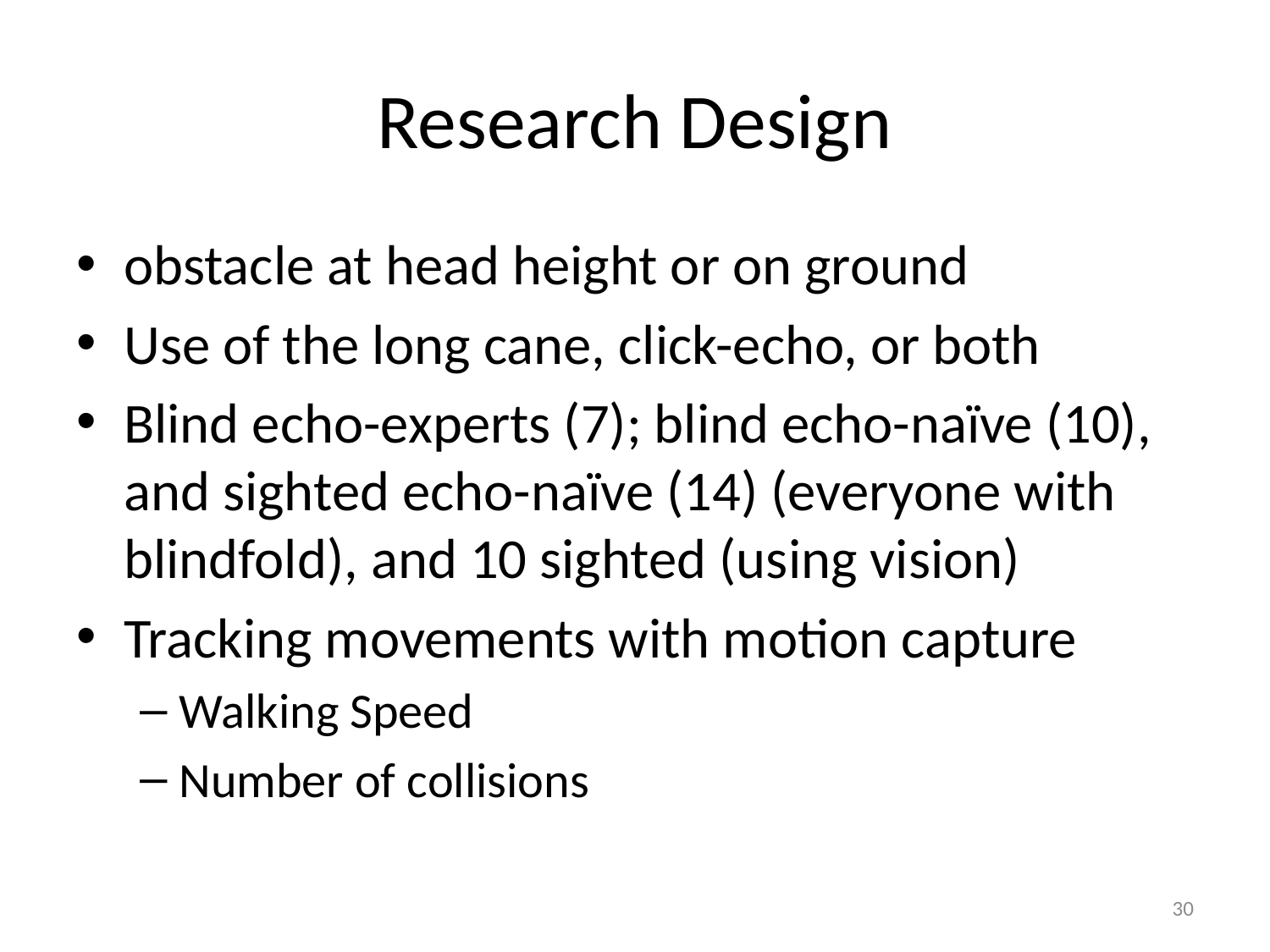

# Research Design
obstacle at head height or on ground
Use of the long cane, click-echo, or both
Blind echo-experts (7); blind echo-naïve (10), and sighted echo-naïve (14) (everyone with blindfold), and 10 sighted (using vision)
Tracking movements with motion capture
Walking Speed
Number of collisions
30

## Slide 31
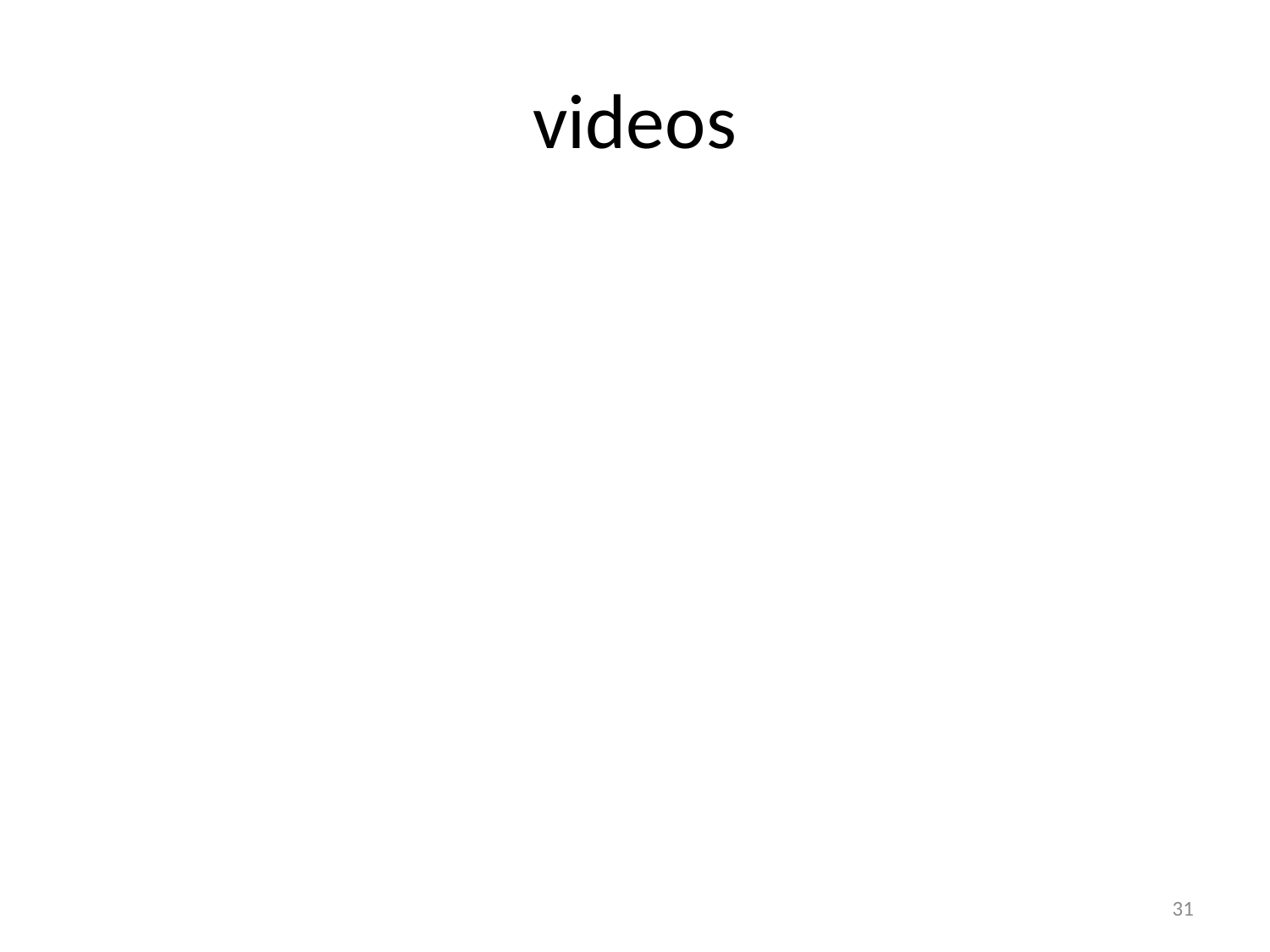

# videos
31

## Slide 32
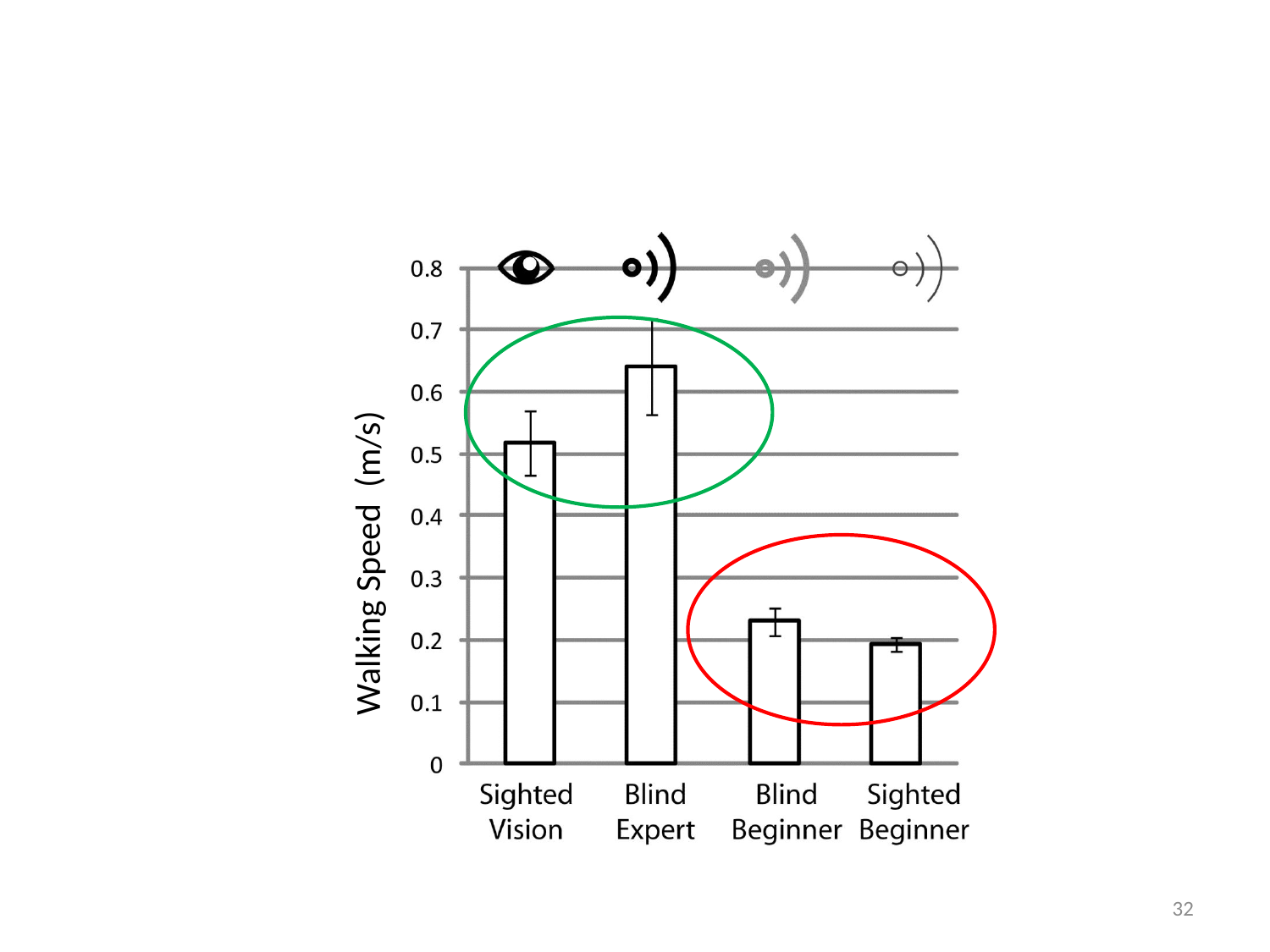

#
Walking Speed (m/s)
32

## Slide 33
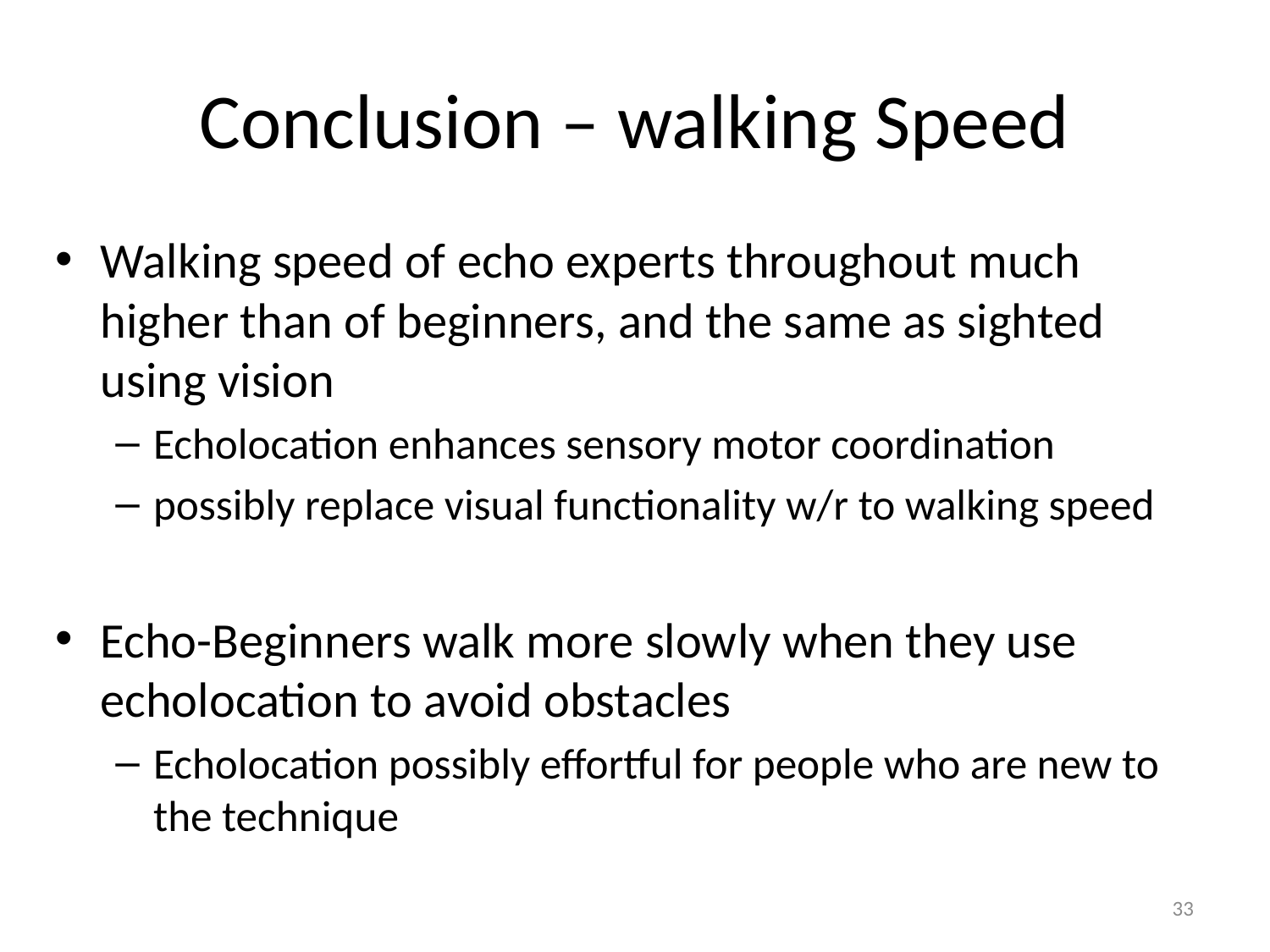

# Conclusion – walking Speed
Walking speed of echo experts throughout much higher than of beginners, and the same as sighted using vision
Echolocation enhances sensory motor coordination
possibly replace visual functionality w/r to walking speed
Echo-Beginners walk more slowly when they use echolocation to avoid obstacles
Echolocation possibly effortful for people who are new to the technique
33

## Slide 34
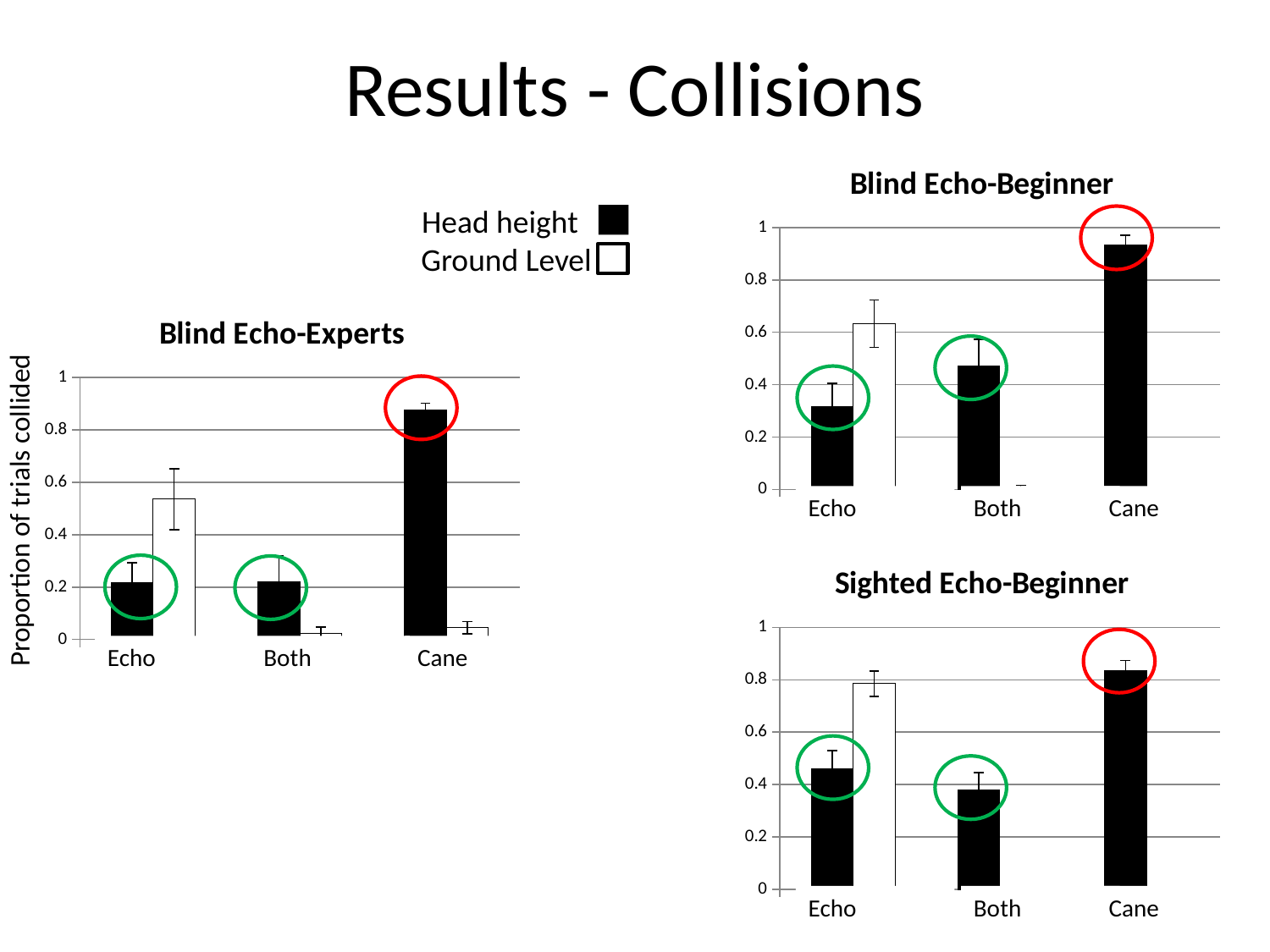

# Results - Collisions
### Chart: Blind Echo-Beginner
| Category | | |
|---|---|---|Head height
Ground Level
### Chart: Blind Echo-Experts
| Category | | |
|---|---|---|
Proportion of trials collided
Echo
Both
Cane
### Chart: Sighted Echo-Beginner
| Category | | |
|---|---|---|
Echo
Both
Cane
34
Echo
Both
Cane

## Slide 35
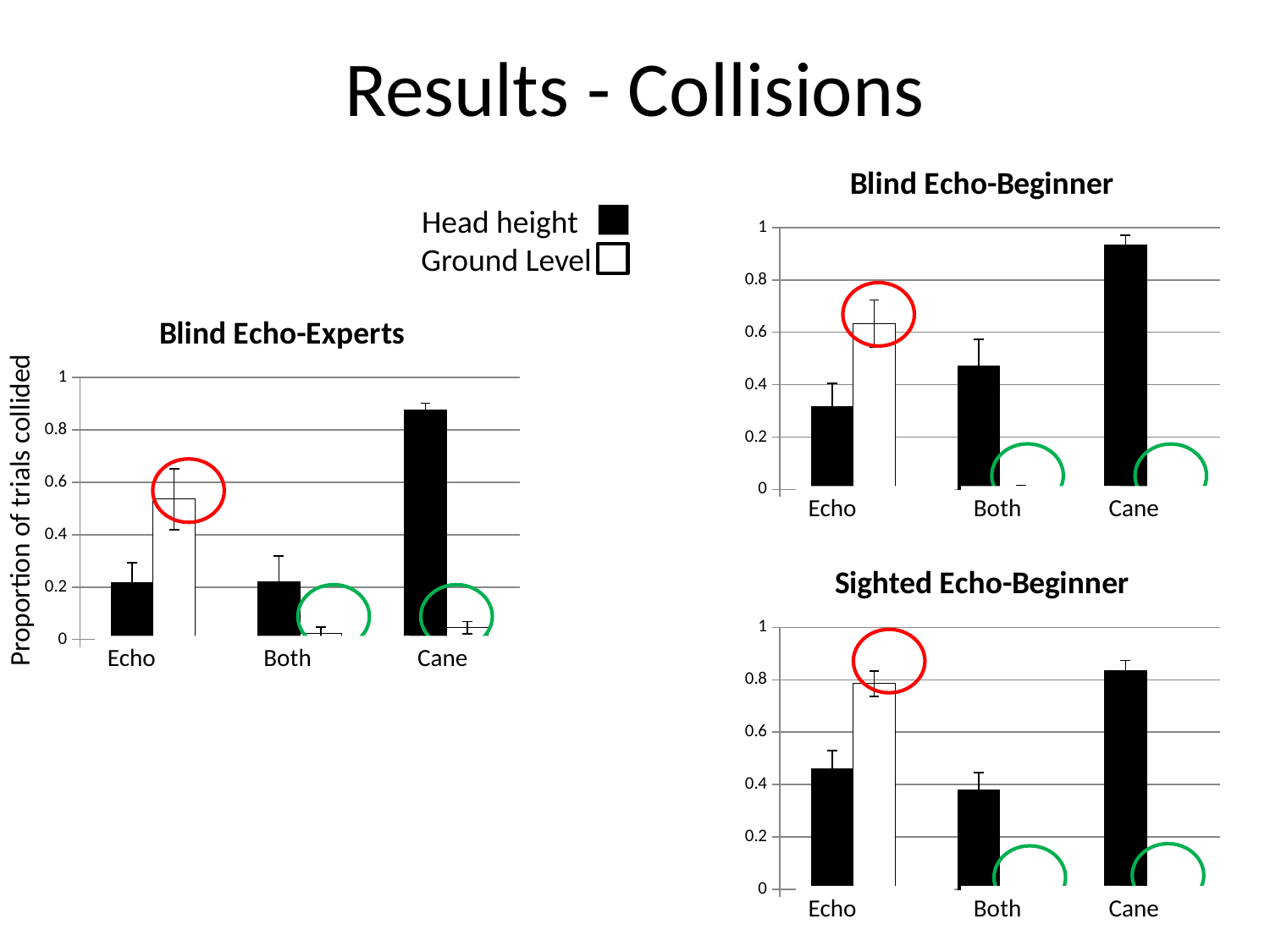

# Results - Collisions
### Chart: Blind Echo-Beginner
| Category | | |
|---|---|---|Head height
Ground Level
### Chart: Blind Echo-Experts
| Category | | |
|---|---|---|
Proportion of trials collided
Echo
Both
Cane
### Chart: Sighted Echo-Beginner
| Category | | |
|---|---|---|
Echo
Both
Cane
35
Echo
Both
Cane

## Slide 36
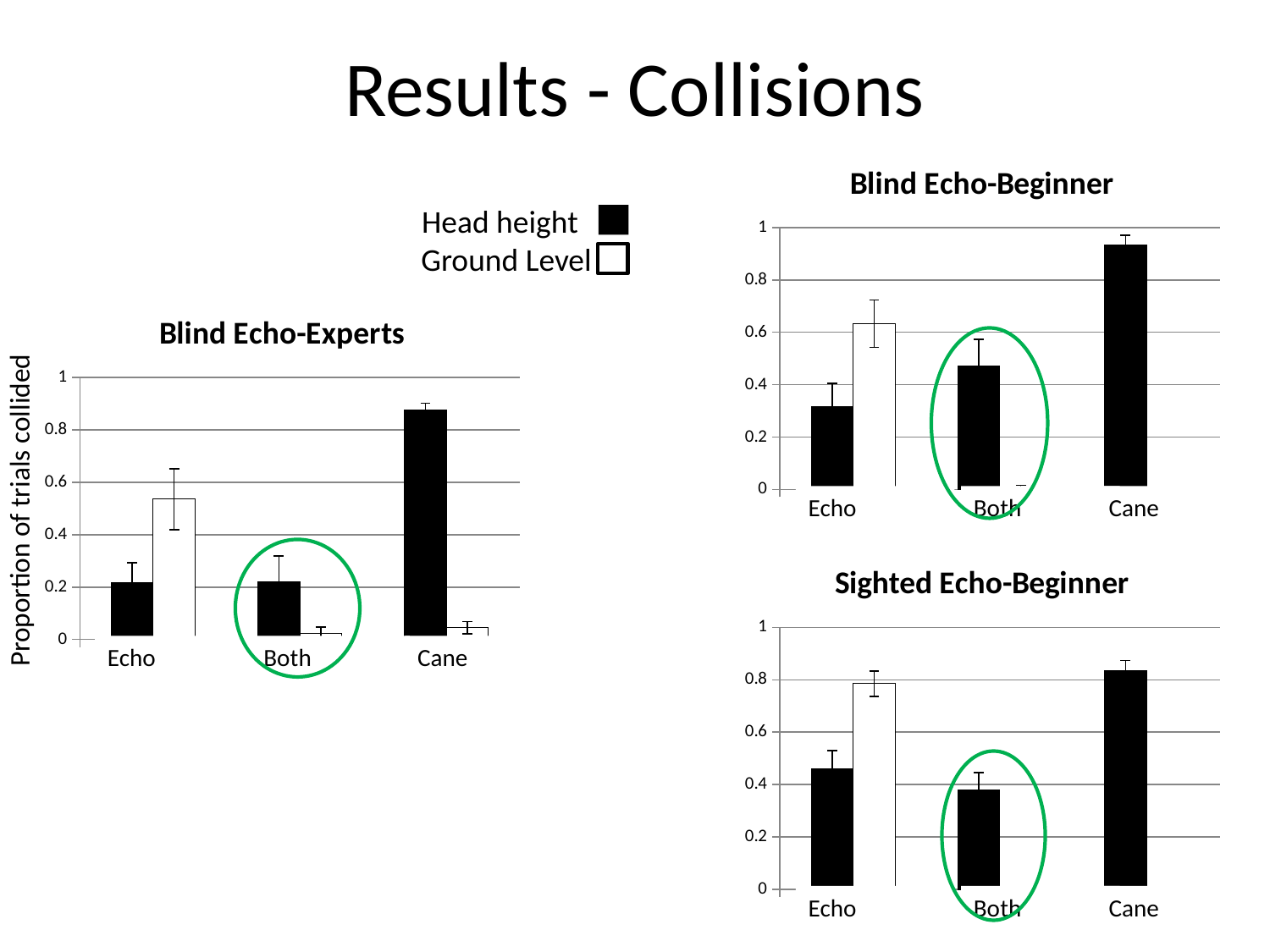

# Results - Collisions
### Chart: Blind Echo-Beginner
| Category | | |
|---|---|---|Head height
Ground Level
### Chart: Blind Echo-Experts
| Category | | |
|---|---|---|
Proportion of trials collided
Echo
Both
Cane
### Chart: Sighted Echo-Beginner
| Category | | |
|---|---|---|Echo
Both
Cane
36
Echo
Both
Cane

## Slide 37
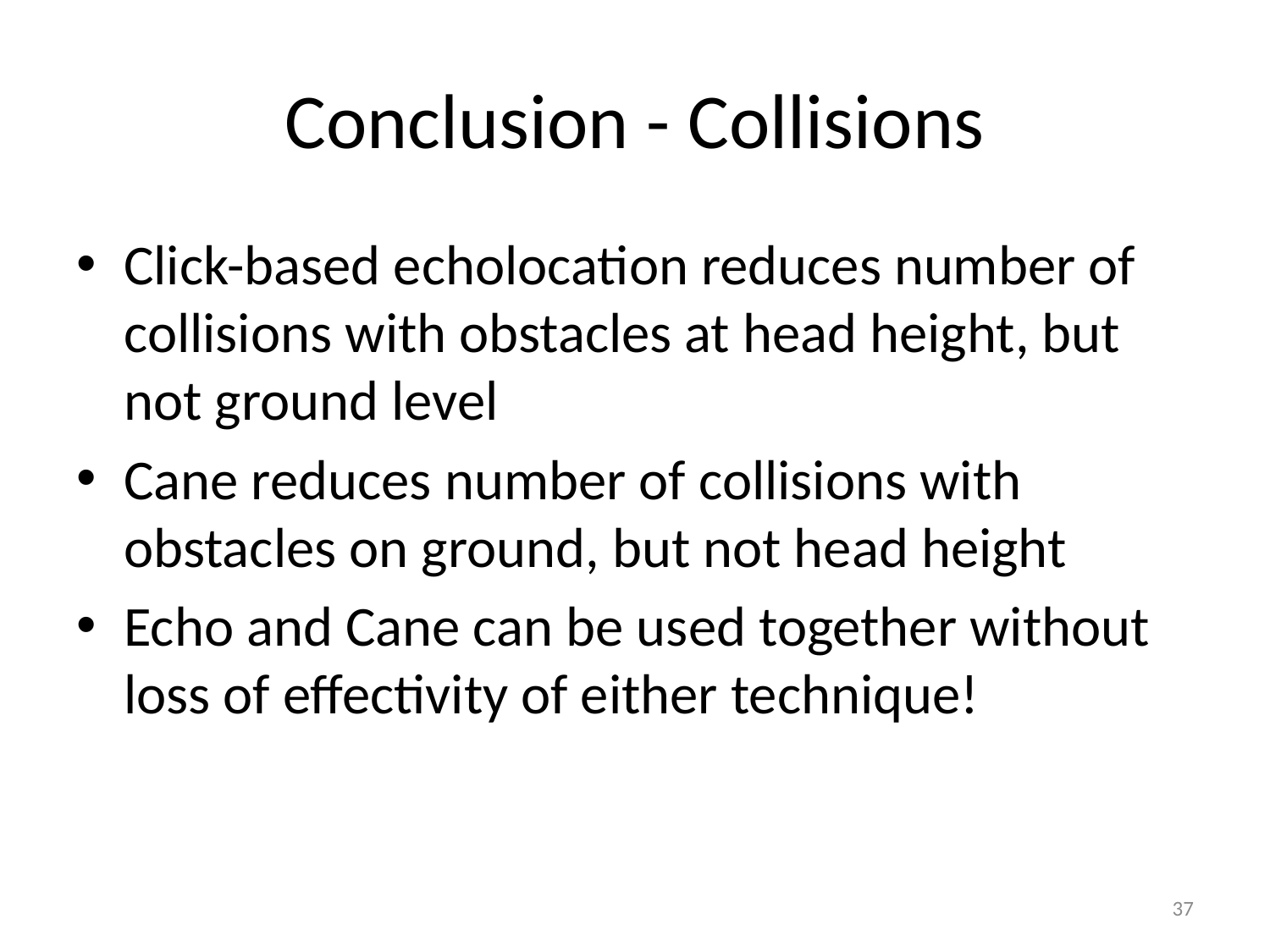

# Conclusion - Collisions
Click-based echolocation reduces number of collisions with obstacles at head height, but not ground level
Cane reduces number of collisions with obstacles on ground, but not head height
Echo and Cane can be used together without loss of effectivity of either technique!
37

## Slide 38
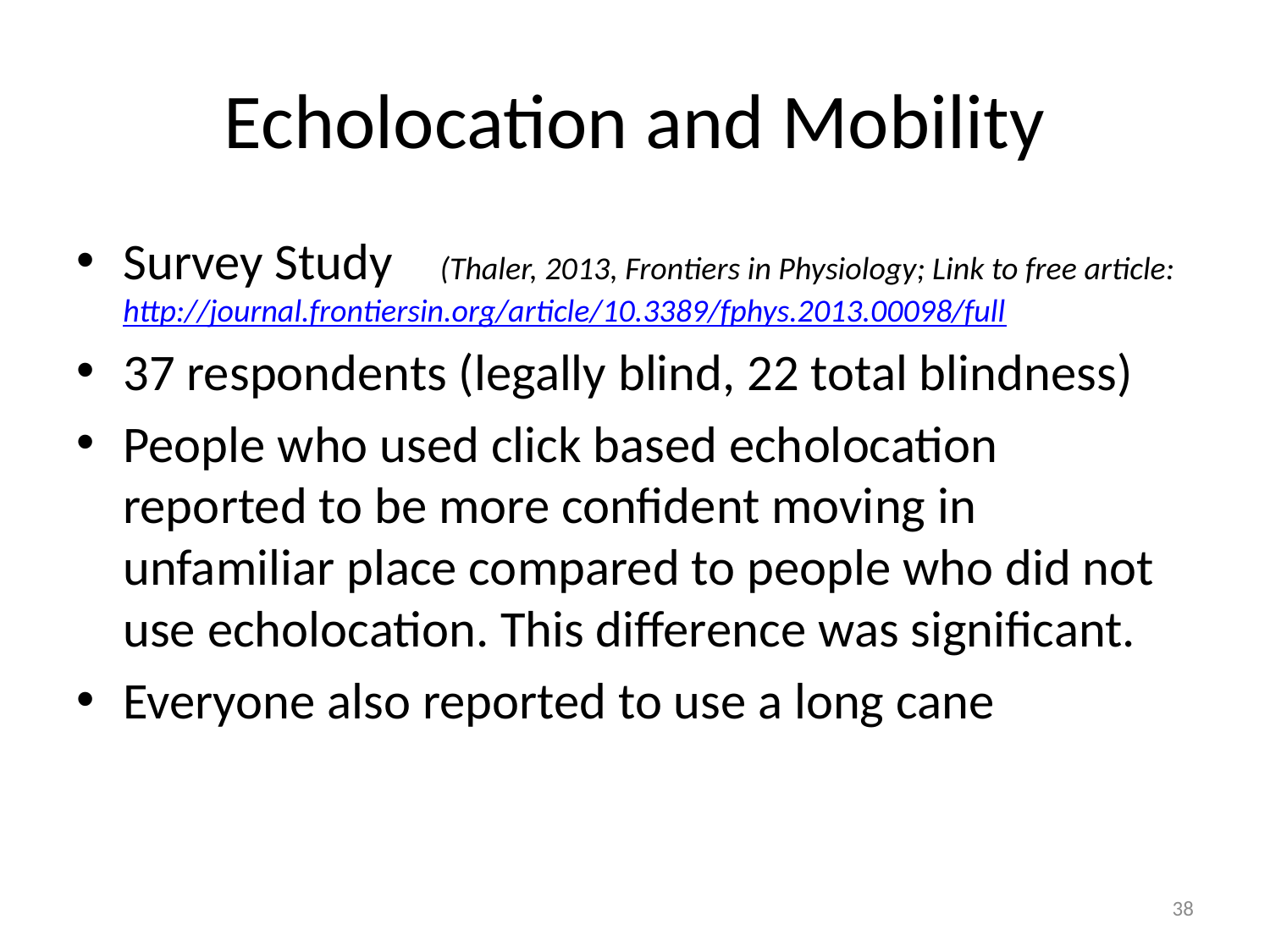

# Echolocation and Mobility
Survey Study (Thaler, 2013, Frontiers in Physiology; Link to free article: http://journal.frontiersin.org/article/10.3389/fphys.2013.00098/full
37 respondents (legally blind, 22 total blindness)
People who used click based echolocation reported to be more confident moving in unfamiliar place compared to people who did not use echolocation. This difference was significant.
Everyone also reported to use a long cane
38

## Slide 39
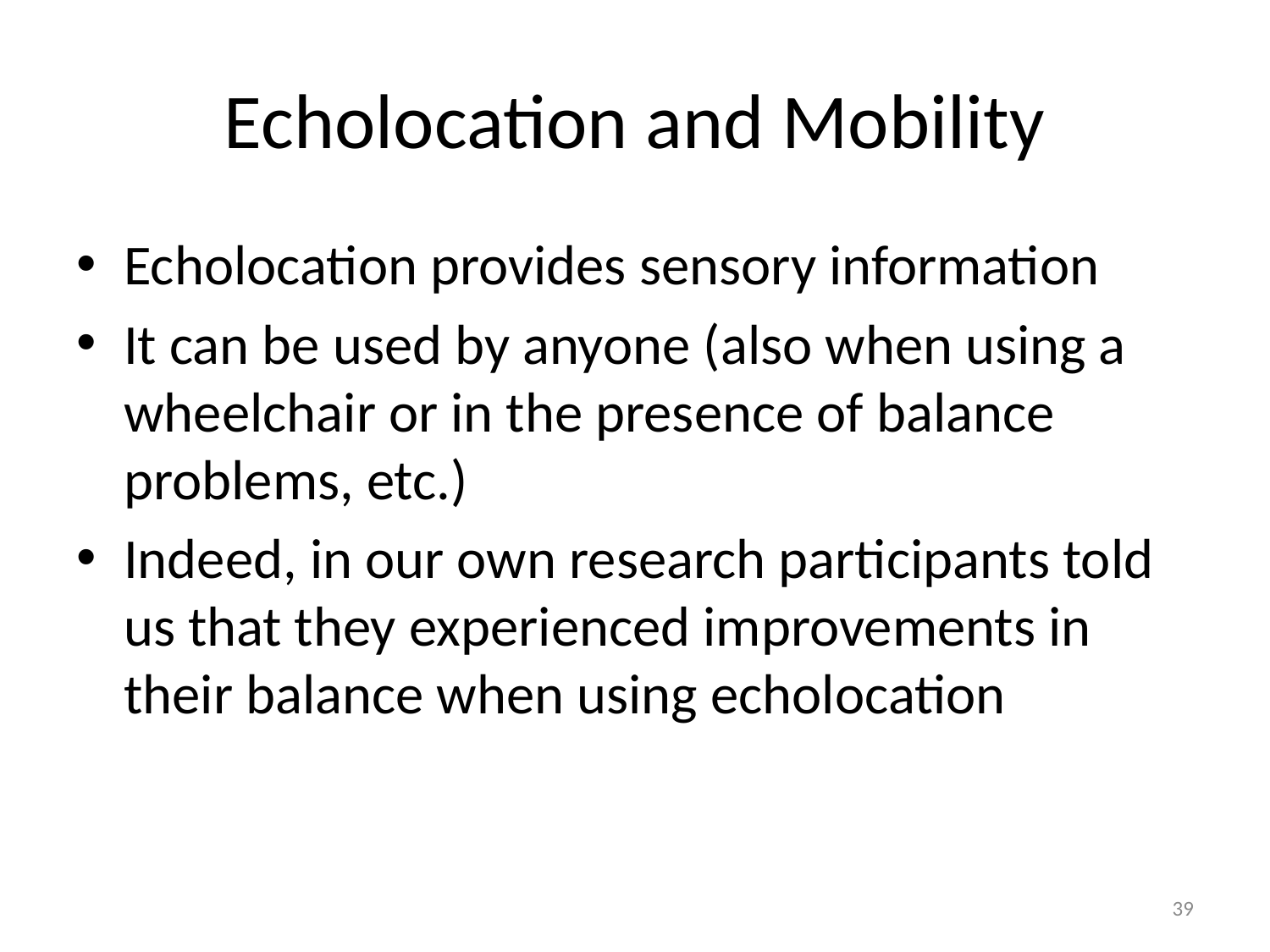

# Echolocation and Mobility
Echolocation provides sensory information
It can be used by anyone (also when using a wheelchair or in the presence of balance problems, etc.)
Indeed, in our own research participants told us that they experienced improvements in their balance when using echolocation
39

## Slide 40
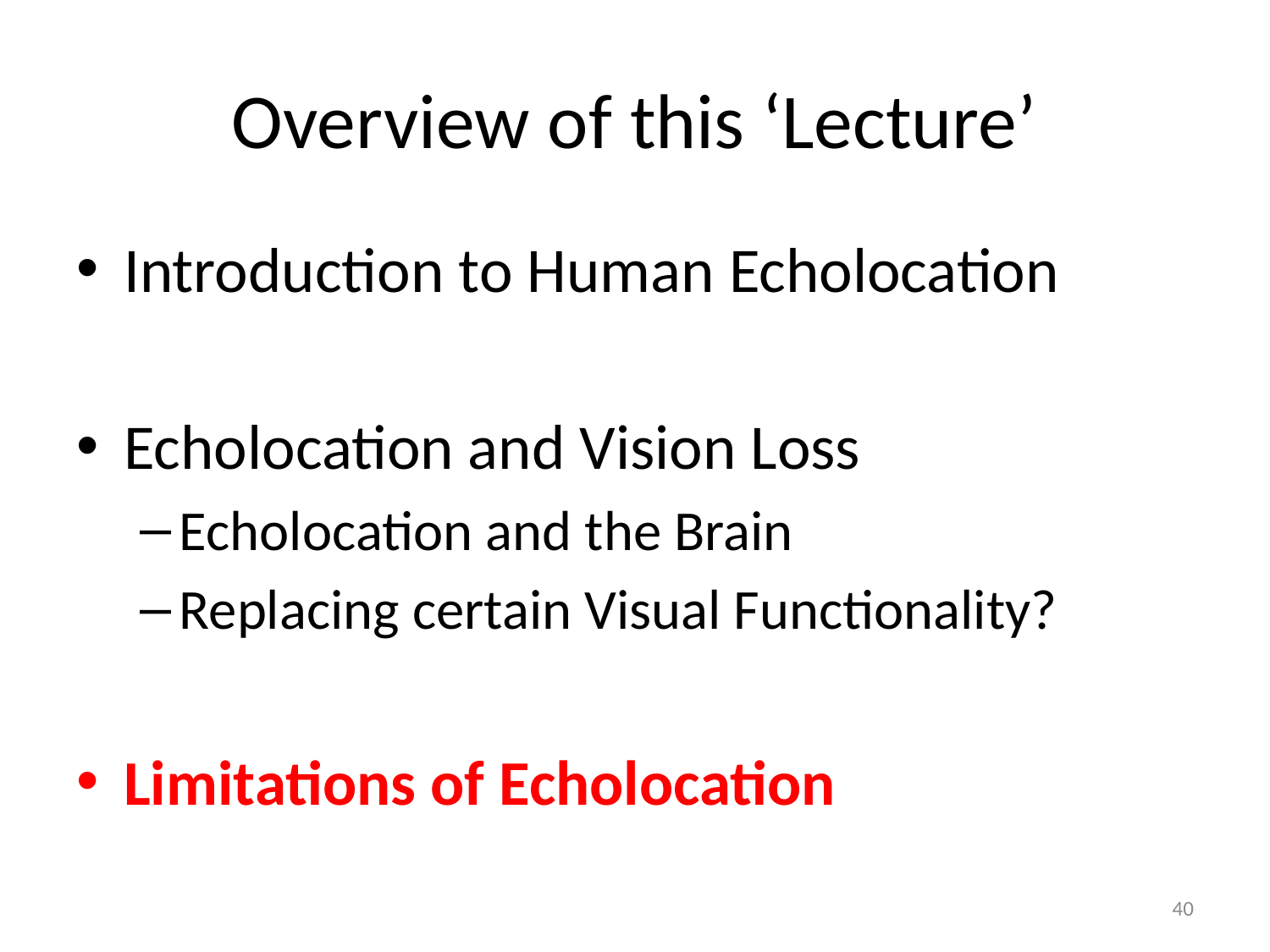

# Overview of this ‘Lecture’
Introduction to Human Echolocation
Echolocation and Vision Loss
Echolocation and the Brain
Replacing certain Visual Functionality?
Limitations of Echolocation
40

## Slide 41
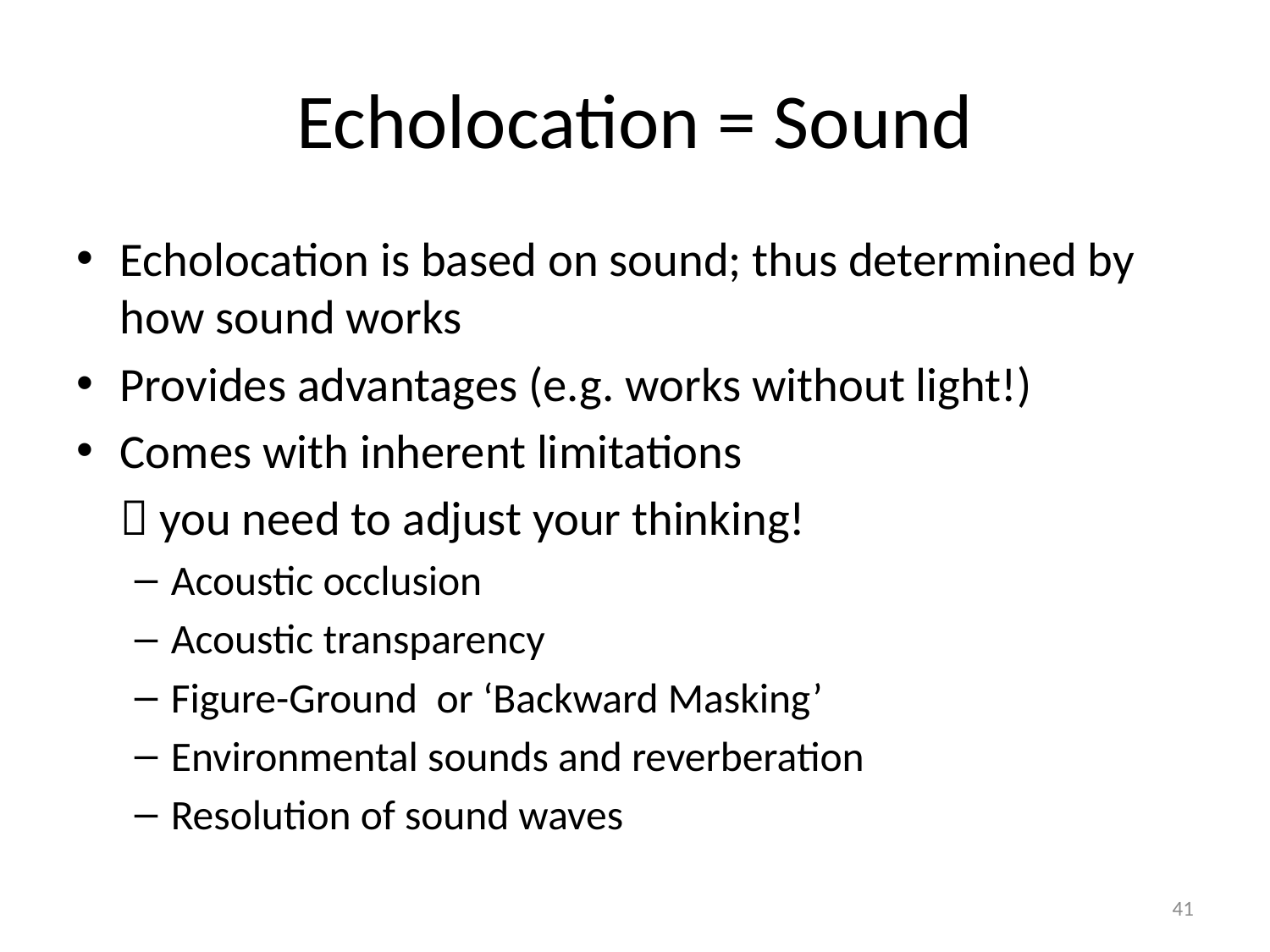

# Echolocation = Sound
Echolocation is based on sound; thus determined by how sound works
Provides advantages (e.g. works without light!)
Comes with inherent limitations
	 you need to adjust your thinking!
Acoustic occlusion
Acoustic transparency
Figure-Ground or ‘Backward Masking’
Environmental sounds and reverberation
Resolution of sound waves
41

## Slide 42
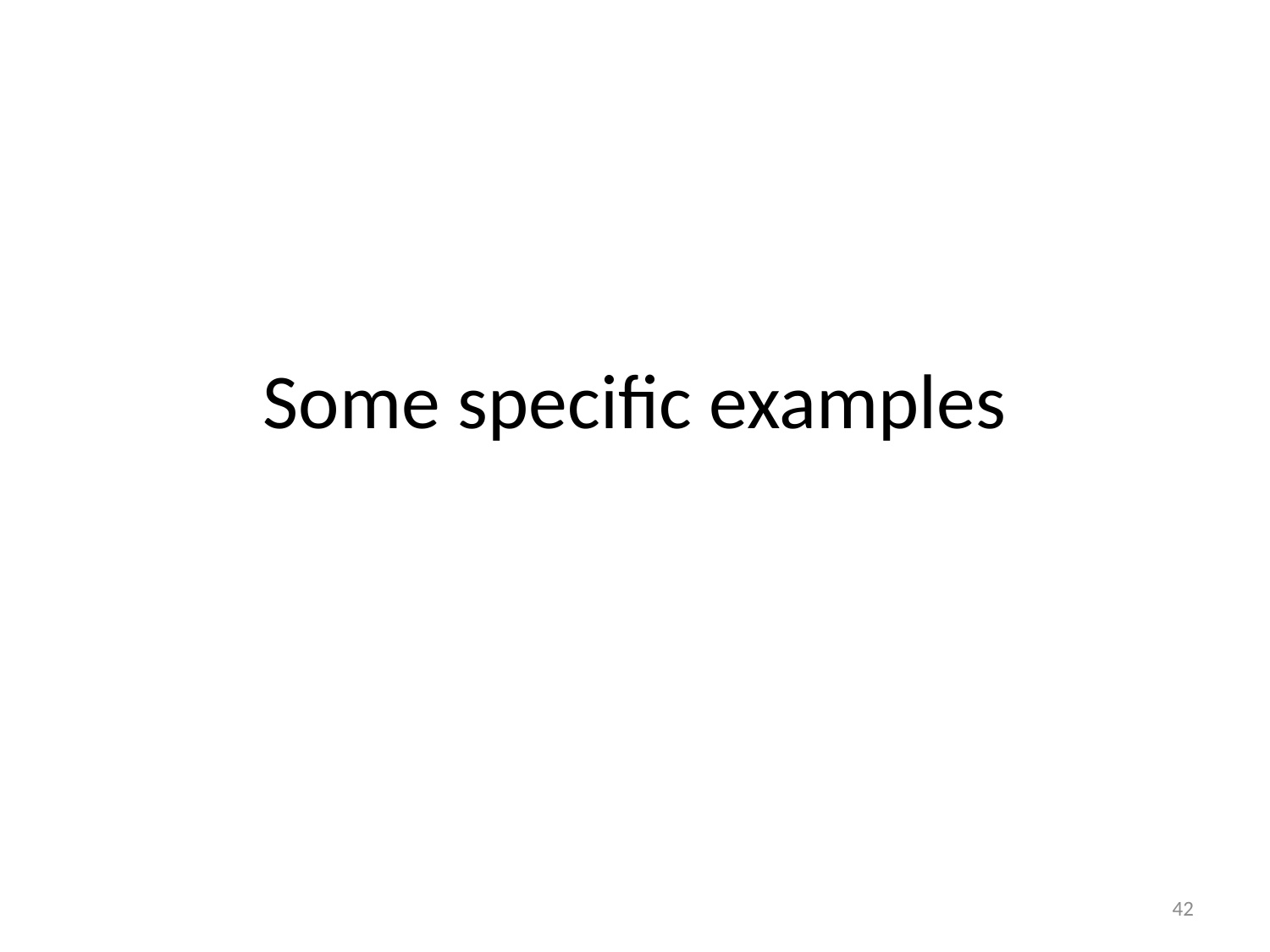

# Some specific examples
42

## Slide 43
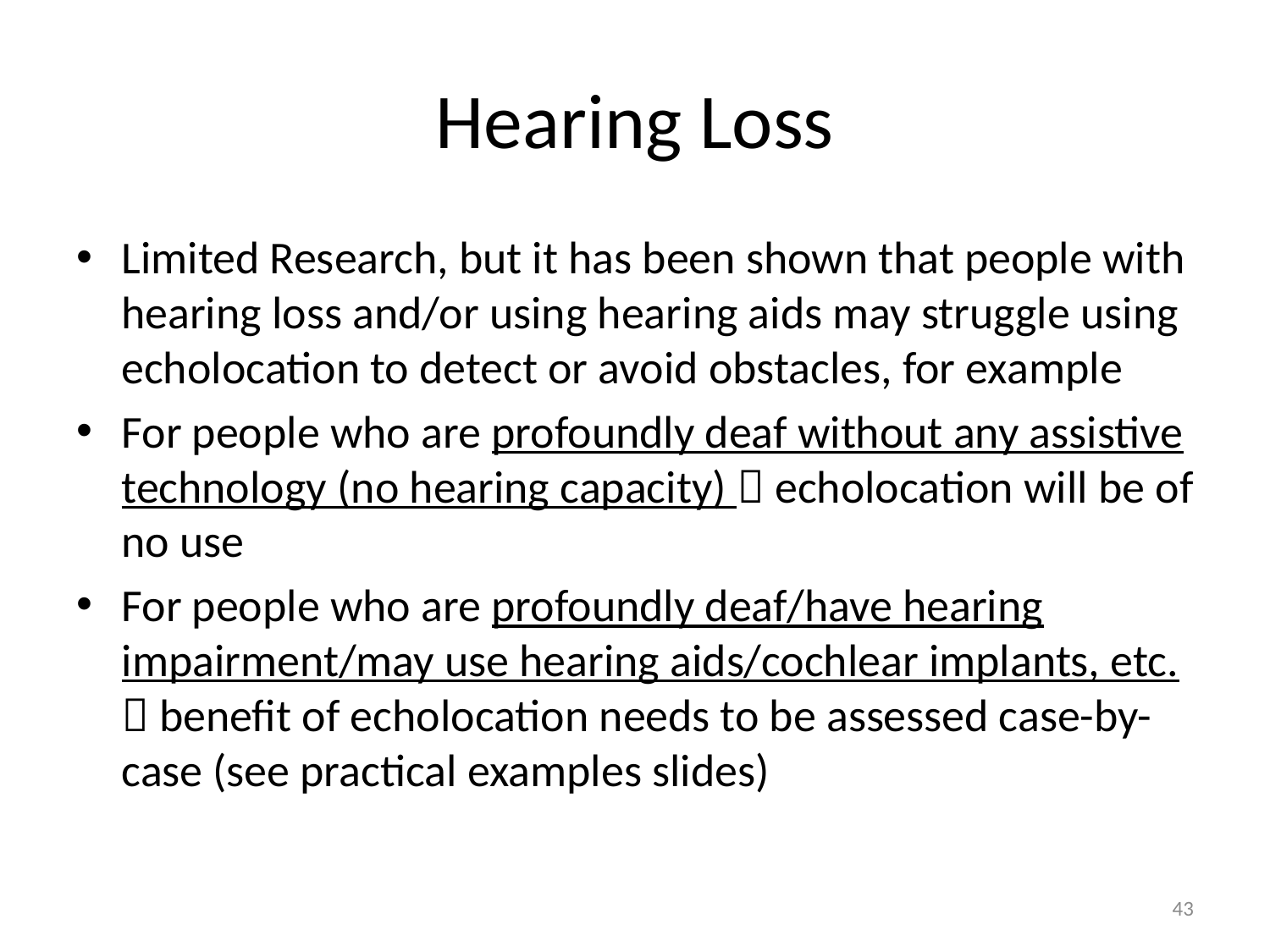

# Hearing Loss
Limited Research, but it has been shown that people with hearing loss and/or using hearing aids may struggle using echolocation to detect or avoid obstacles, for example
For people who are profoundly deaf without any assistive technology (no hearing capacity)  echolocation will be of no use
For people who are profoundly deaf/have hearing impairment/may use hearing aids/cochlear implants, etc.  benefit of echolocation needs to be assessed case-by-case (see practical examples slides)
43

## Slide 44
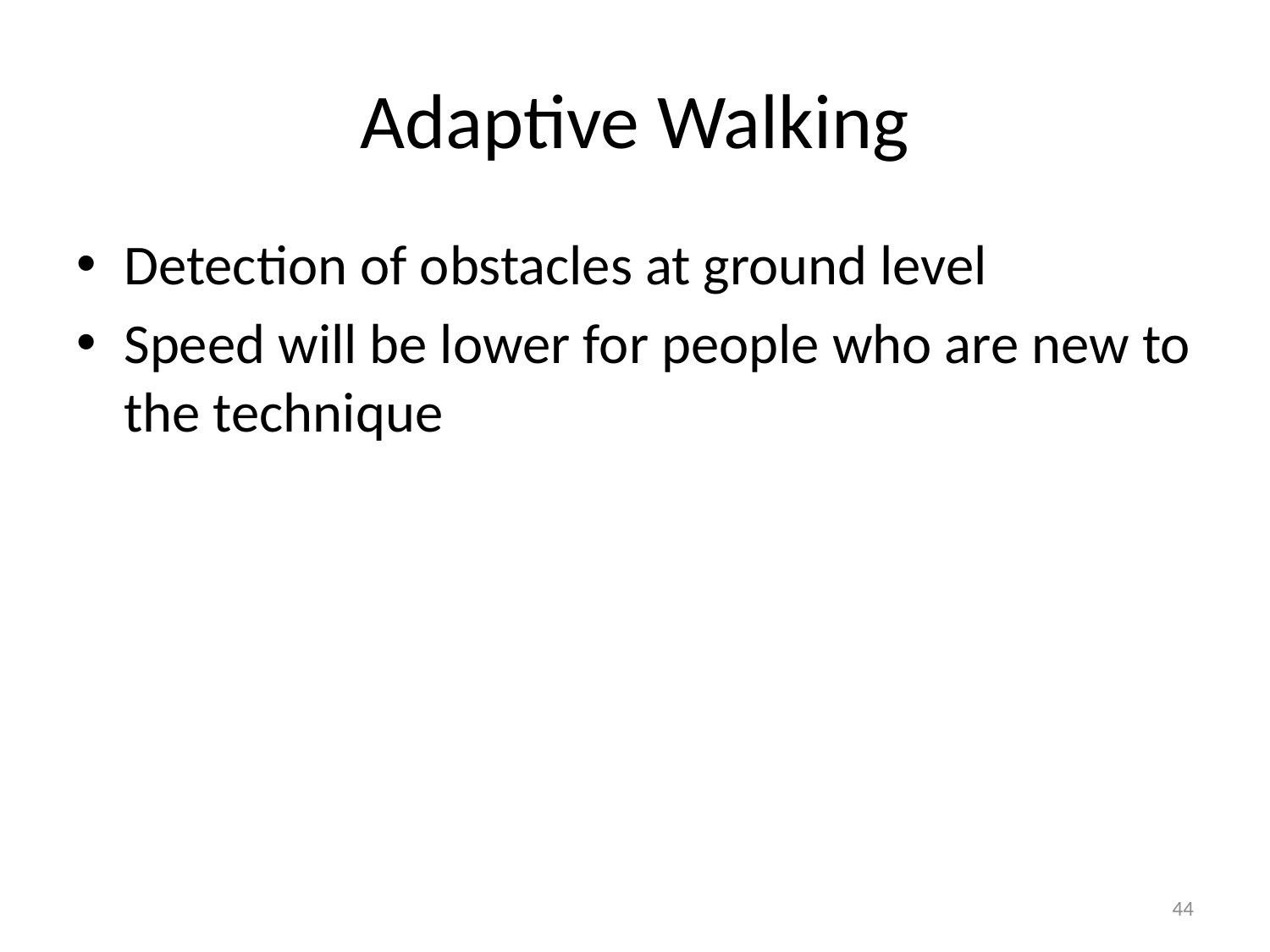

# Adaptive Walking
Detection of obstacles at ground level
Speed will be lower for people who are new to the technique
44

## Slide 45
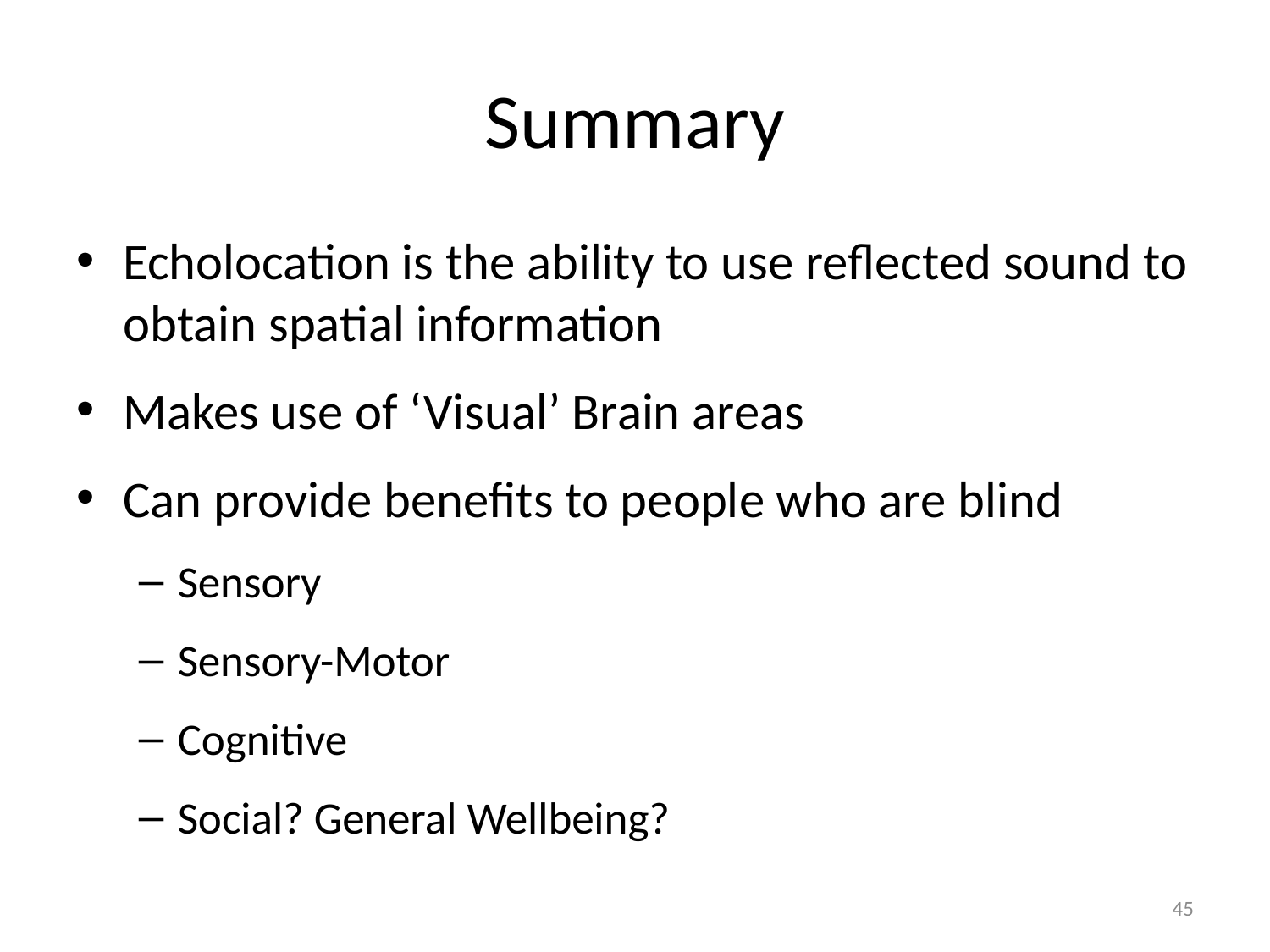

# Summary
Echolocation is the ability to use reflected sound to obtain spatial information
Makes use of ‘Visual’ Brain areas
Can provide benefits to people who are blind
Sensory
Sensory-Motor
Cognitive
Social? General Wellbeing?
45

## Slide 46
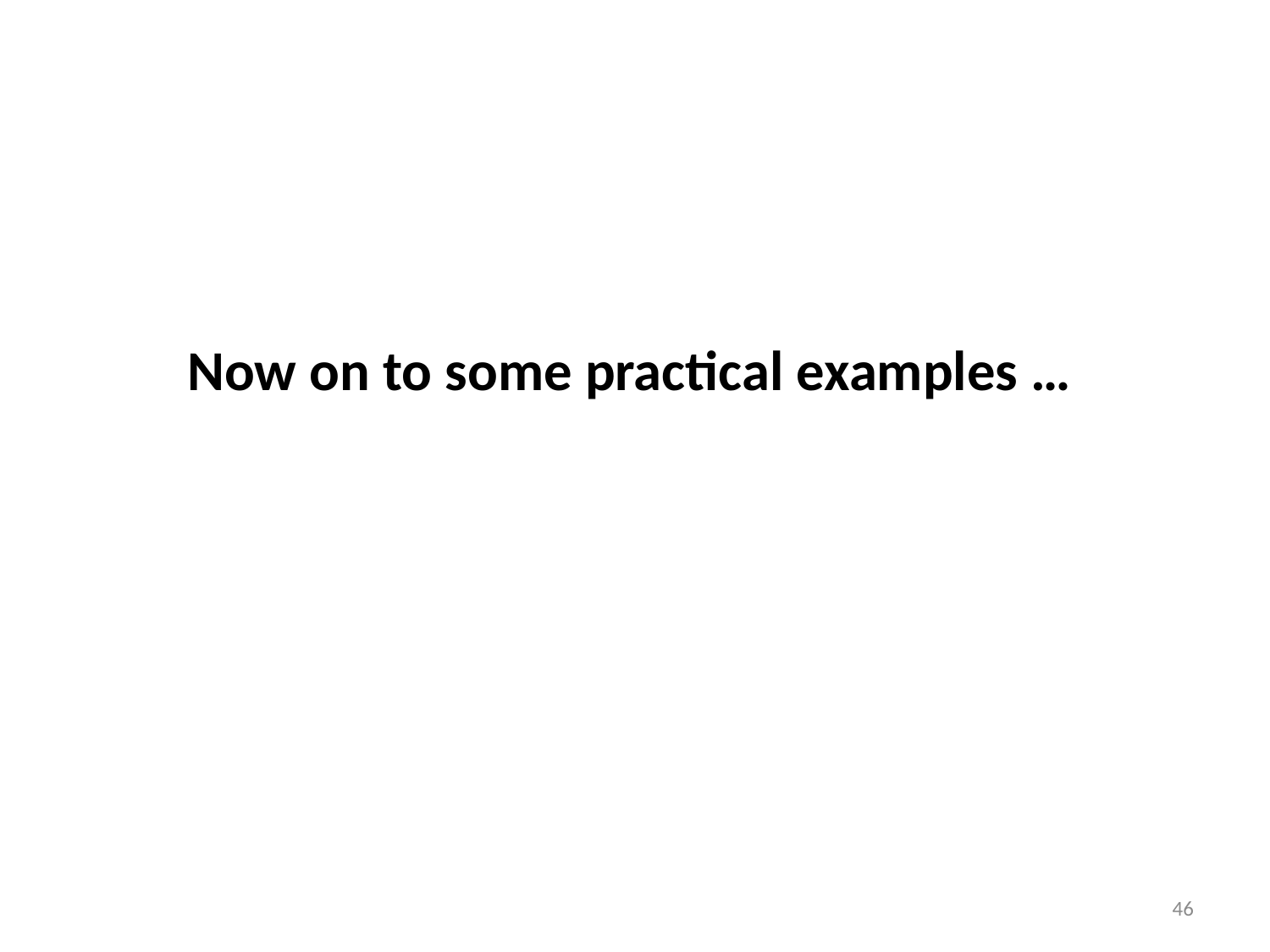

#
Now on to some practical examples …
46

## Slide 47
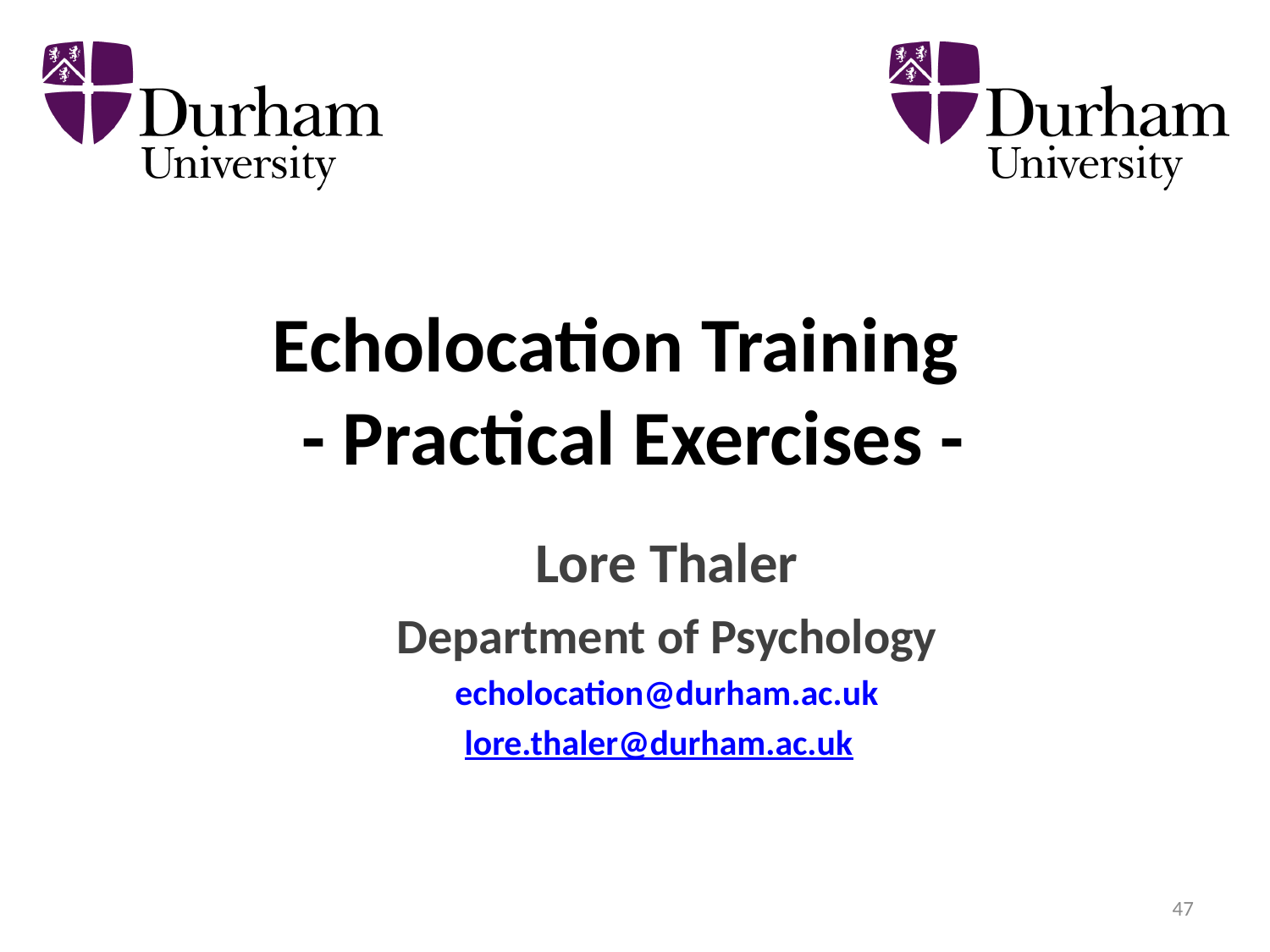

# Echolocation Training - Practical Exercises -
Lore Thaler
Department of Psychology
echolocation@durham.ac.uk
lore.thaler@durham.ac.uk
47

## Slide 48
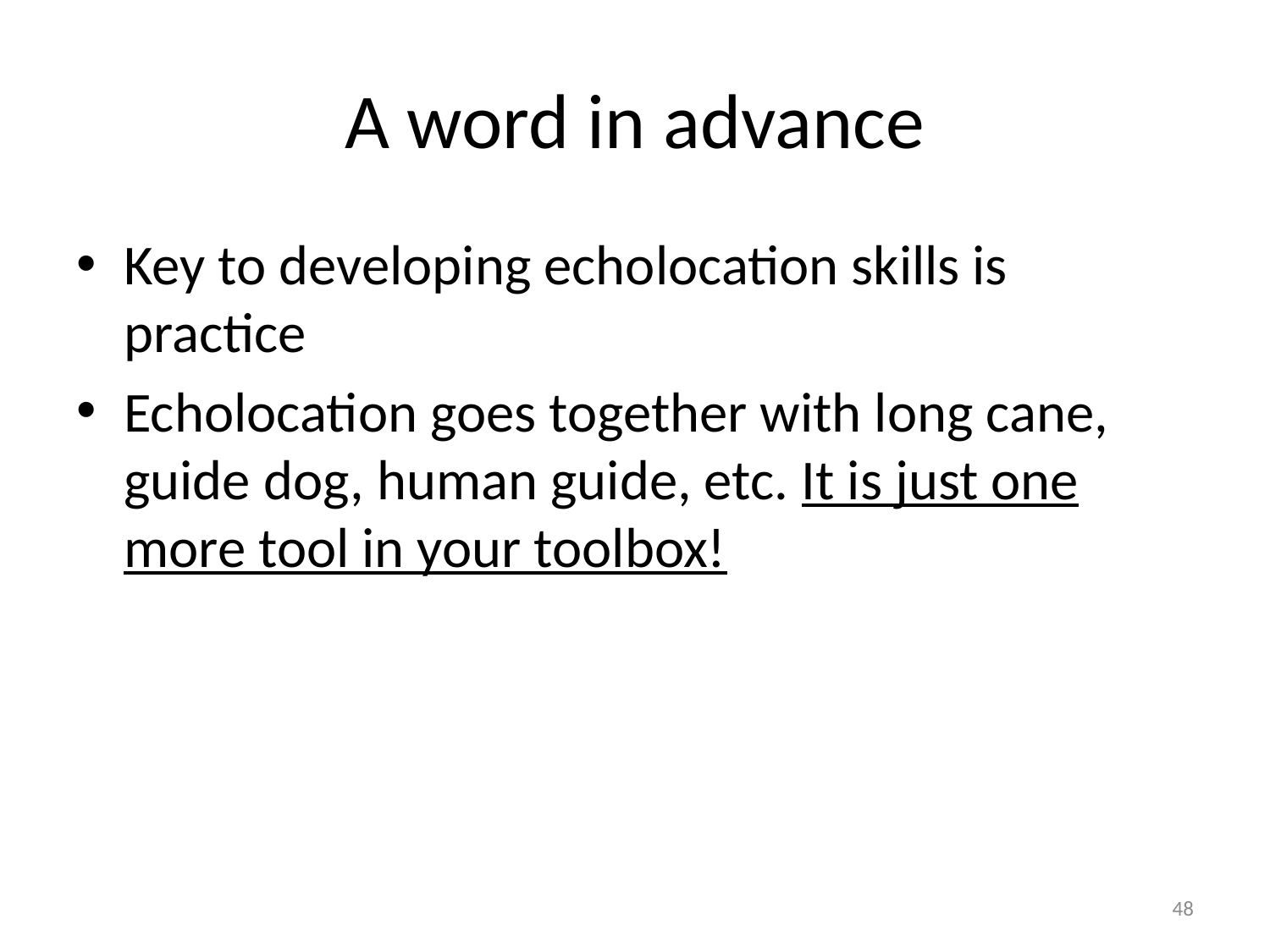

# A word in advance
Key to developing echolocation skills is practice
Echolocation goes together with long cane, guide dog, human guide, etc. It is just one more tool in your toolbox!
48

## Slide 49
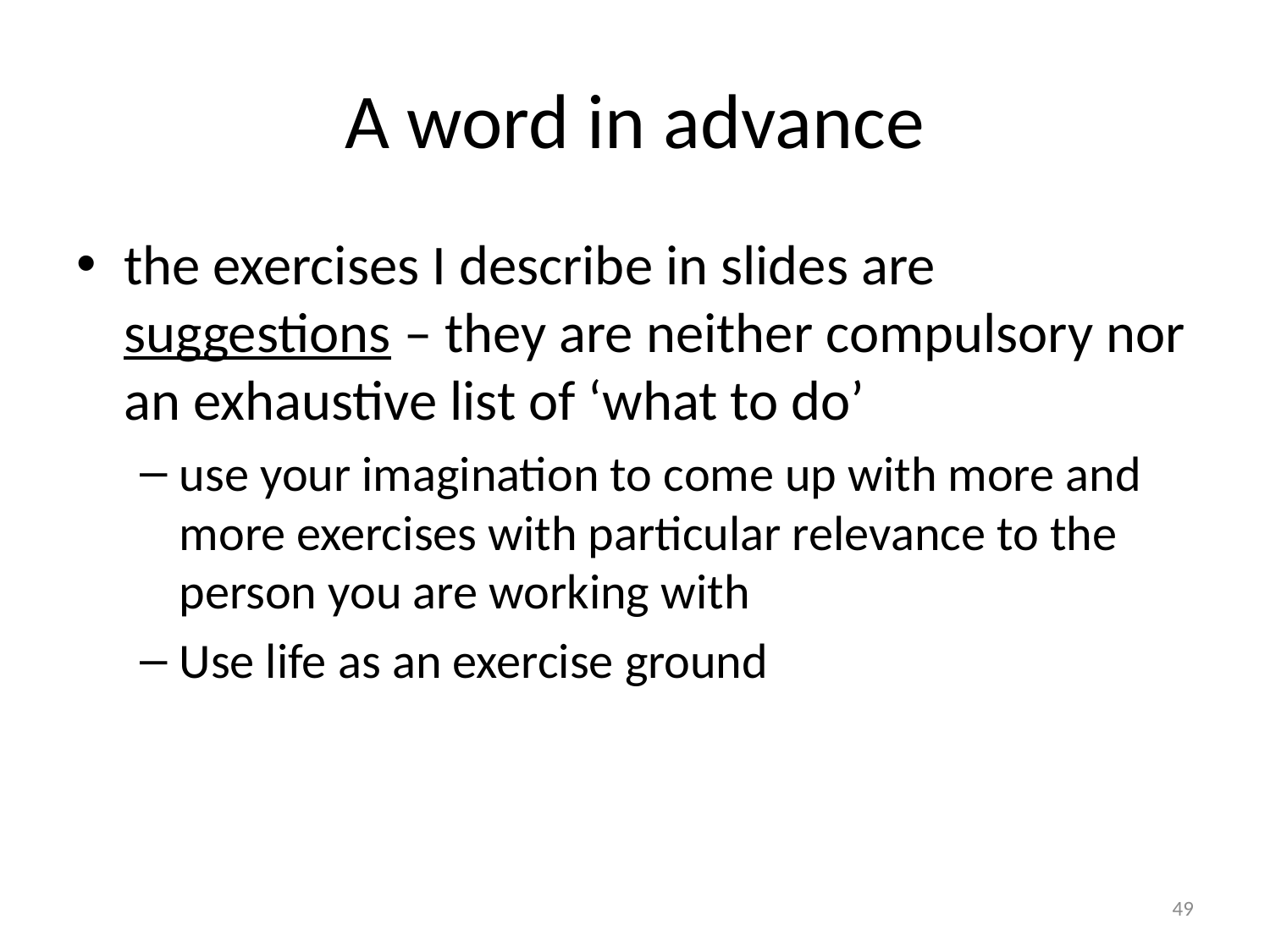

# A word in advance
the exercises I describe in slides are suggestions – they are neither compulsory nor an exhaustive list of ‘what to do’
use your imagination to come up with more and more exercises with particular relevance to the person you are working with
Use life as an exercise ground
49

## Slide 50
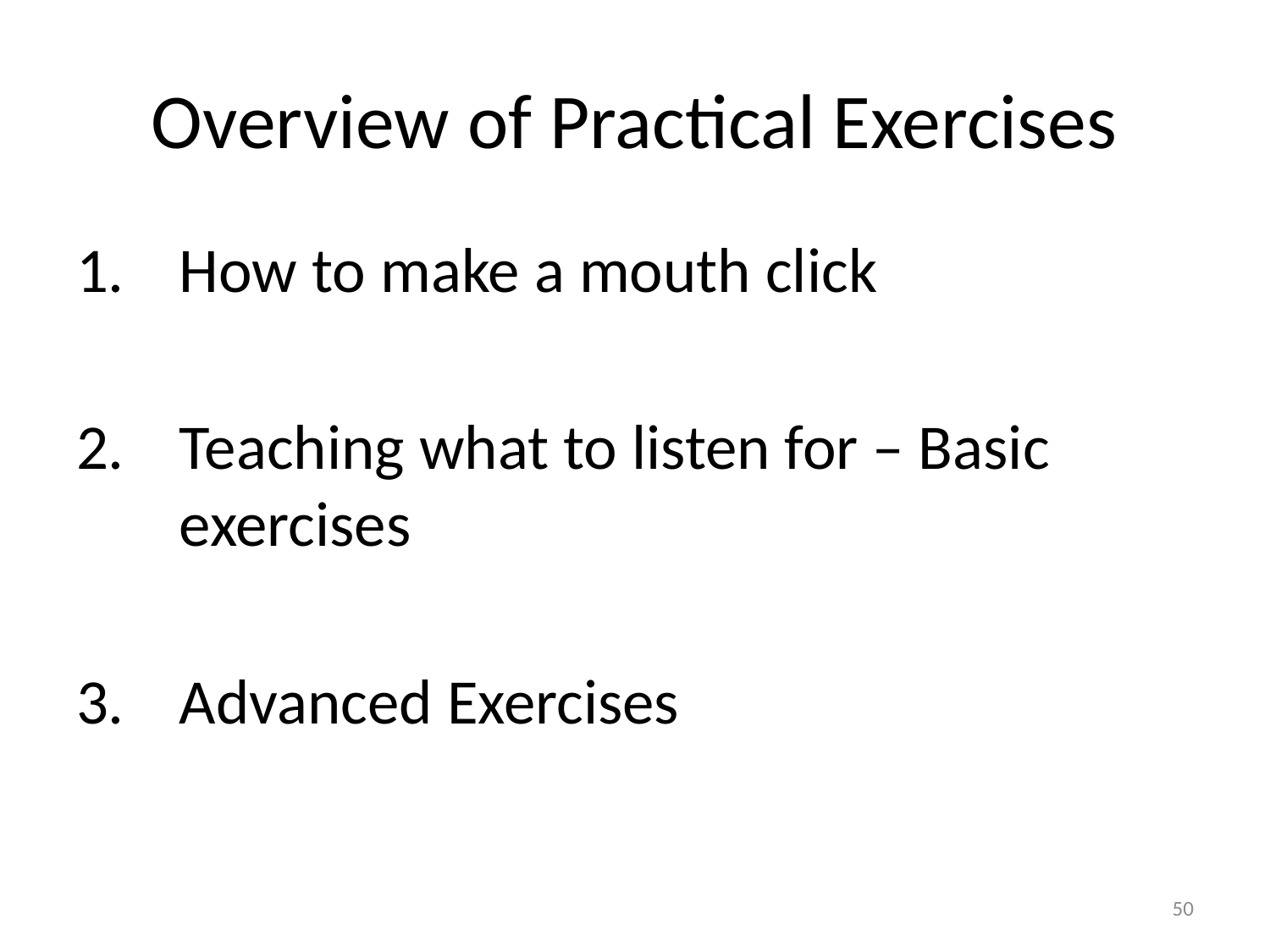

# Overview of Practical Exercises
How to make a mouth click
Teaching what to listen for – Basic exercises
Advanced Exercises
50

## Slide 51
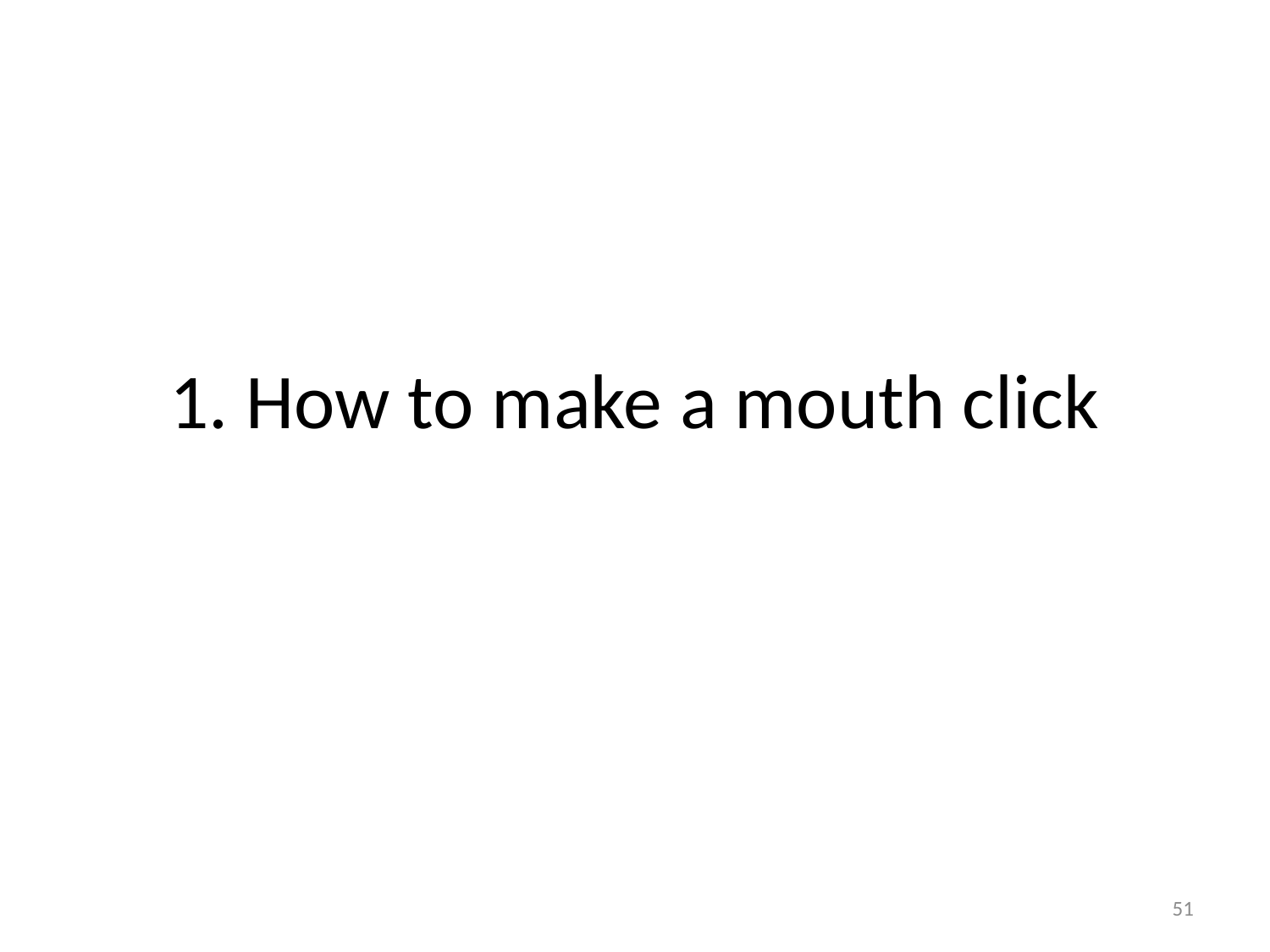

# 1. How to make a mouth click
51

## Slide 52
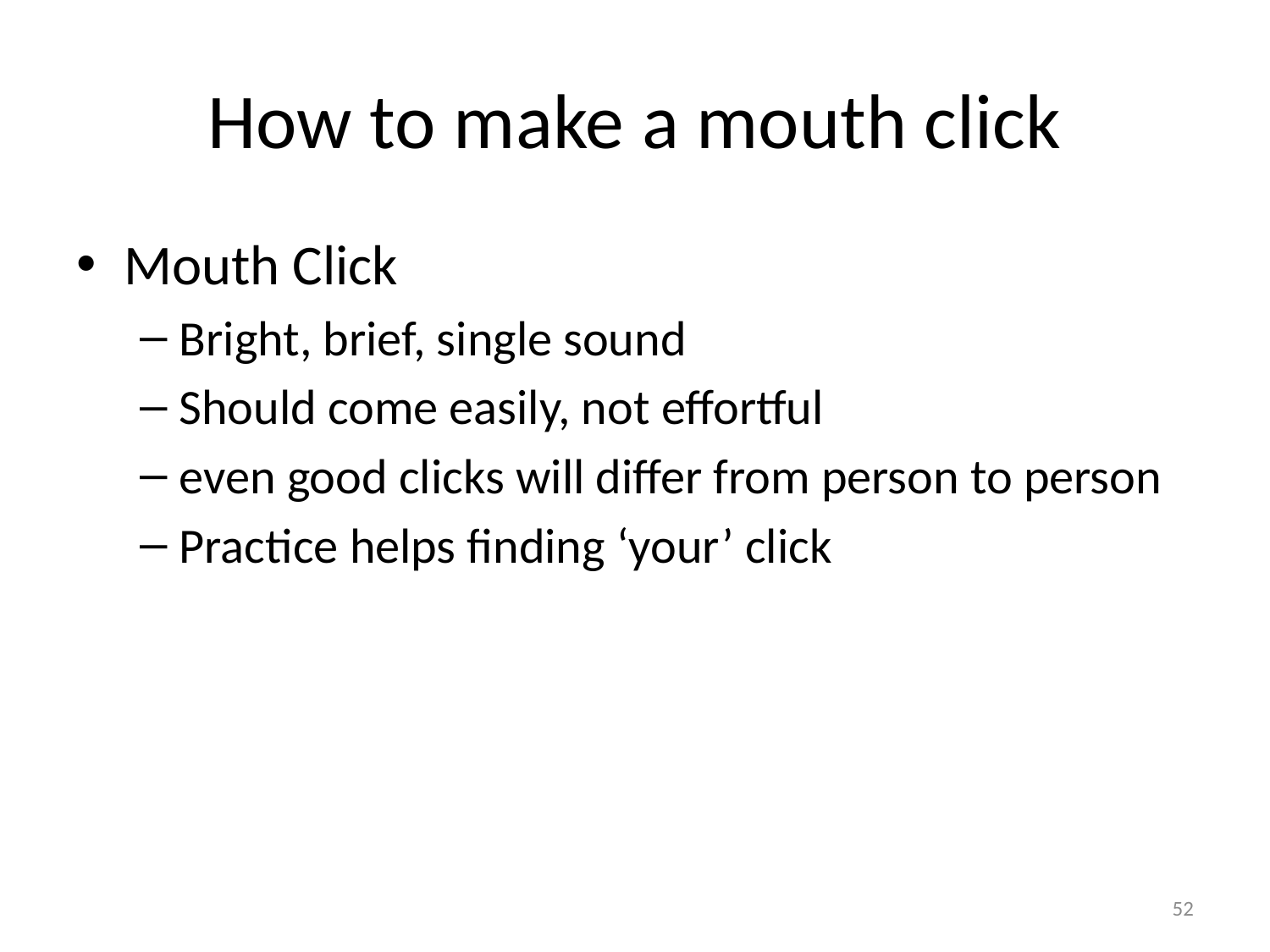

# How to make a mouth click
Mouth Click
Bright, brief, single sound
Should come easily, not effortful
even good clicks will differ from person to person
Practice helps finding ‘your’ click
52

## Slide 53
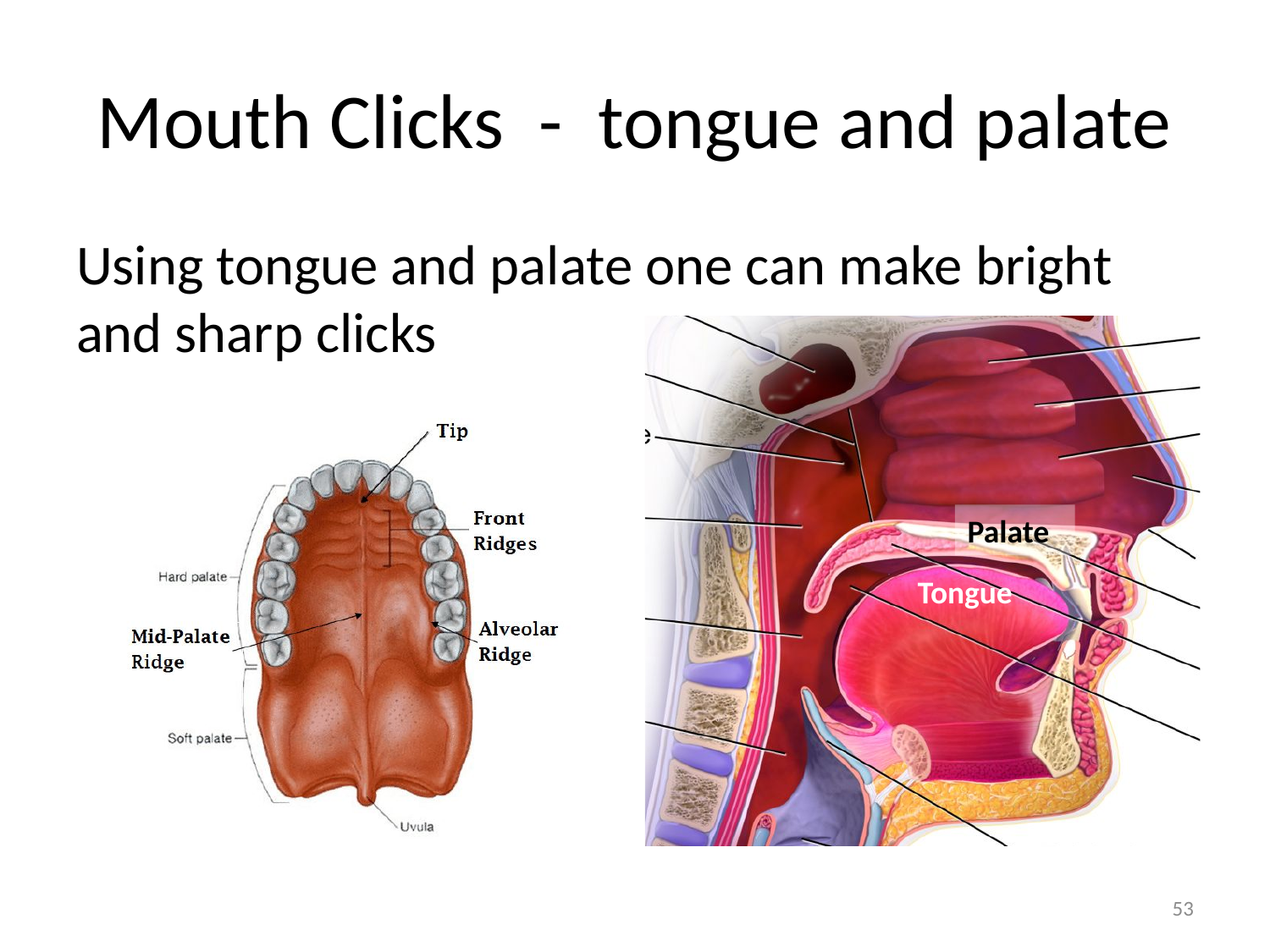

# Mouth Clicks - tongue and palate
Using tongue and palate one can make bright and sharp clicks
Palate
Tongue
53

## Slide 54
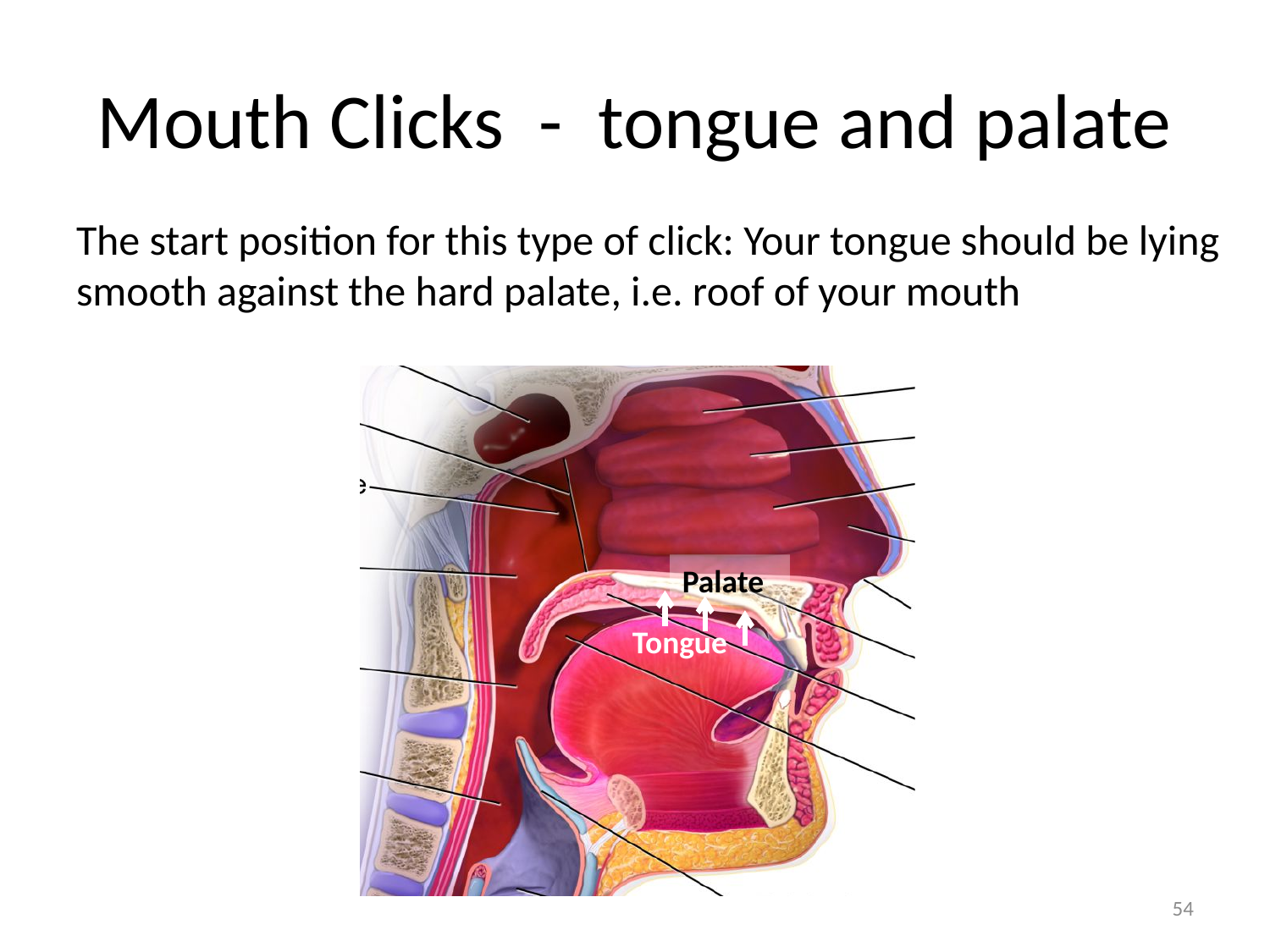

# Mouth Clicks - tongue and palate
The start position for this type of click: Your tongue should be lying smooth against the hard palate, i.e. roof of your mouth
Palate
Tongue
54

## Slide 55
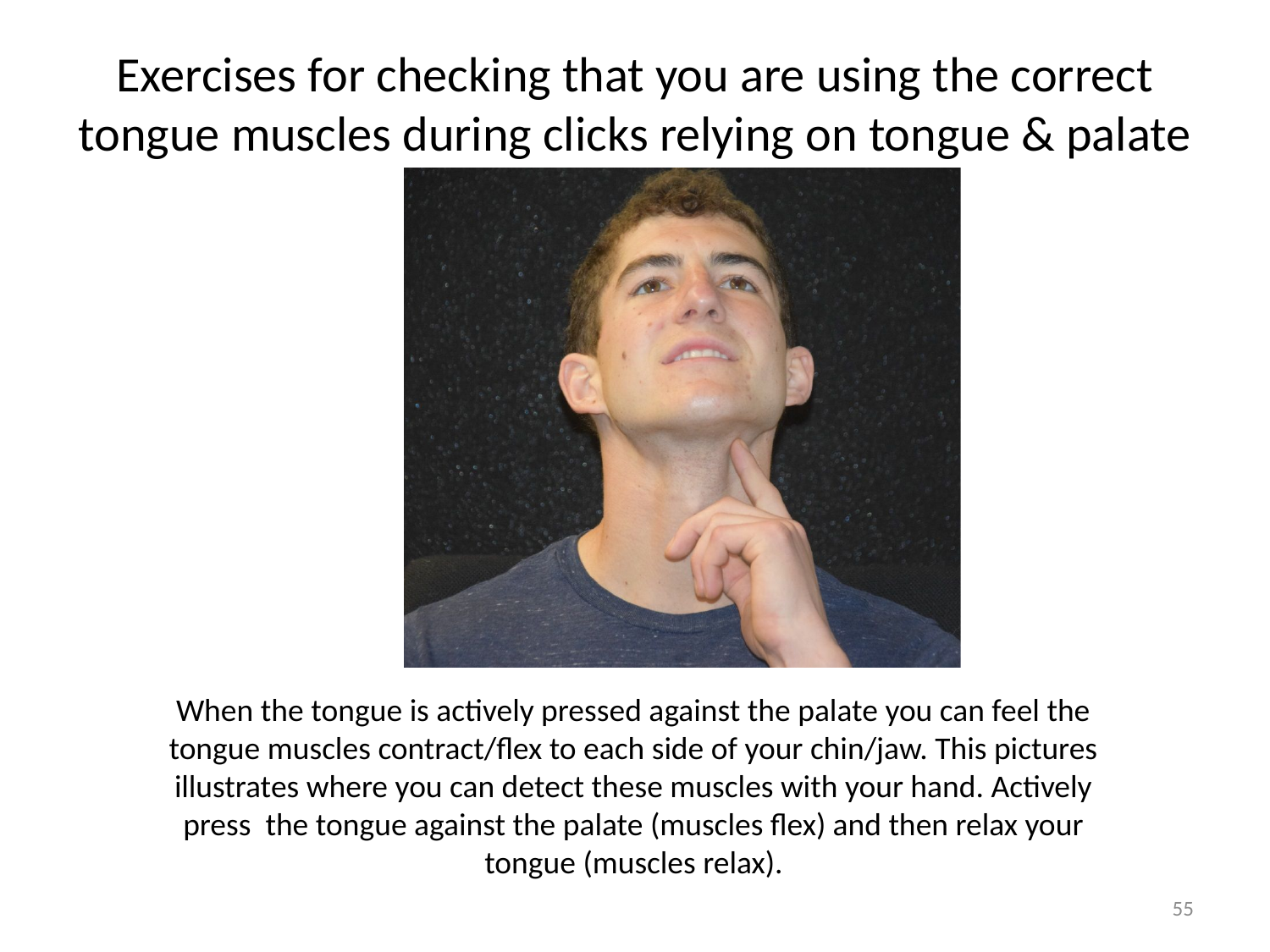

Exercises for checking that you are using the correct tongue muscles during clicks relying on tongue & palate
When the tongue is actively pressed against the palate you can feel the tongue muscles contract/flex to each side of your chin/jaw. This pictures illustrates where you can detect these muscles with your hand. Actively press the tongue against the palate (muscles flex) and then relax your tongue (muscles relax).
55

## Slide 56
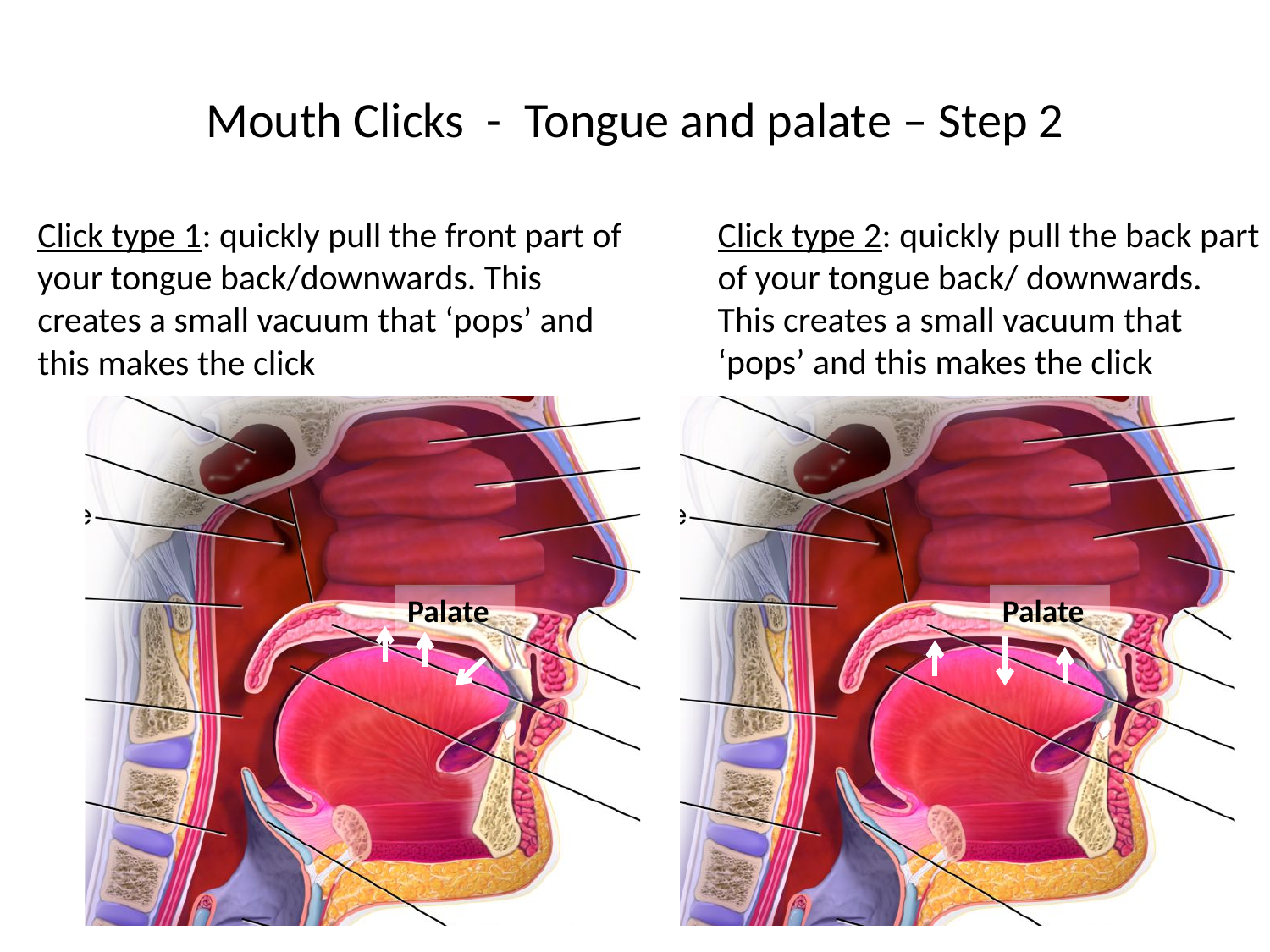

# Mouth Clicks - Tongue and palate – Step 2
Click type 1: quickly pull the front part of your tongue back/downwards. This creates a small vacuum that ‘pops’ and this makes the click
Click type 2: quickly pull the back part of your tongue back/ downwards. This creates a small vacuum that ‘pops’ and this makes the click
Palate
Palate
56

## Slide 57
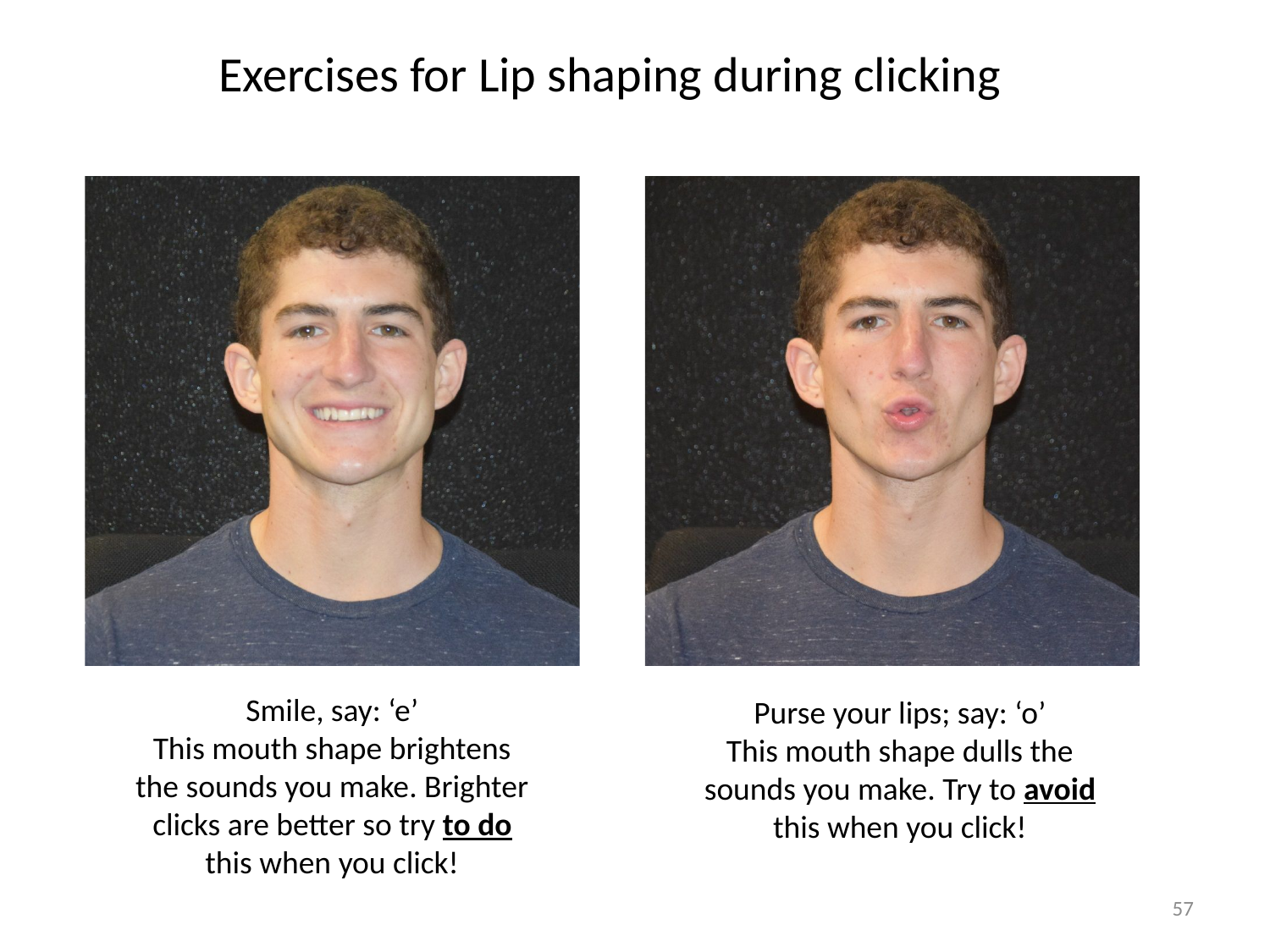

Exercises for Lip shaping during clicking
Smile, say: ‘e’
This mouth shape brightens the sounds you make. Brighter clicks are better so try to do this when you click!
Purse your lips; say: ‘o’
This mouth shape dulls the sounds you make. Try to avoid this when you click!
57

## Slide 58
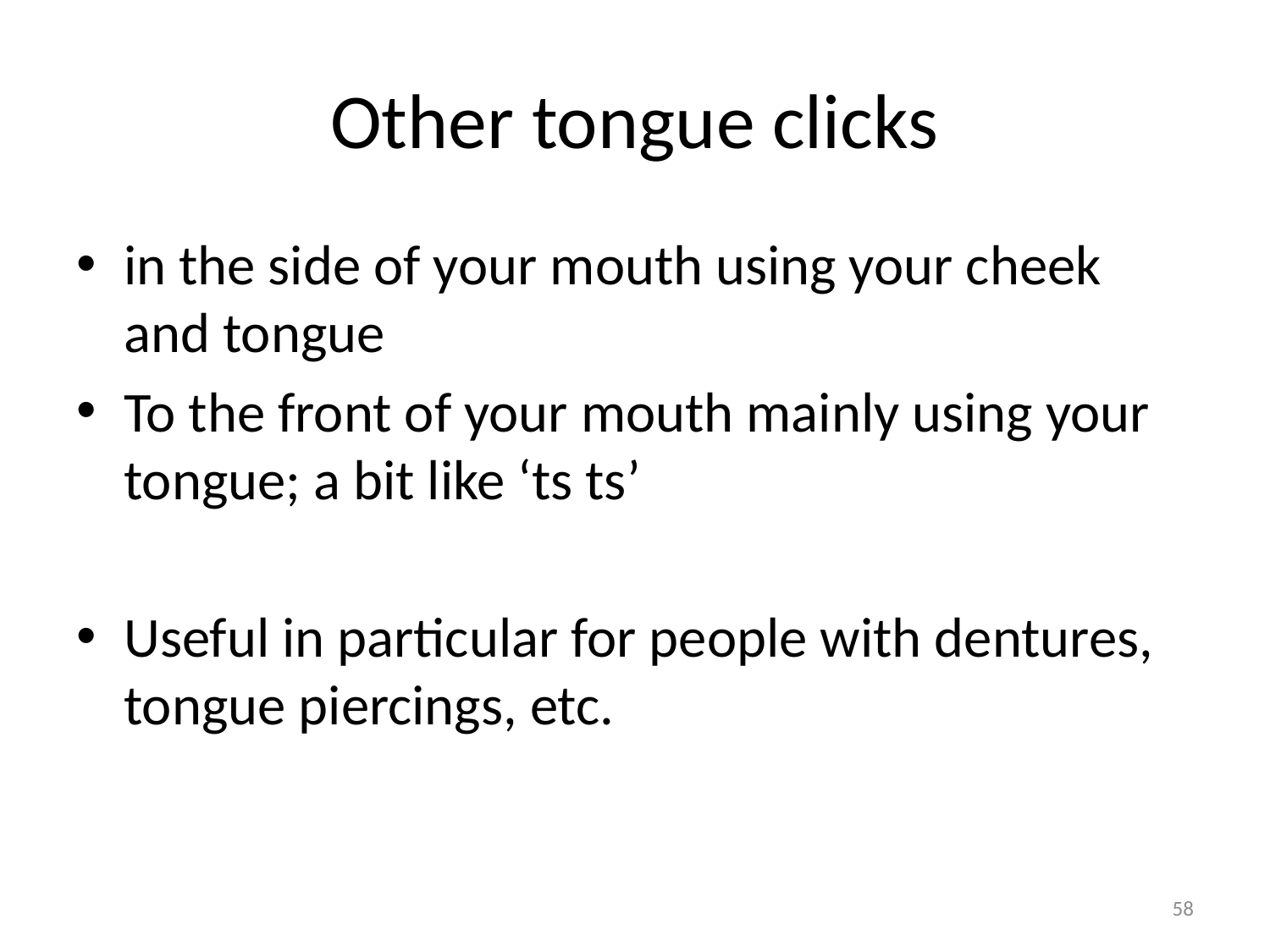

# Other tongue clicks
in the side of your mouth using your cheek and tongue
To the front of your mouth mainly using your tongue; a bit like ‘ts ts’
Useful in particular for people with dentures, tongue piercings, etc.
58

## Slide 59
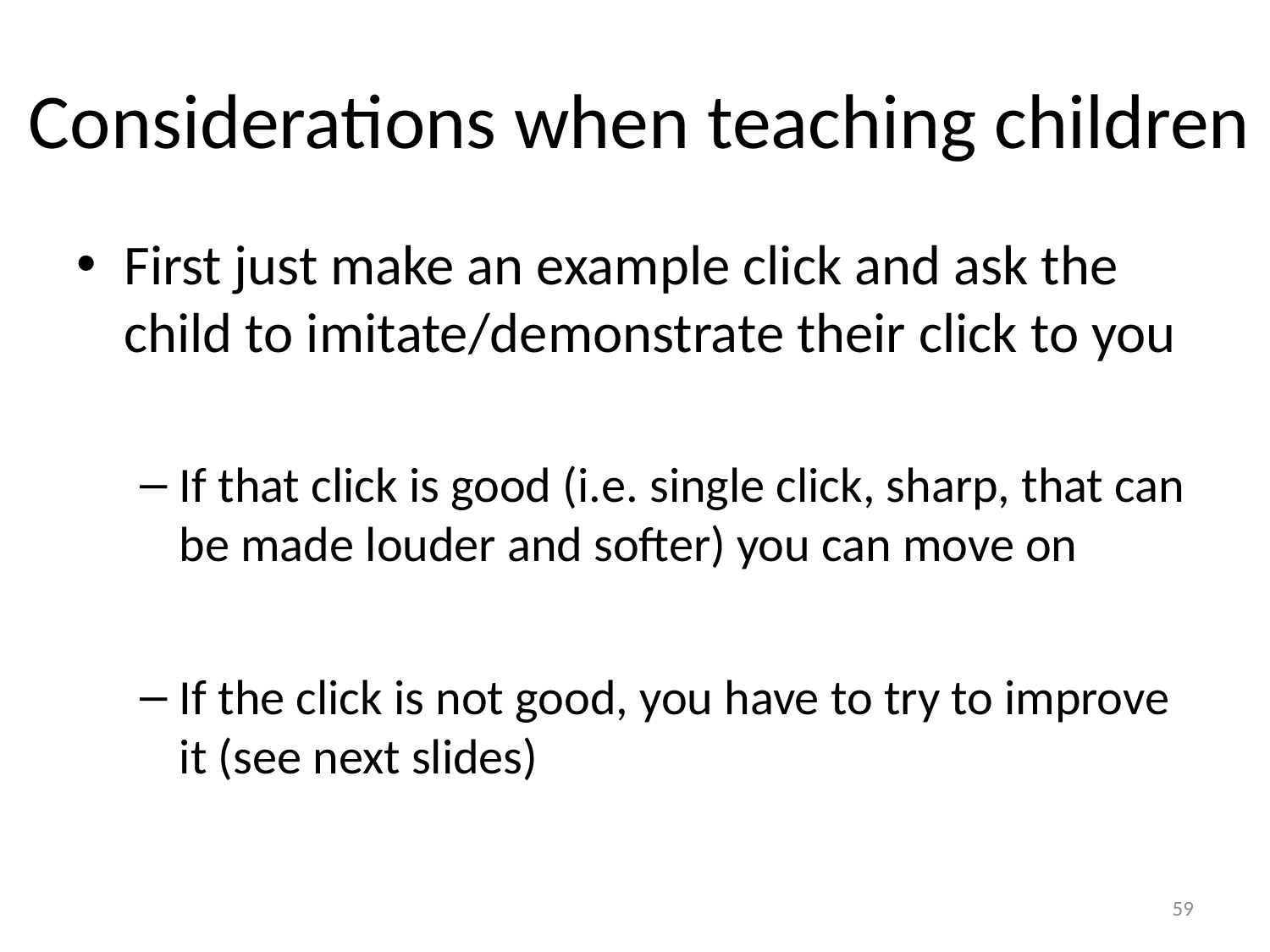

# Considerations when teaching children
First just make an example click and ask the child to imitate/demonstrate their click to you
If that click is good (i.e. single click, sharp, that can be made louder and softer) you can move on
If the click is not good, you have to try to improve it (see next slides)
59

## Slide 60
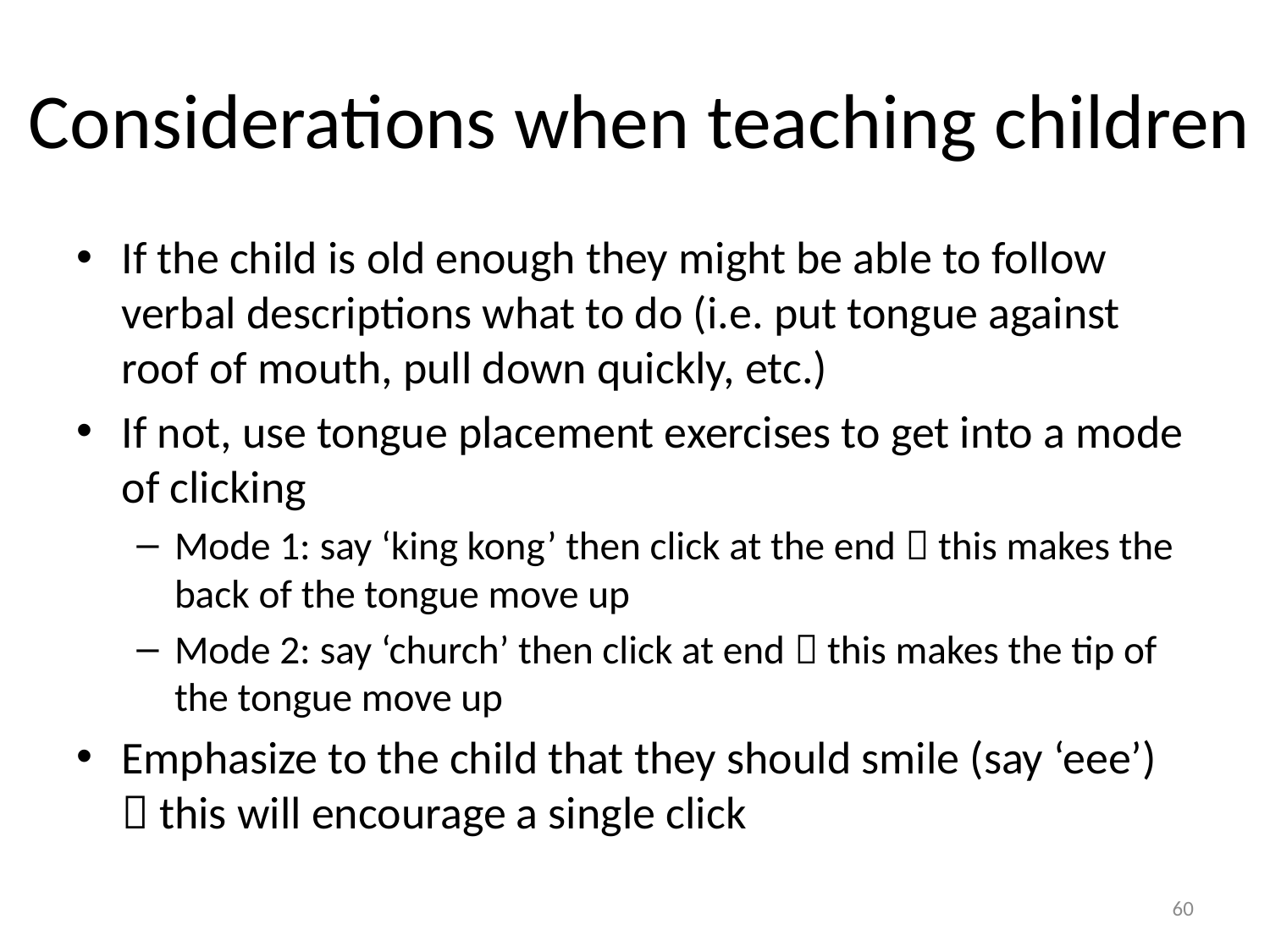

# Considerations when teaching children
If the child is old enough they might be able to follow verbal descriptions what to do (i.e. put tongue against roof of mouth, pull down quickly, etc.)
If not, use tongue placement exercises to get into a mode of clicking
Mode 1: say ‘king kong’ then click at the end  this makes the back of the tongue move up
Mode 2: say ‘church’ then click at end  this makes the tip of the tongue move up
Emphasize to the child that they should smile (say ‘eee’)  this will encourage a single click
60

## Slide 61
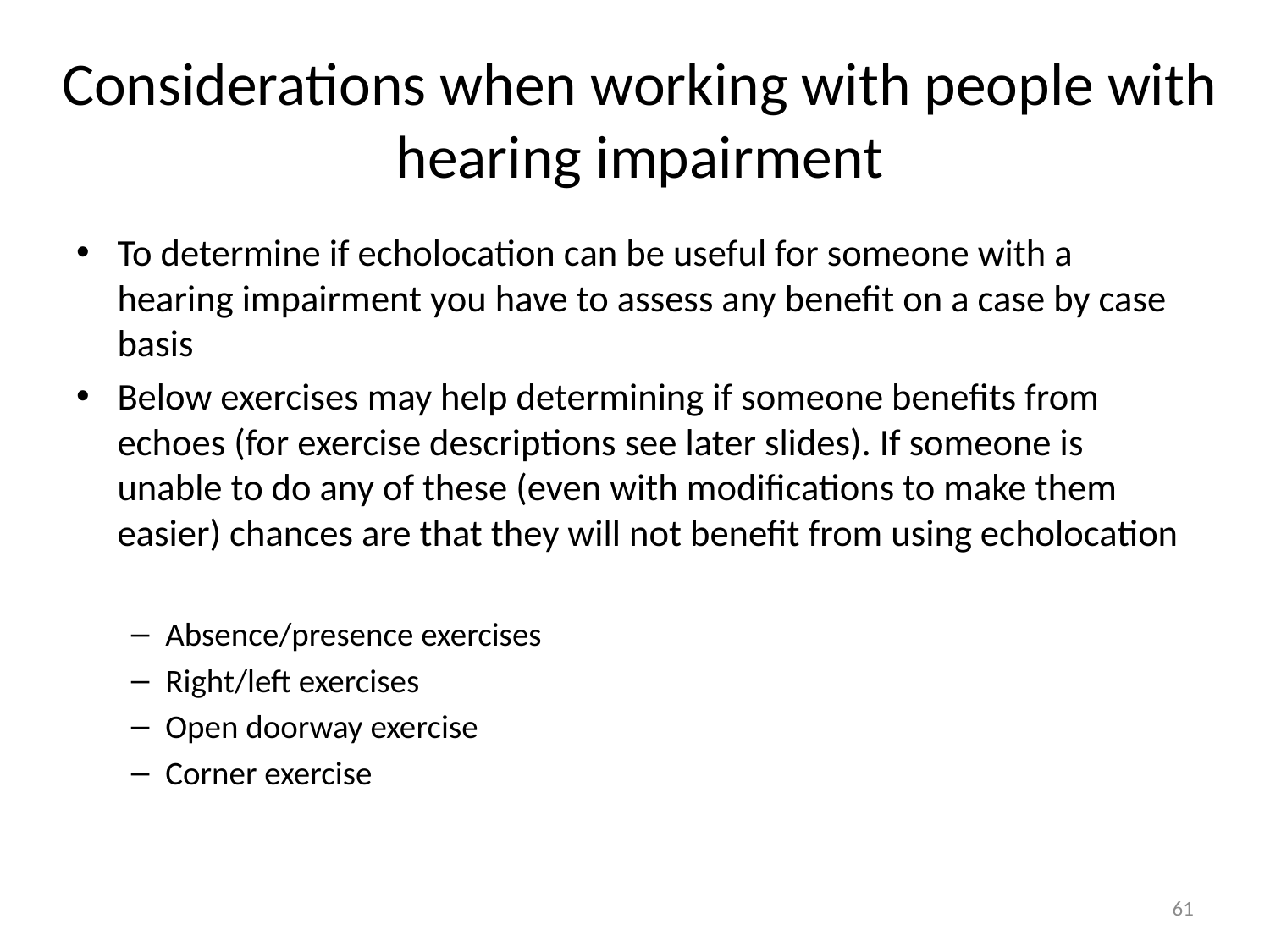

# Considerations when working with people with hearing impairment
To determine if echolocation can be useful for someone with a hearing impairment you have to assess any benefit on a case by case basis
Below exercises may help determining if someone benefits from echoes (for exercise descriptions see later slides). If someone is unable to do any of these (even with modifications to make them easier) chances are that they will not benefit from using echolocation
Absence/presence exercises
Right/left exercises
Open doorway exercise
Corner exercise
61

## Slide 62
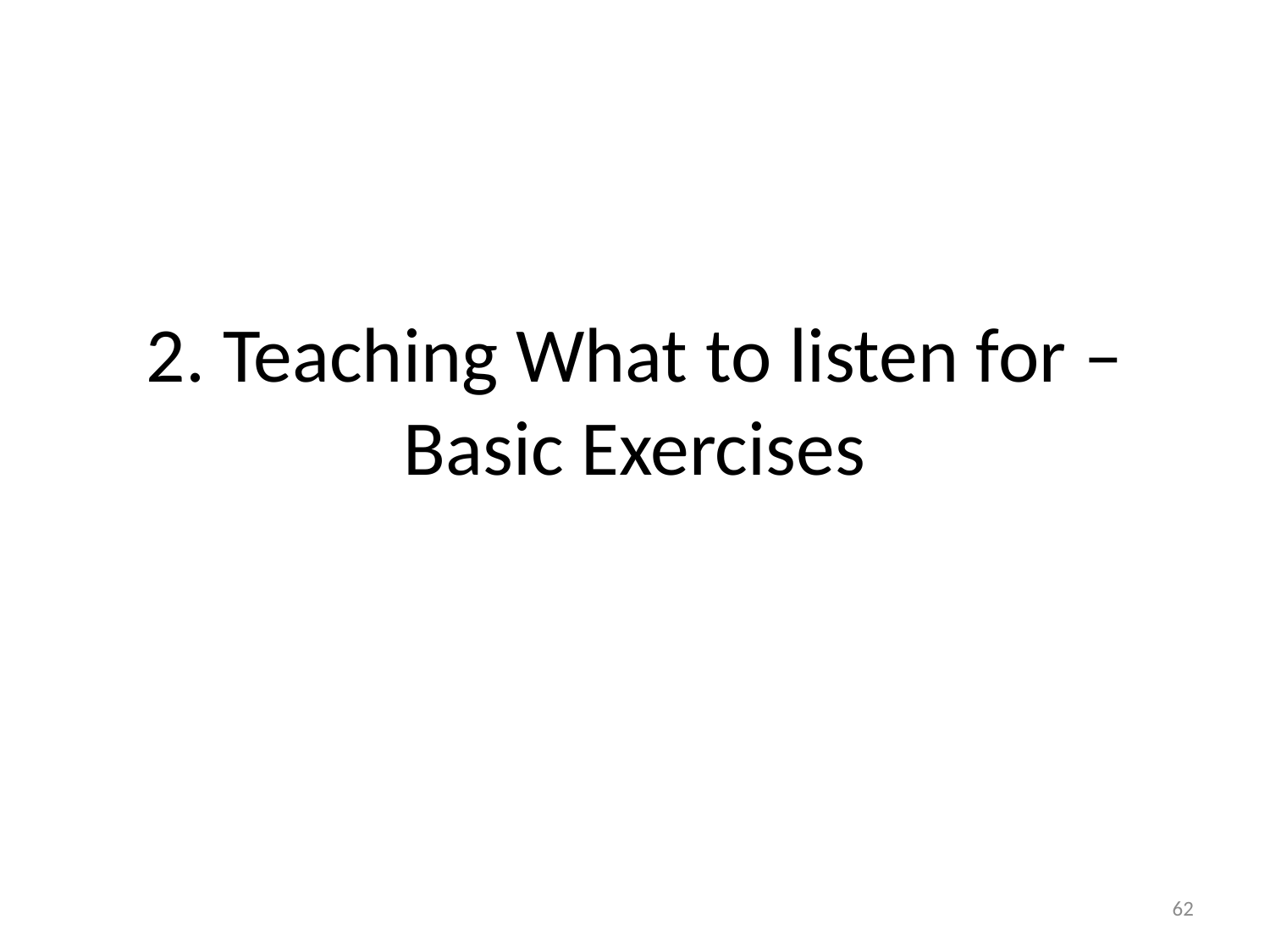

# 2. Teaching What to listen for – Basic Exercises
62

## Slide 63
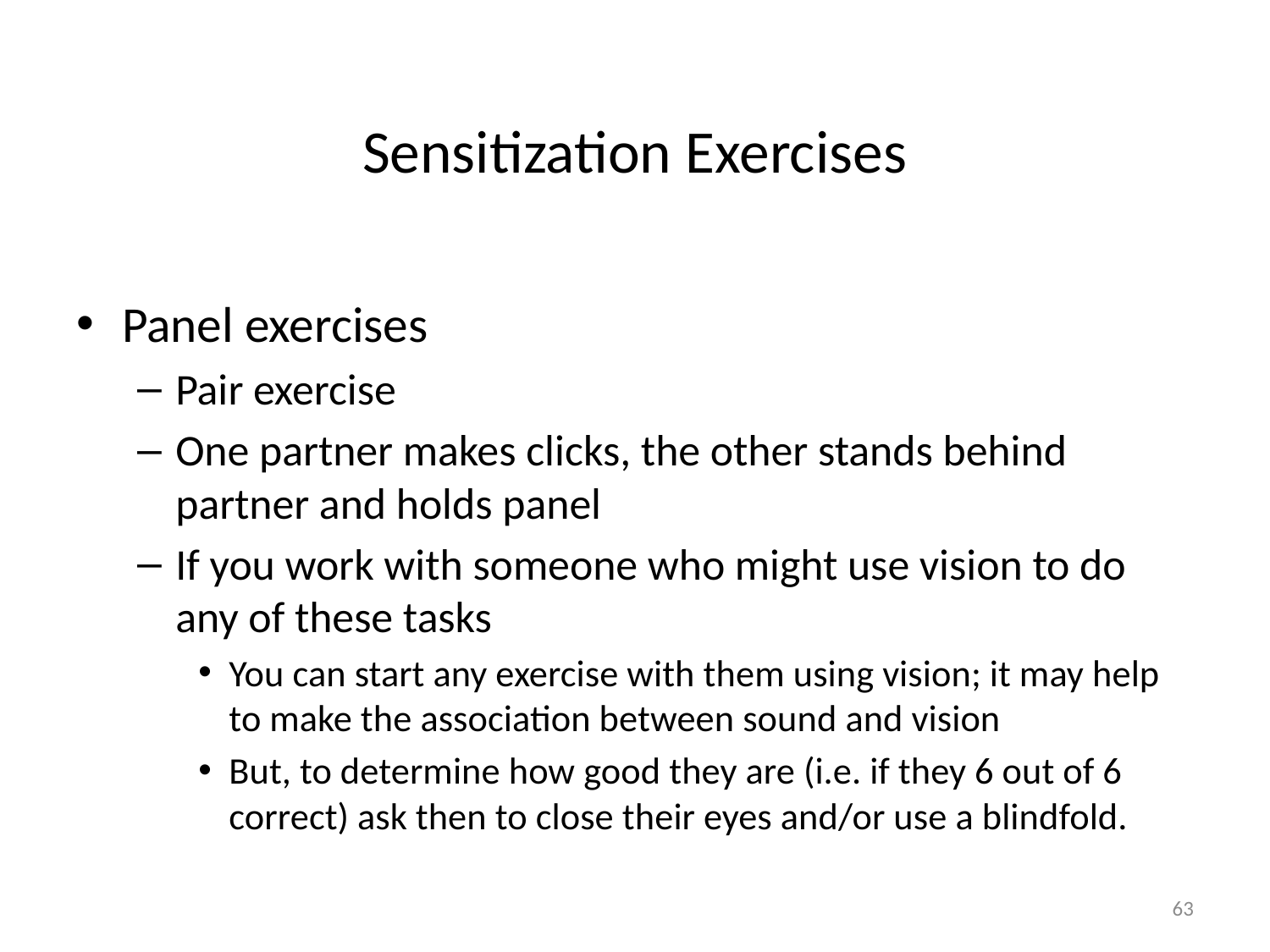

# Sensitization Exercises
Panel exercises
Pair exercise
One partner makes clicks, the other stands behind partner and holds panel
If you work with someone who might use vision to do any of these tasks
You can start any exercise with them using vision; it may help to make the association between sound and vision
But, to determine how good they are (i.e. if they 6 out of 6 correct) ask then to close their eyes and/or use a blindfold.
63

## Slide 64
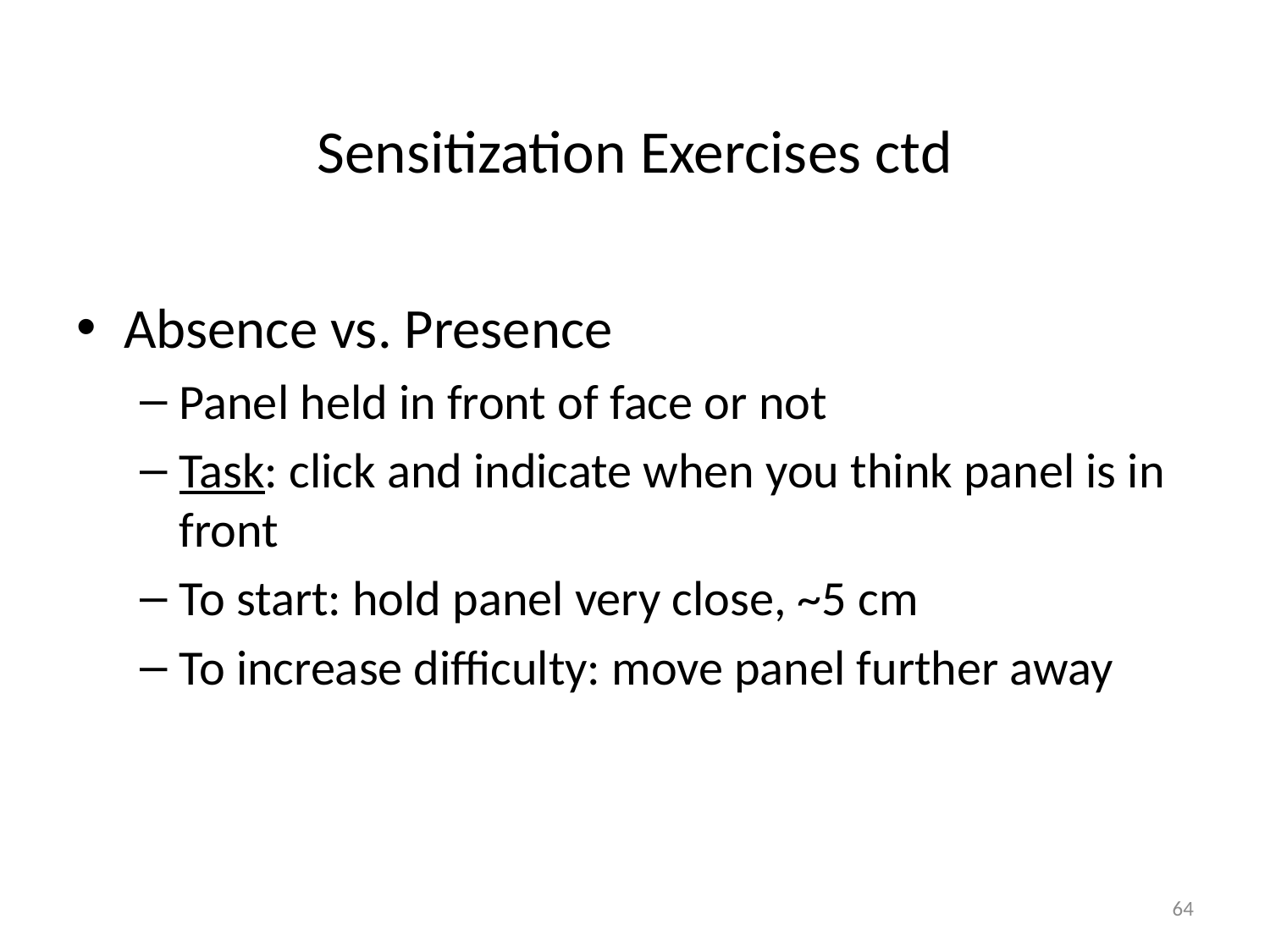

# Sensitization Exercises ctd
Absence vs. Presence
Panel held in front of face or not
Task: click and indicate when you think panel is in front
To start: hold panel very close, ~5 cm
To increase difficulty: move panel further away
64

## Slide 65
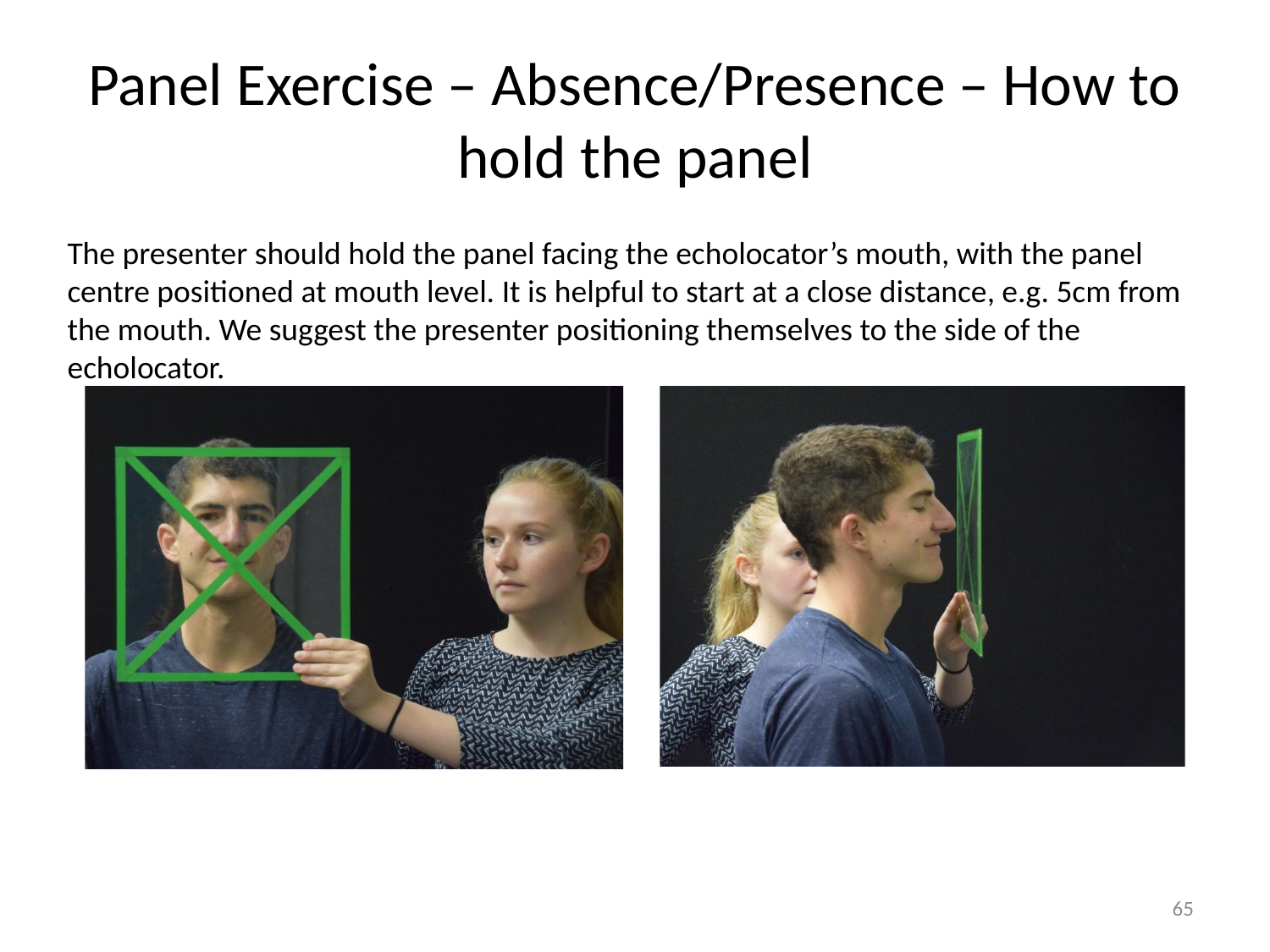

# Panel Exercise – Absence/Presence – How to hold the panel
The presenter should hold the panel facing the echolocator’s mouth, with the panel centre positioned at mouth level. It is helpful to start at a close distance, e.g. 5cm from the mouth. We suggest the presenter positioning themselves to the side of the echolocator.
65

## Slide 66
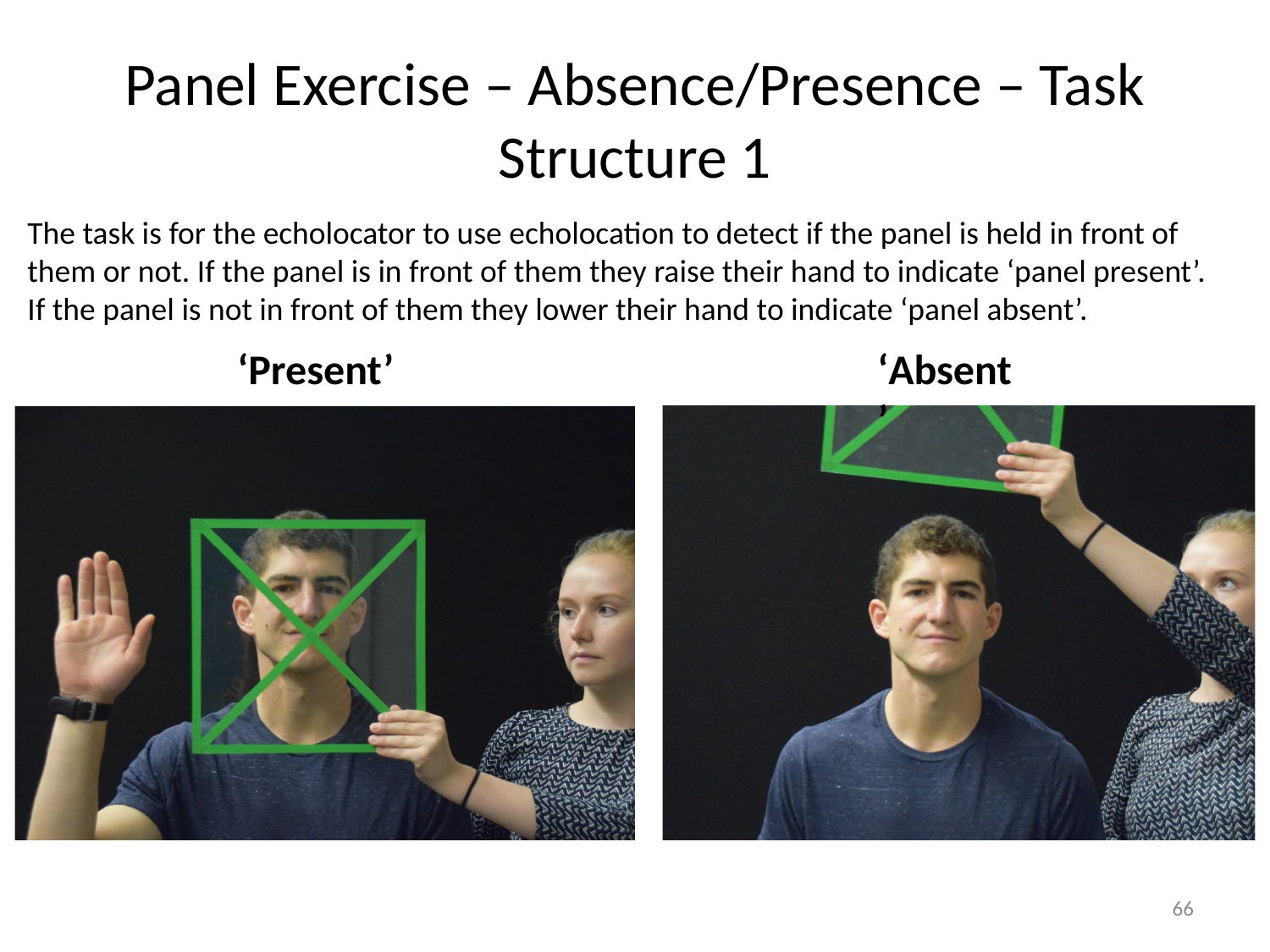

# Panel Exercise – Absence/Presence – Task Structure 1
The task is for the echolocator to use echolocation to detect if the panel is held in front of them or not. If the panel is in front of them they raise their hand to indicate ‘panel present’. If the panel is not in front of them they lower their hand to indicate ‘panel absent’.
‘Present’
‘Absent’
66

## Slide 67
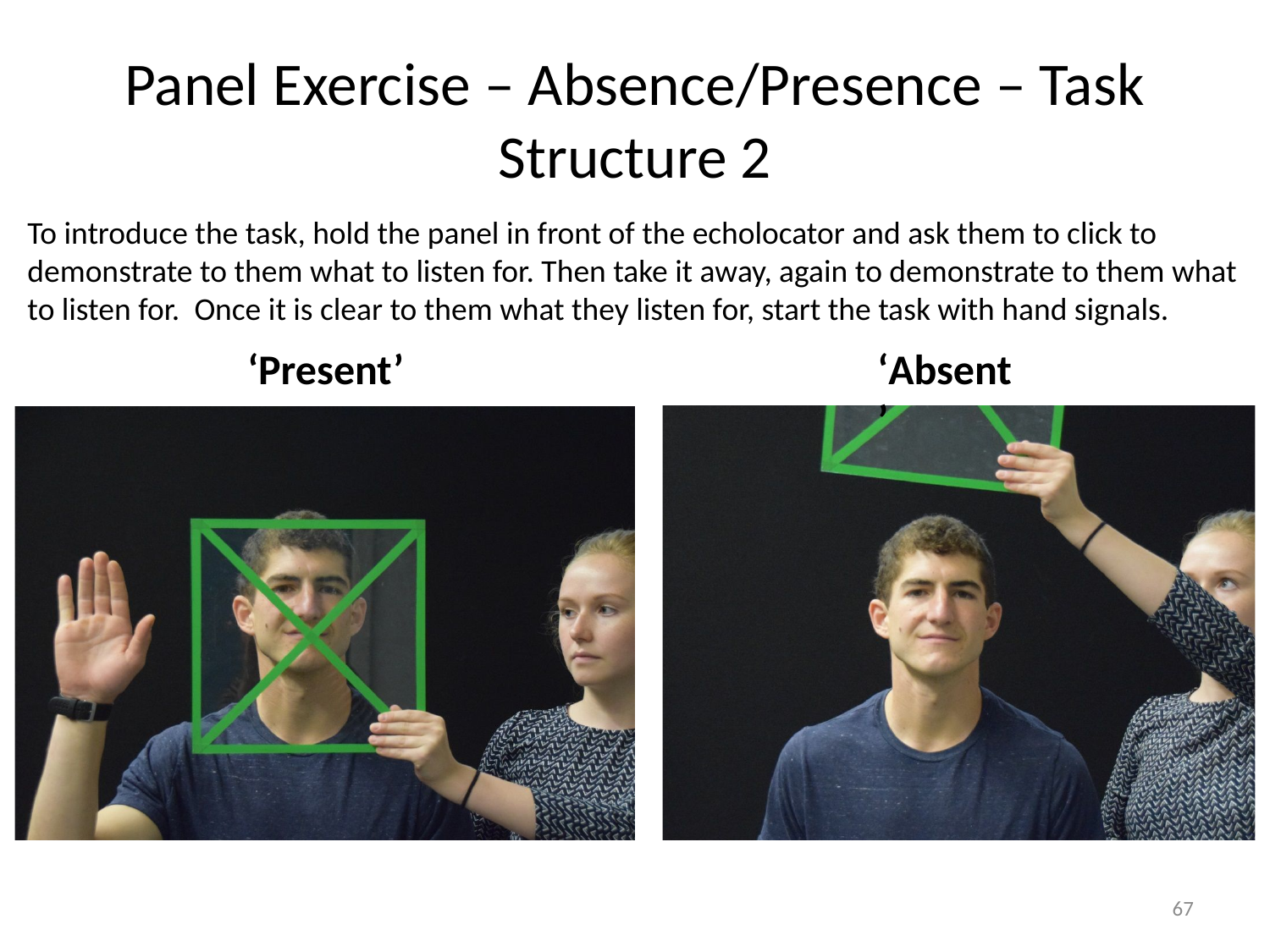

# Panel Exercise – Absence/Presence – Task Structure 2
To introduce the task, hold the panel in front of the echolocator and ask them to click to demonstrate to them what to listen for. Then take it away, again to demonstrate to them what to listen for. Once it is clear to them what they listen for, start the task with hand signals.
‘Present’
‘Absent’
67

## Slide 68
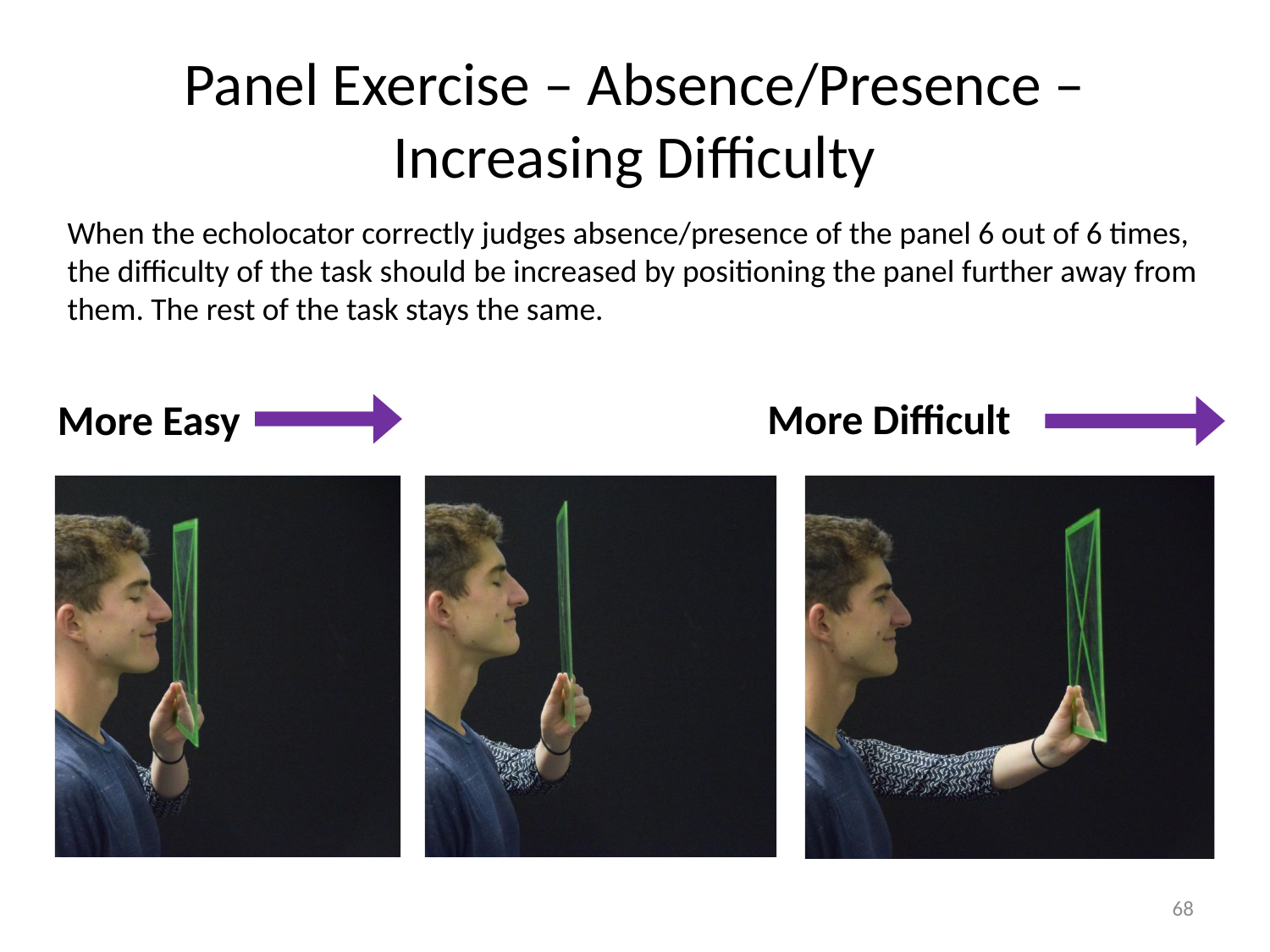

# Panel Exercise – Absence/Presence – Increasing Difficulty
When the echolocator correctly judges absence/presence of the panel 6 out of 6 times, the difficulty of the task should be increased by positioning the panel further away from them. The rest of the task stays the same.
More Difficult
More Easy
68

## Slide 69
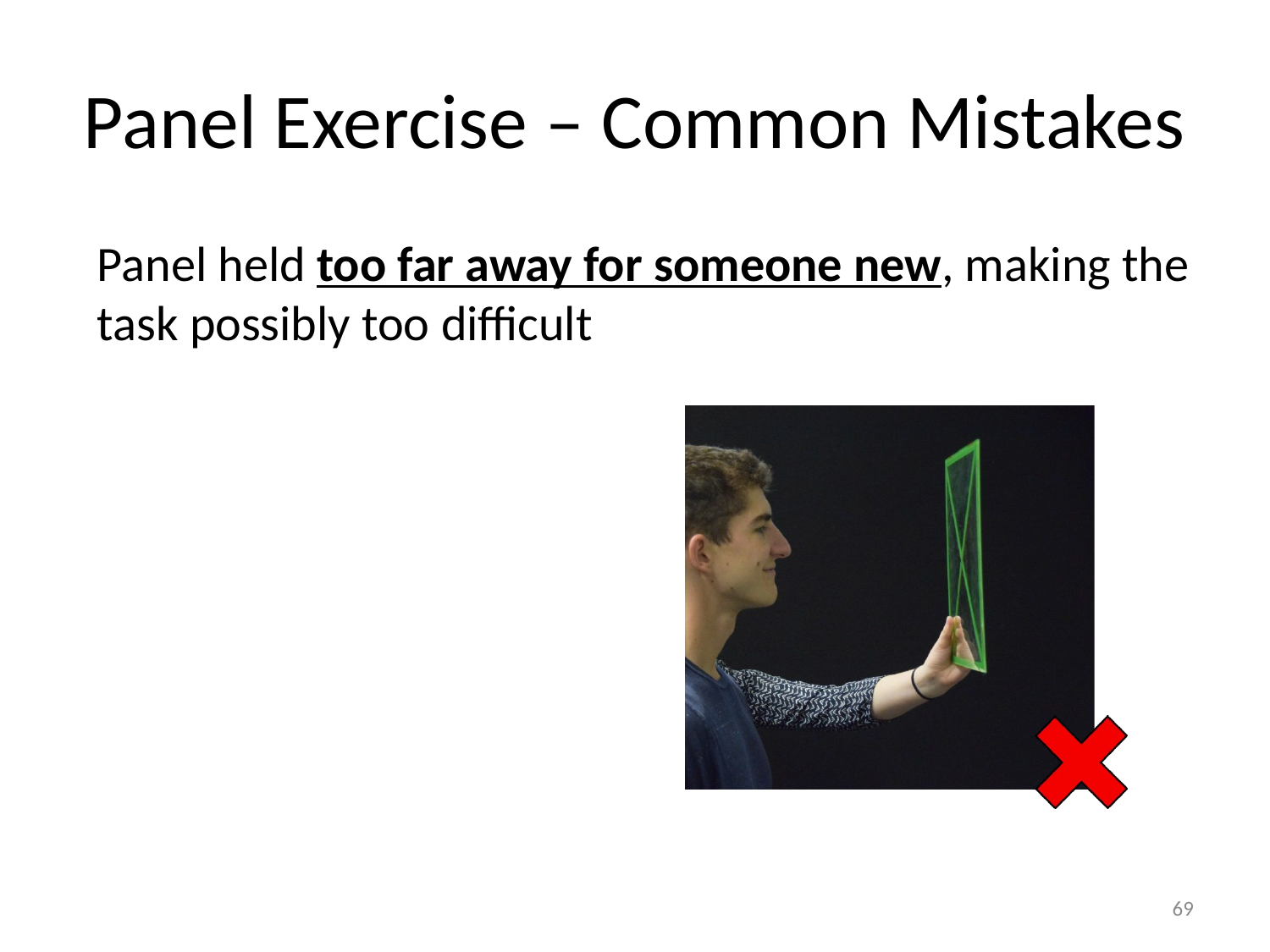

# Panel Exercise – Common Mistakes
Panel held too far away for someone new, making the task possibly too difficult
69

## Slide 70
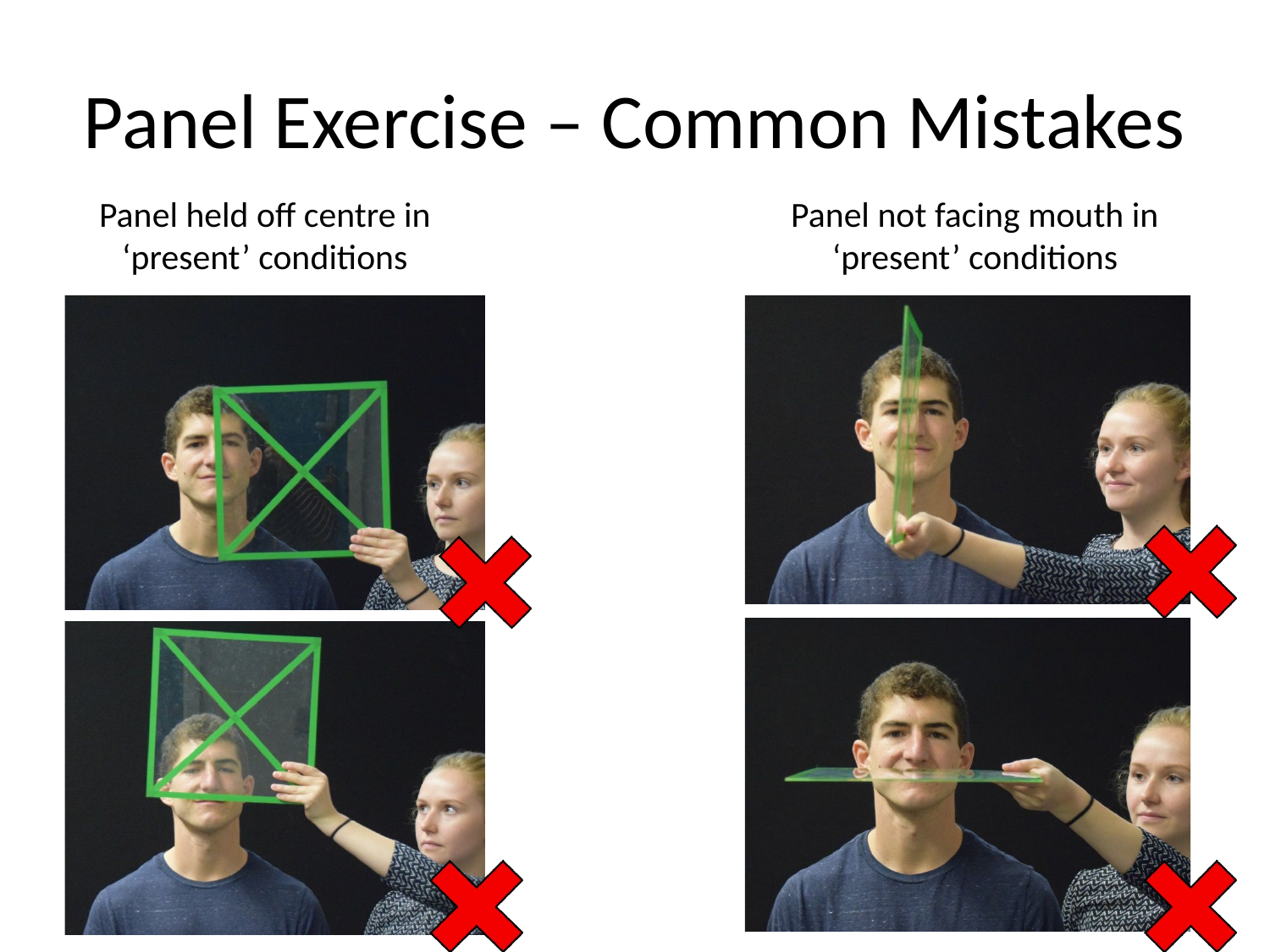

# Panel Exercise – Common Mistakes
Panel held off centre in ‘present’ conditions
Panel not facing mouth in ‘present’ conditions
70

## Slide 71
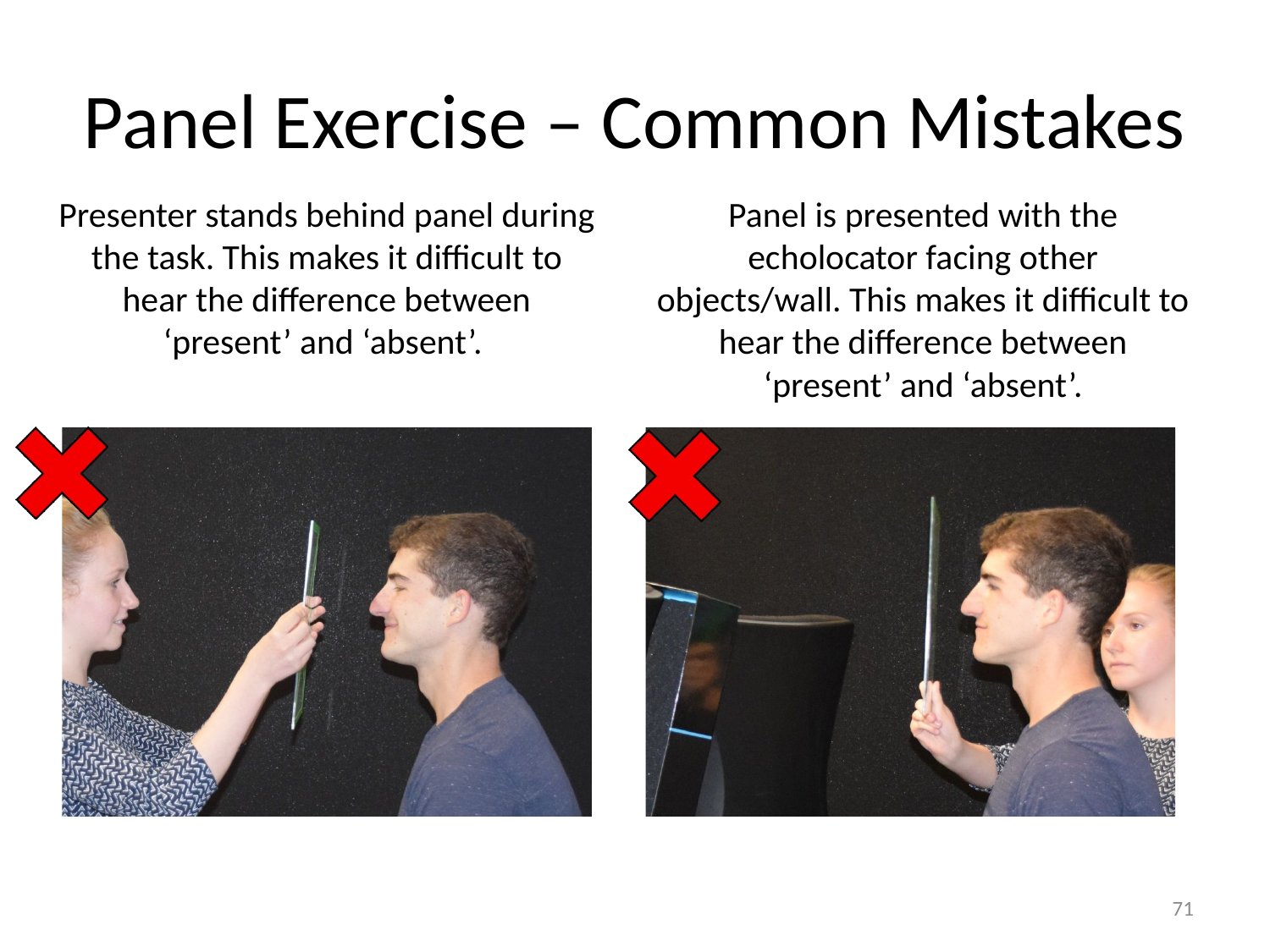

# Panel Exercise – Common Mistakes
Presenter stands behind panel during the task. This makes it difficult to hear the difference between ‘present’ and ‘absent’.
Panel is presented with the echolocator facing other objects/wall. This makes it difficult to hear the difference between ‘present’ and ‘absent’.
71

## Slide 72
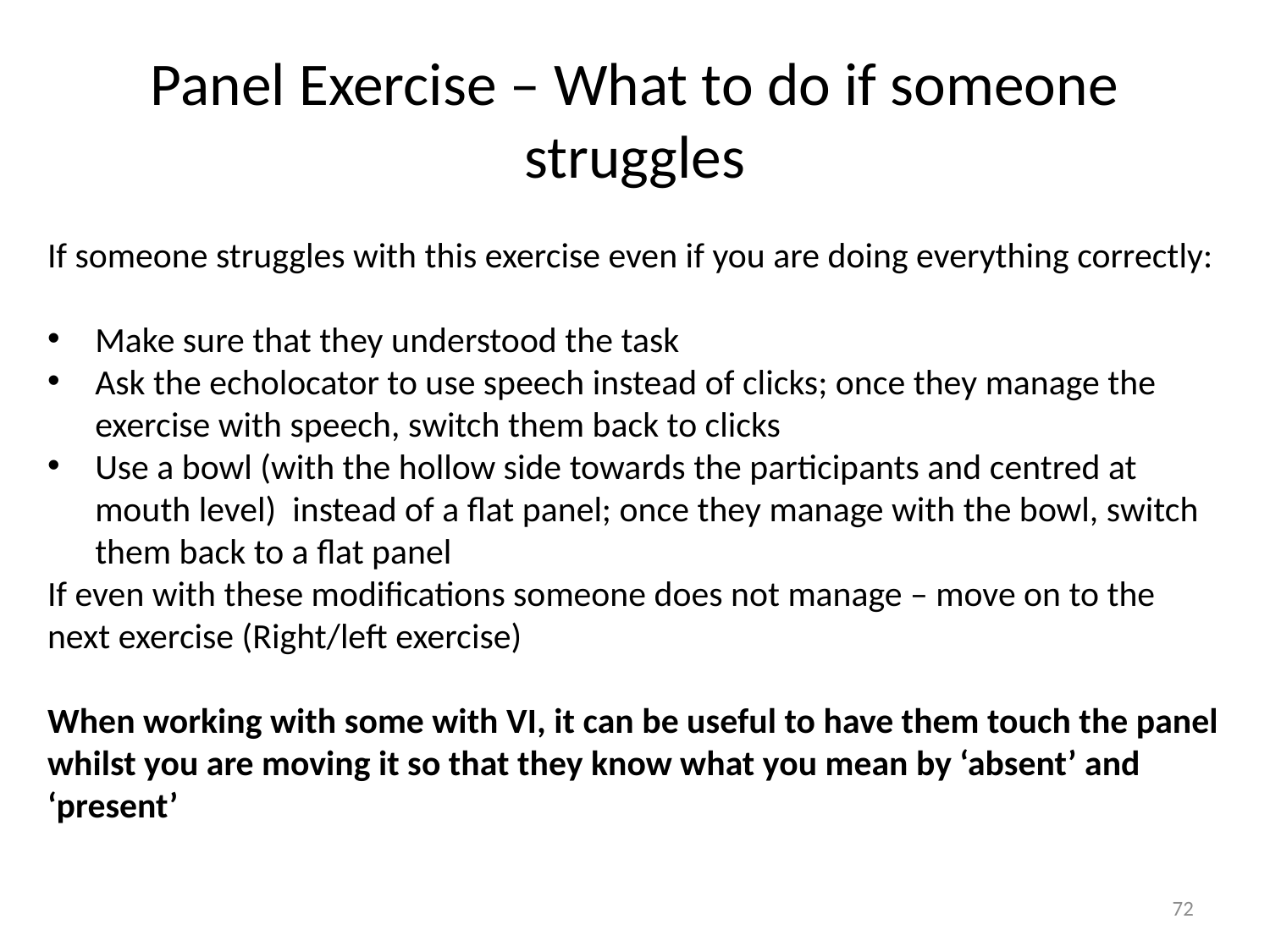

# Panel Exercise – What to do if someone struggles
If someone struggles with this exercise even if you are doing everything correctly:
Make sure that they understood the task
Ask the echolocator to use speech instead of clicks; once they manage the exercise with speech, switch them back to clicks
Use a bowl (with the hollow side towards the participants and centred at mouth level) instead of a flat panel; once they manage with the bowl, switch them back to a flat panel
If even with these modifications someone does not manage – move on to the next exercise (Right/left exercise)
When working with some with VI, it can be useful to have them touch the panel whilst you are moving it so that they know what you mean by ‘absent’ and ‘present’
72

## Slide 73
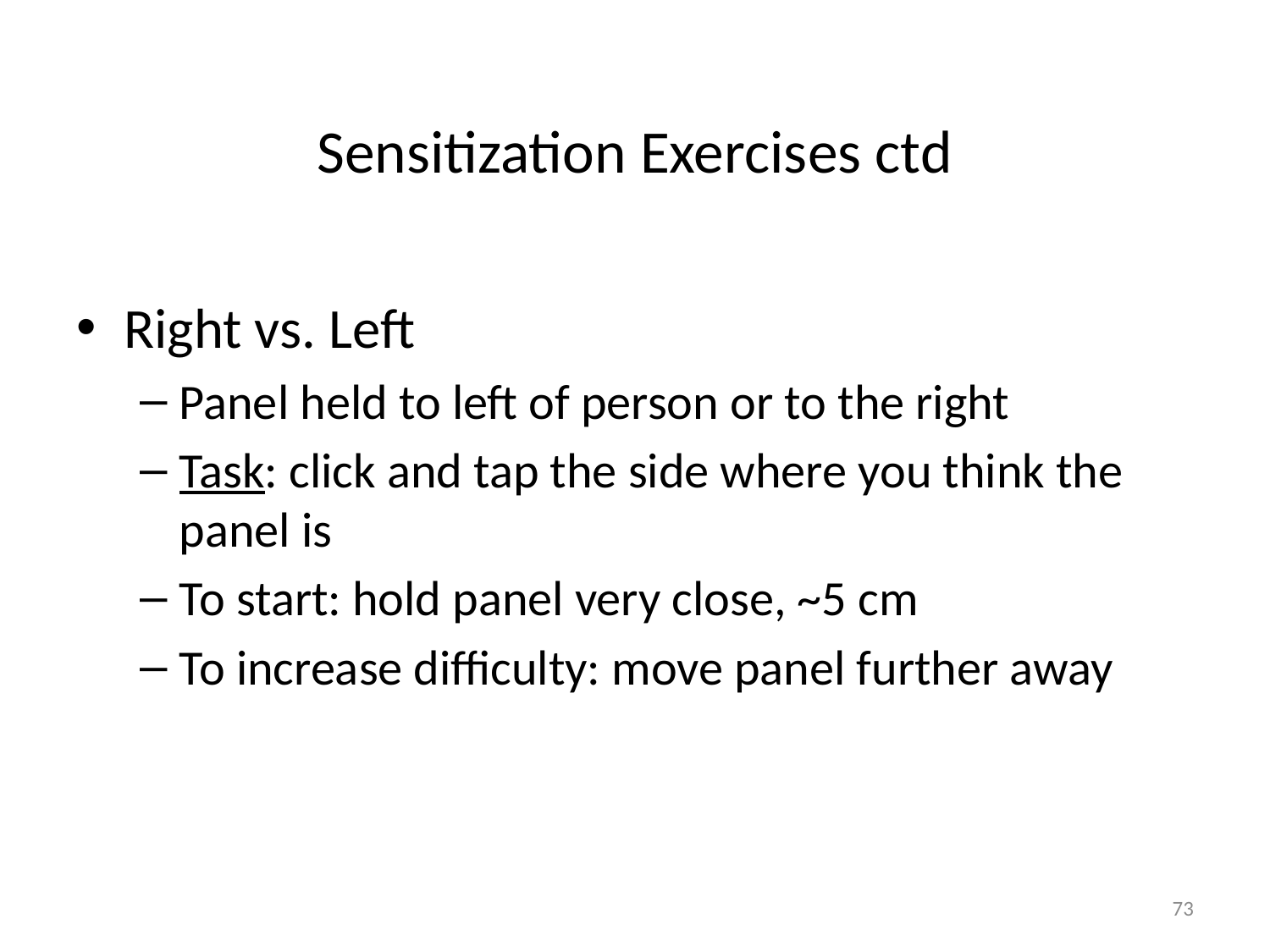

# Sensitization Exercises ctd
Right vs. Left
Panel held to left of person or to the right
Task: click and tap the side where you think the panel is
To start: hold panel very close, ~5 cm
To increase difficulty: move panel further away
73

## Slide 74
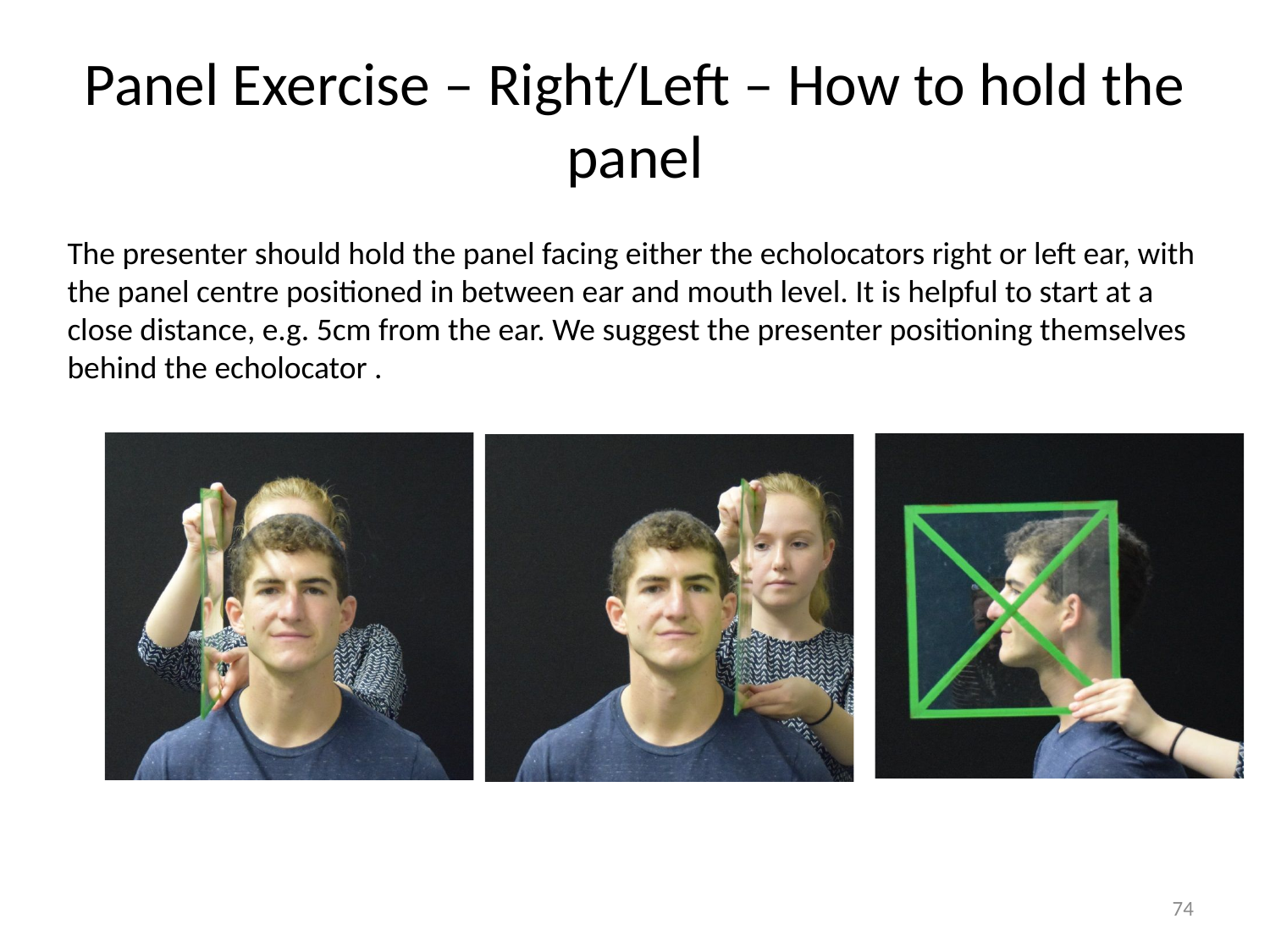

# Panel Exercise – Right/Left – How to hold the panel
The presenter should hold the panel facing either the echolocators right or left ear, with the panel centre positioned in between ear and mouth level. It is helpful to start at a close distance, e.g. 5cm from the ear. We suggest the presenter positioning themselves behind the echolocator .
74

## Slide 75
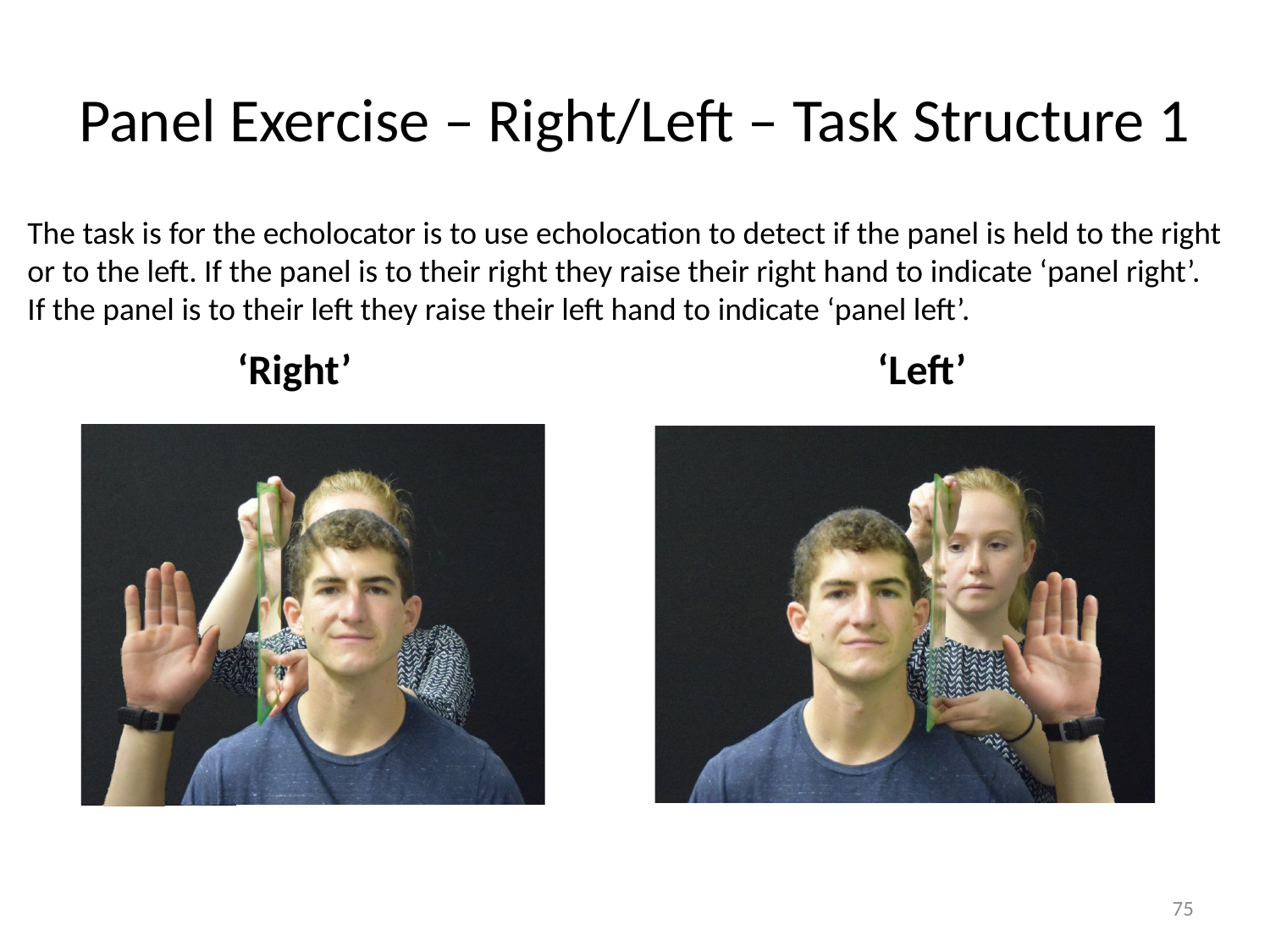

# Panel Exercise – Right/Left – Task Structure 1
The task is for the echolocator is to use echolocation to detect if the panel is held to the right or to the left. If the panel is to their right they raise their right hand to indicate ‘panel right’. If the panel is to their left they raise their left hand to indicate ‘panel left’.
‘Right’
‘Left’
75

## Slide 76
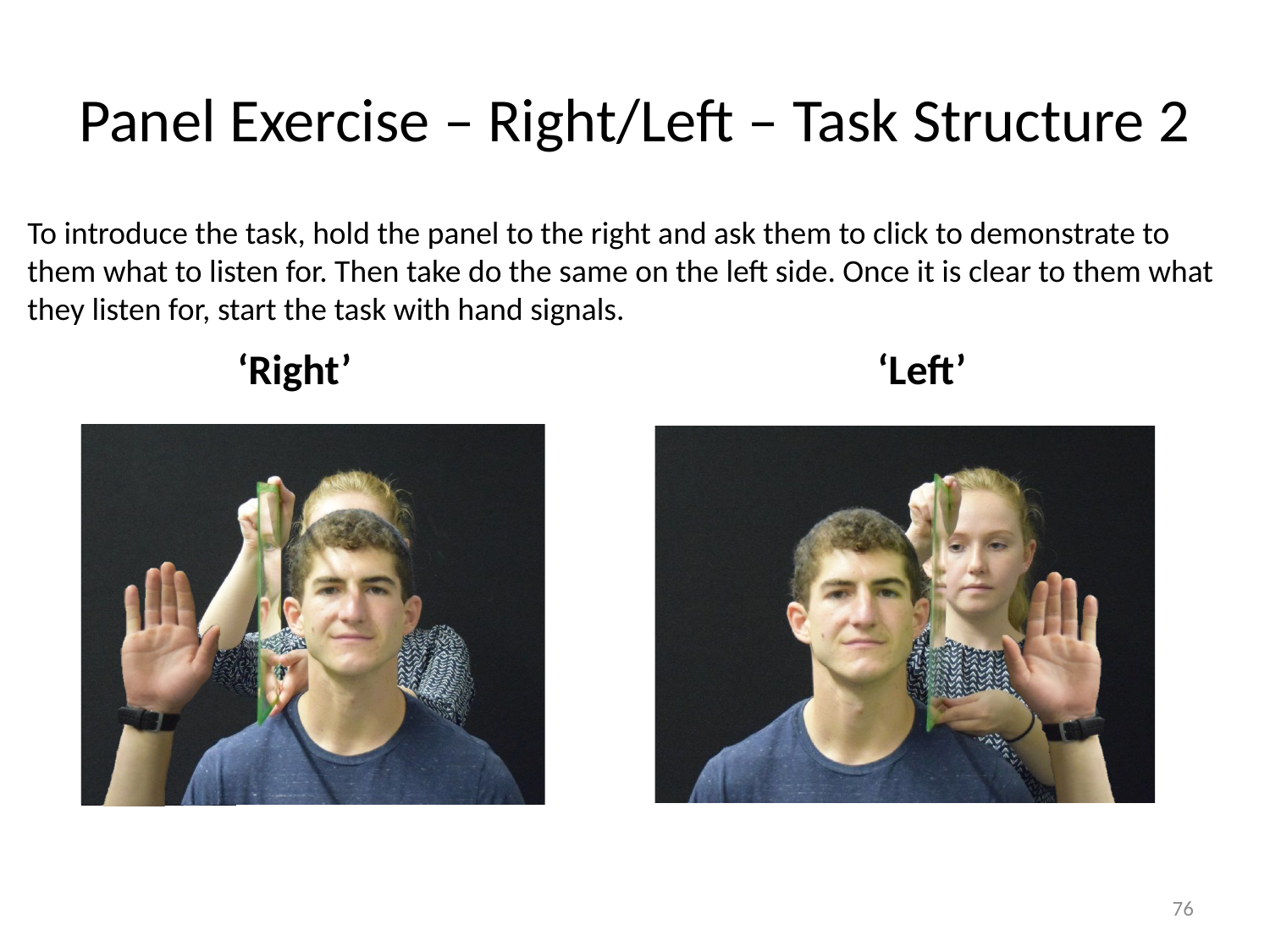

# Panel Exercise – Right/Left – Task Structure 2
To introduce the task, hold the panel to the right and ask them to click to demonstrate to them what to listen for. Then take do the same on the left side. Once it is clear to them what they listen for, start the task with hand signals.
‘Right’
‘Left’
76

## Slide 77
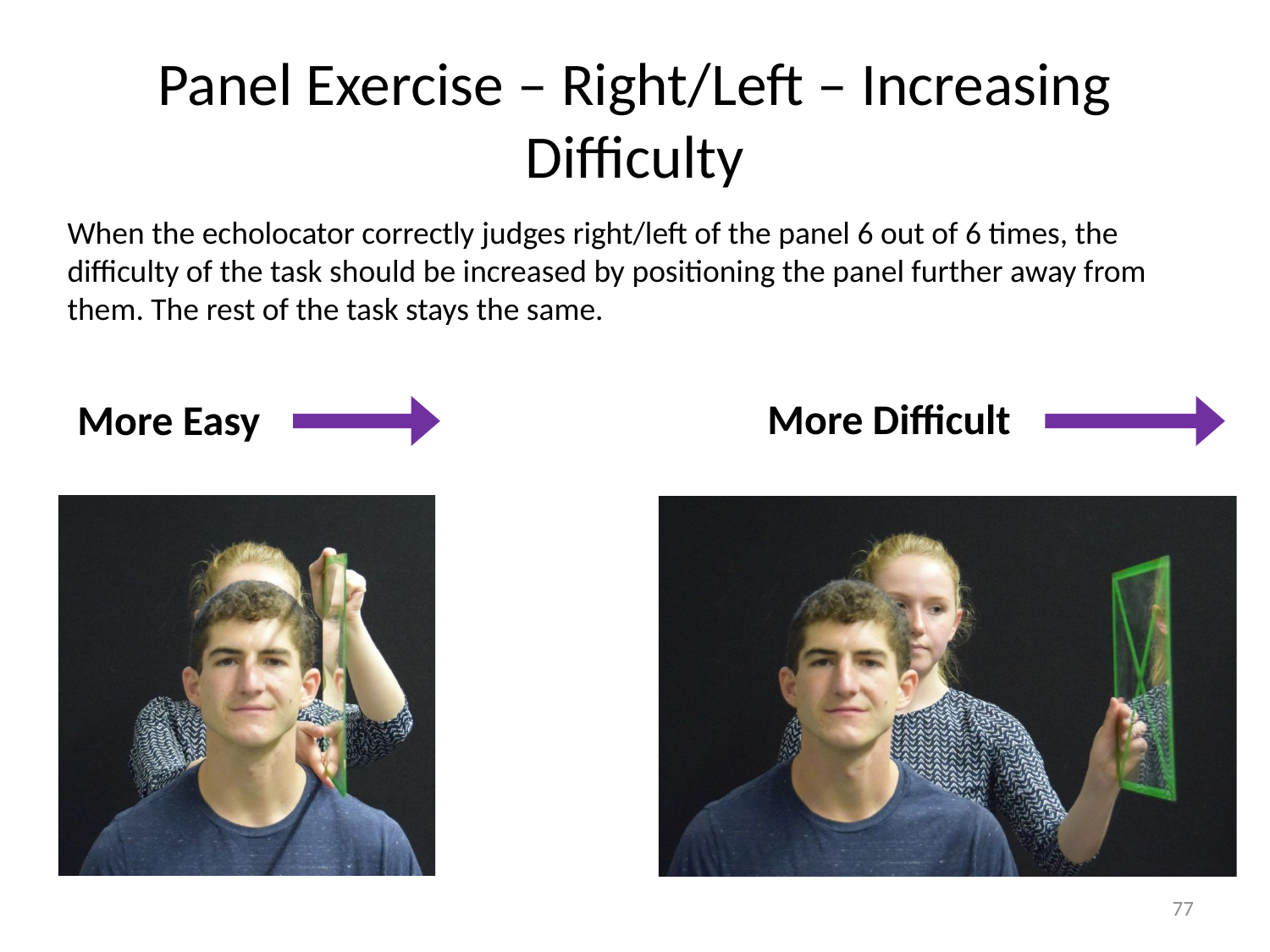

# Panel Exercise – Right/Left – Increasing Difficulty
When the echolocator correctly judges right/left of the panel 6 out of 6 times, the difficulty of the task should be increased by positioning the panel further away from them. The rest of the task stays the same.
More Difficult
More Easy
77

## Slide 78
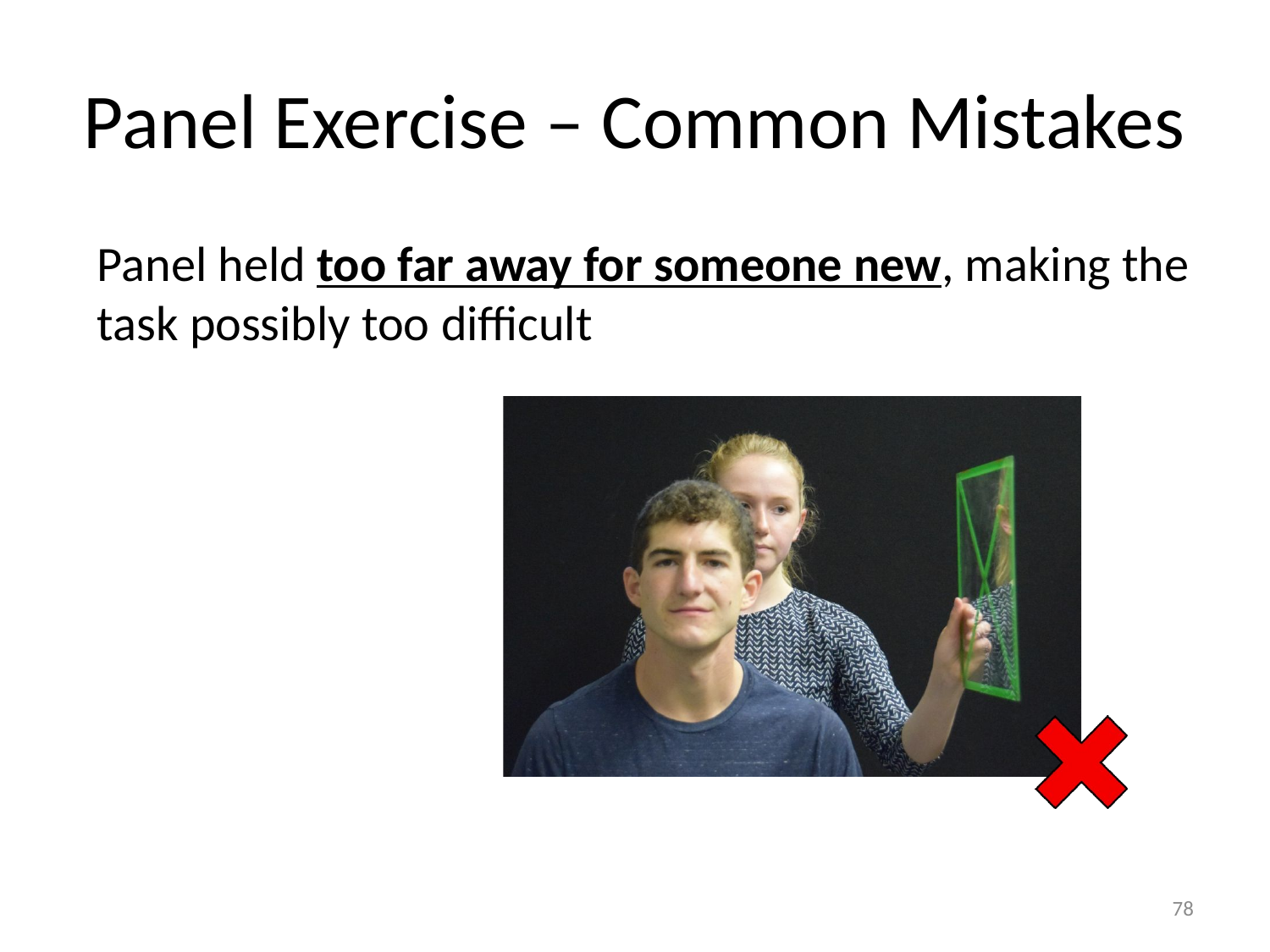

# Panel Exercise – Common Mistakes
Panel held too far away for someone new, making the task possibly too difficult
78

## Slide 79
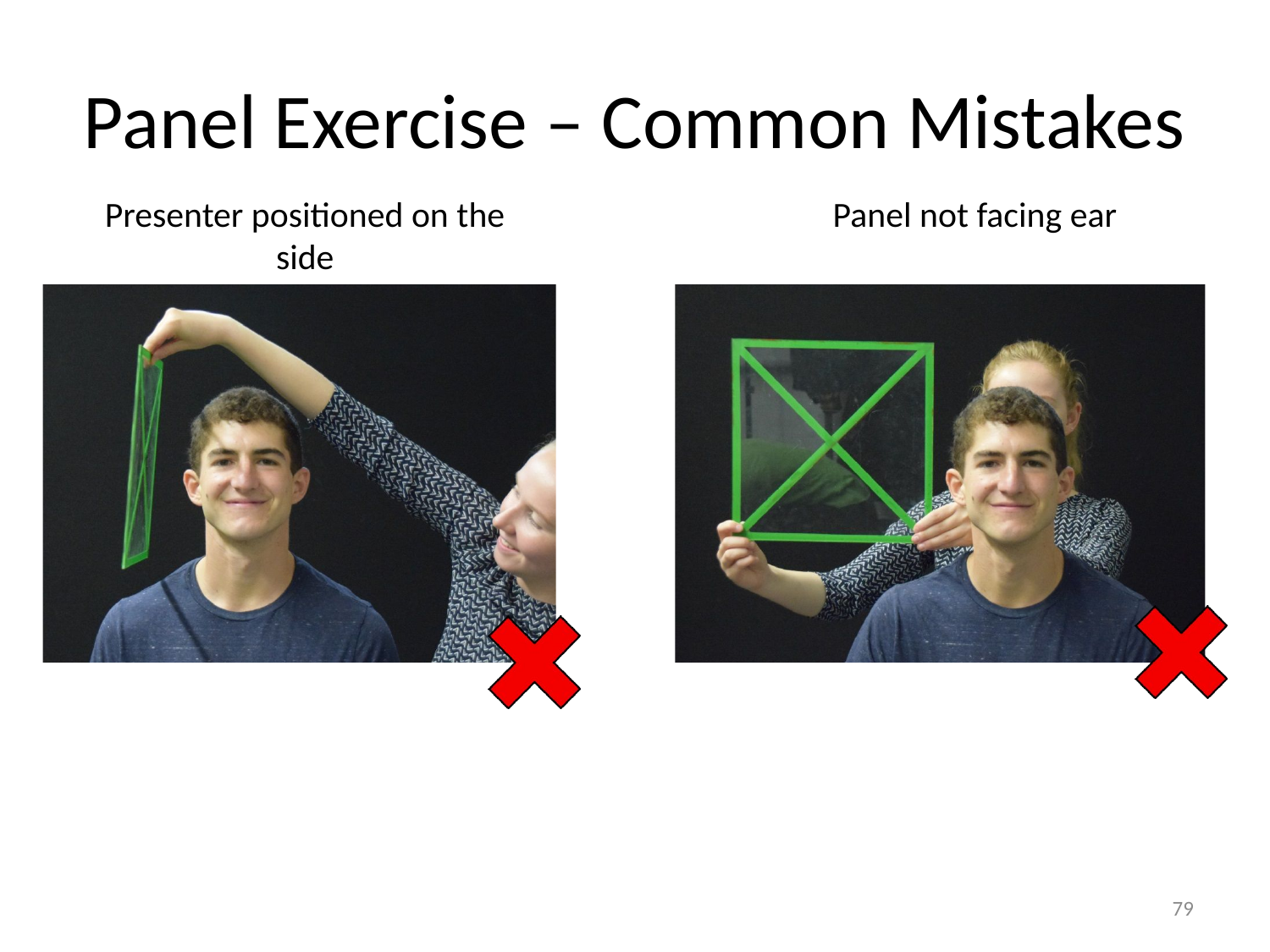

# Panel Exercise – Common Mistakes
Presenter positioned on the side
Panel not facing ear
79

## Slide 80
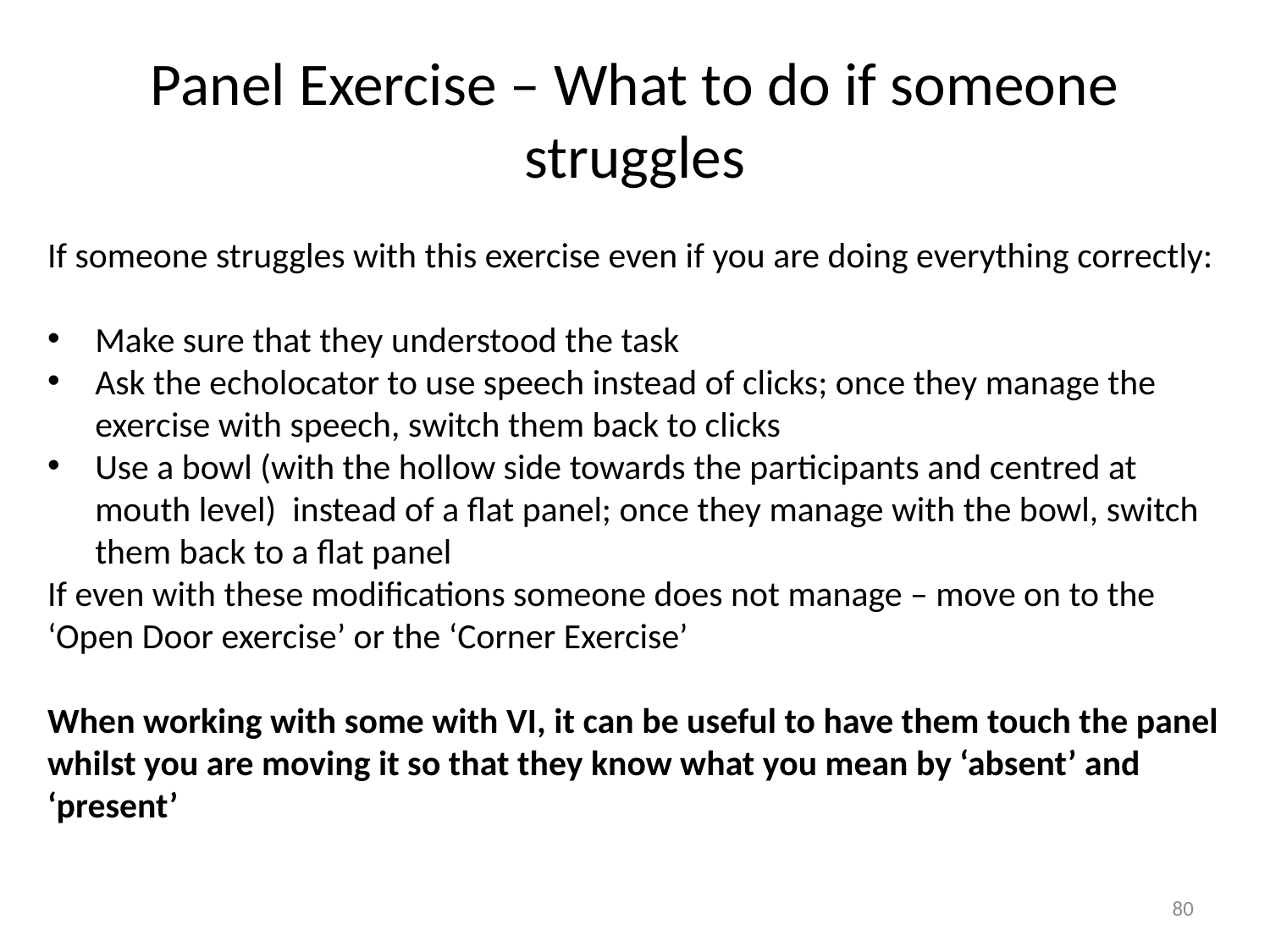

# Panel Exercise – What to do if someone struggles
If someone struggles with this exercise even if you are doing everything correctly:
Make sure that they understood the task
Ask the echolocator to use speech instead of clicks; once they manage the exercise with speech, switch them back to clicks
Use a bowl (with the hollow side towards the participants and centred at mouth level) instead of a flat panel; once they manage with the bowl, switch them back to a flat panel
If even with these modifications someone does not manage – move on to the ‘Open Door exercise’ or the ‘Corner Exercise’
When working with some with VI, it can be useful to have them touch the panel whilst you are moving it so that they know what you mean by ‘absent’ and ‘present’
80

## Slide 81
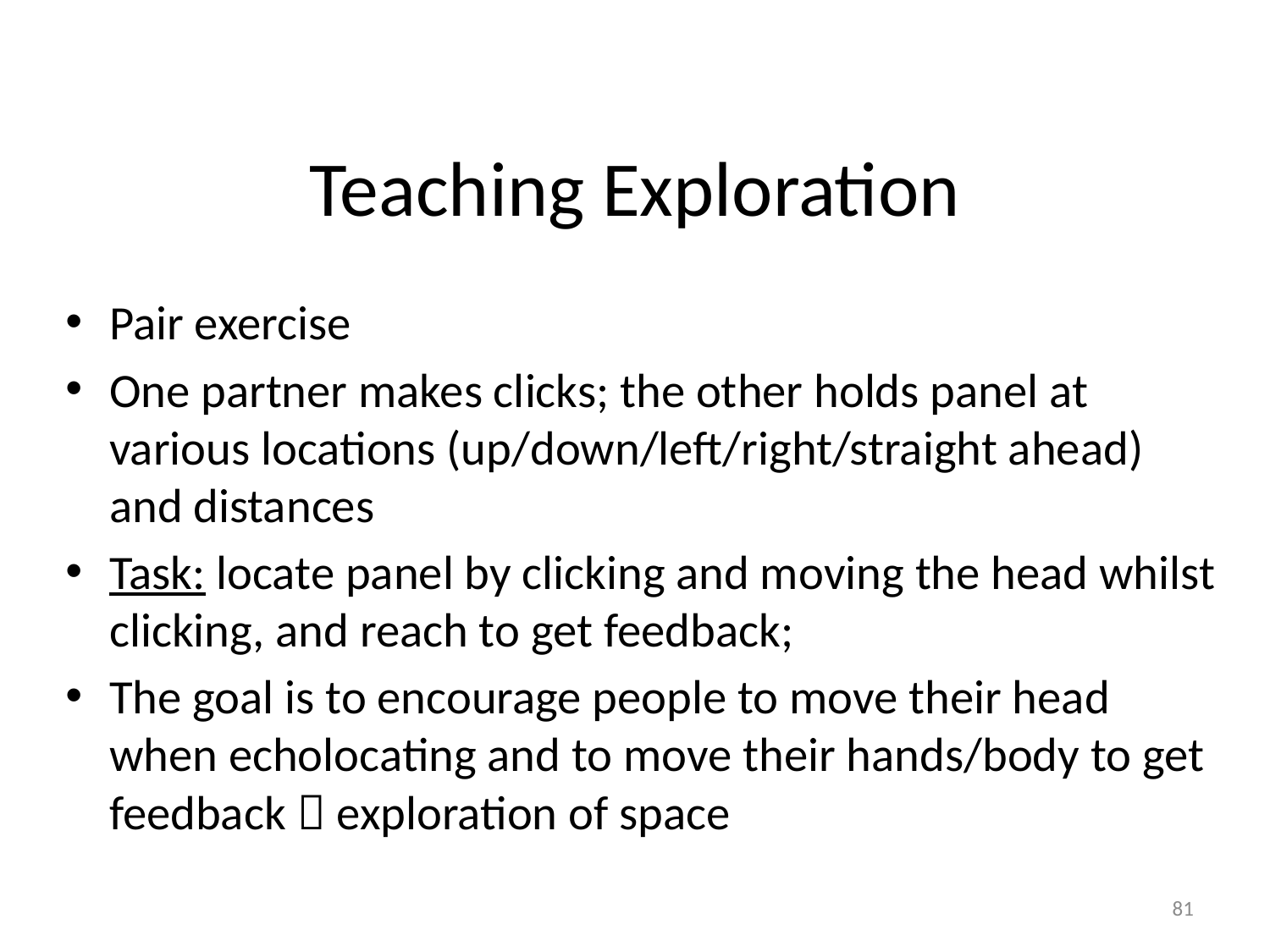

# Teaching Exploration
Pair exercise
One partner makes clicks; the other holds panel at various locations (up/down/left/right/straight ahead) and distances
Task: locate panel by clicking and moving the head whilst clicking, and reach to get feedback;
The goal is to encourage people to move their head when echolocating and to move their hands/body to get feedback  exploration of space
81

## Slide 82
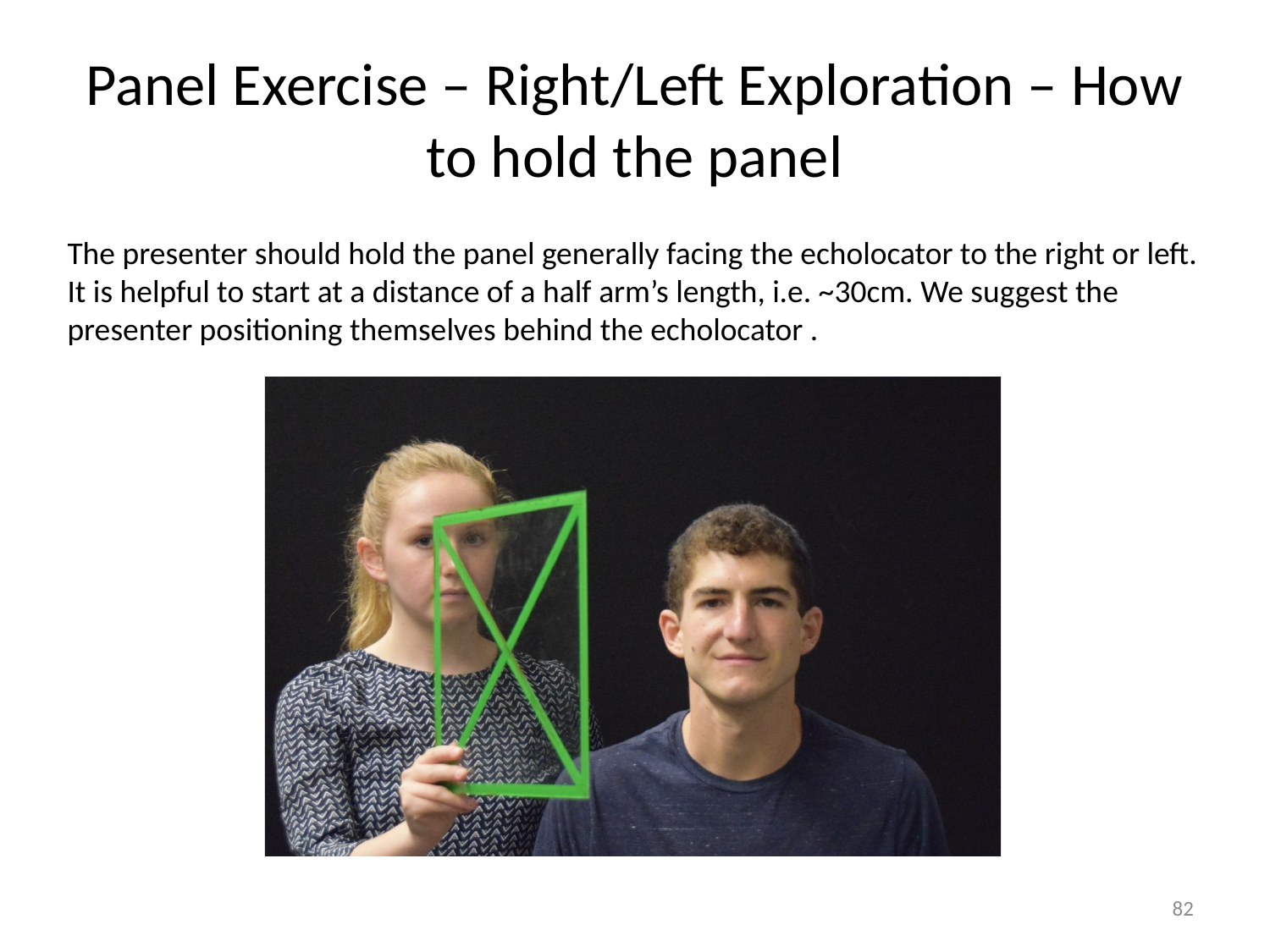

# Panel Exercise – Right/Left Exploration – How to hold the panel
The presenter should hold the panel generally facing the echolocator to the right or left. It is helpful to start at a distance of a half arm’s length, i.e. ~30cm. We suggest the presenter positioning themselves behind the echolocator .
82

## Slide 83
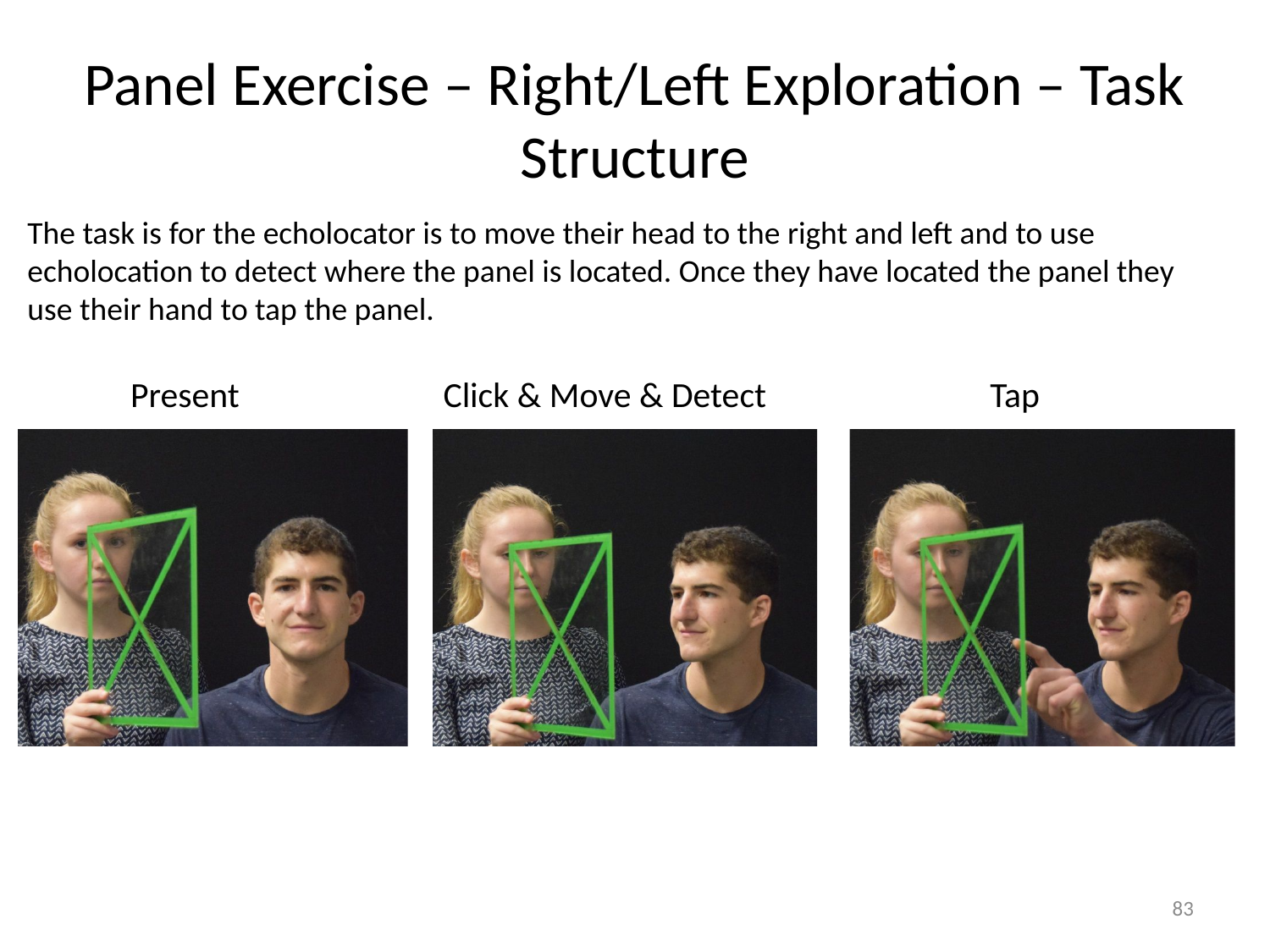

# Panel Exercise – Right/Left Exploration – Task Structure
The task is for the echolocator is to move their head to the right and left and to use echolocation to detect where the panel is located. Once they have located the panel they use their hand to tap the panel.
Present
Click & Move & Detect
Tap
83

## Slide 84
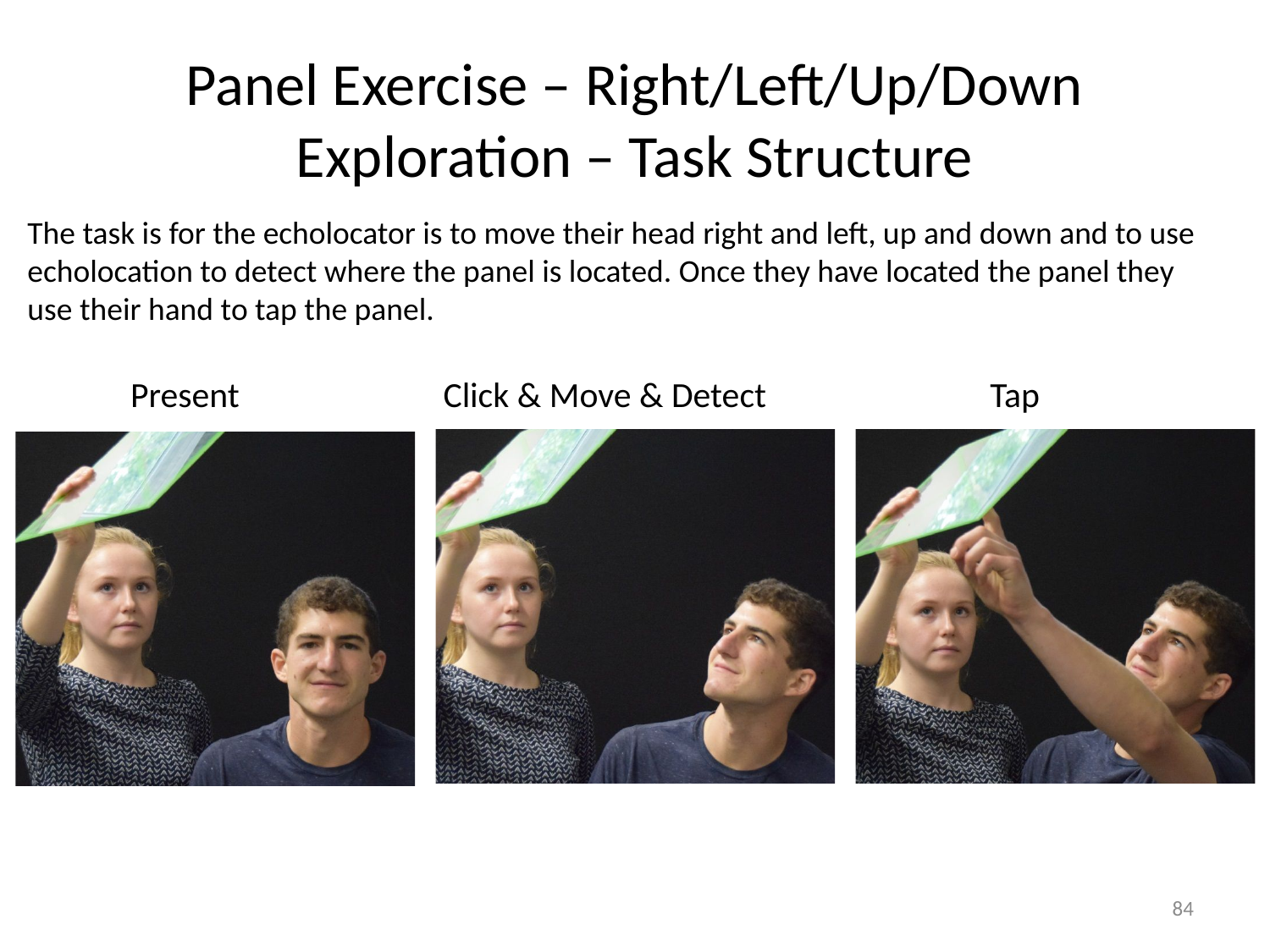

# Panel Exercise – Right/Left/Up/Down Exploration – Task Structure
The task is for the echolocator is to move their head right and left, up and down and to use echolocation to detect where the panel is located. Once they have located the panel they use their hand to tap the panel.
Present
Click & Move & Detect
Tap
84

## Slide 85
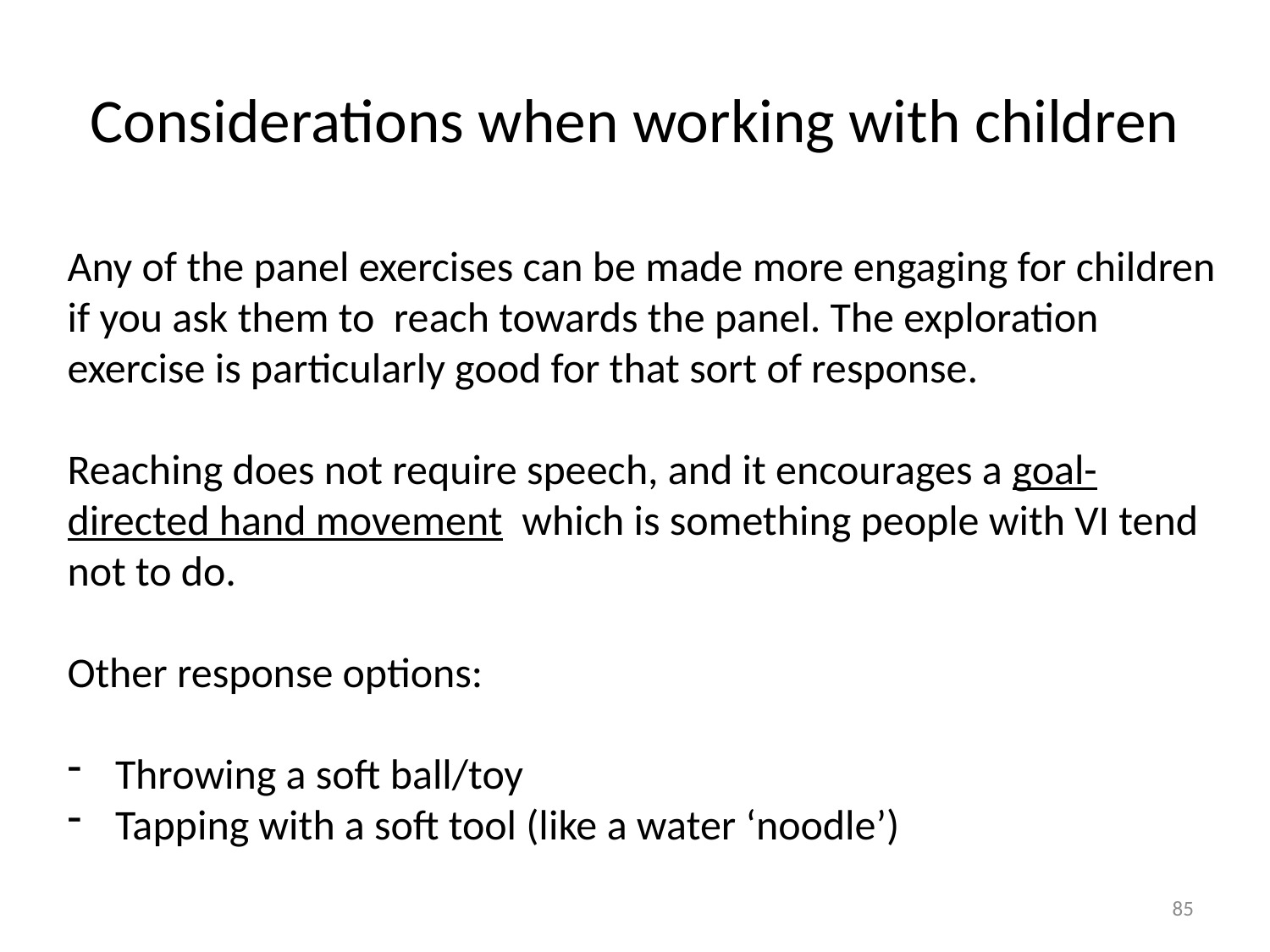

# Considerations when working with children
Any of the panel exercises can be made more engaging for children if you ask them to reach towards the panel. The exploration exercise is particularly good for that sort of response.
Reaching does not require speech, and it encourages a goal-directed hand movement which is something people with VI tend not to do.
Other response options:
Throwing a soft ball/toy
Tapping with a soft tool (like a water ‘noodle’)
85

## Slide 86
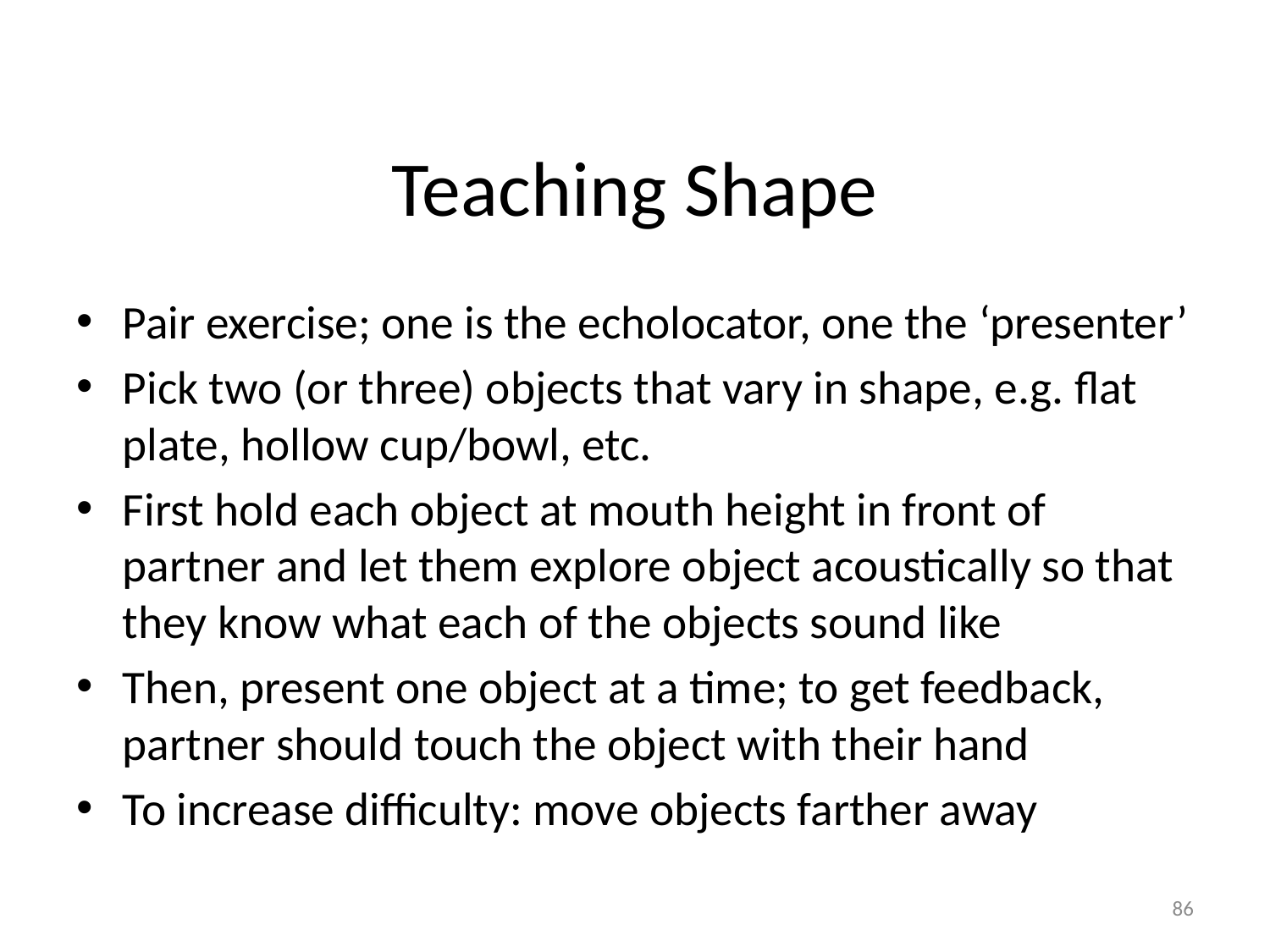

# Teaching Shape
Pair exercise; one is the echolocator, one the ‘presenter’
Pick two (or three) objects that vary in shape, e.g. flat plate, hollow cup/bowl, etc.
First hold each object at mouth height in front of partner and let them explore object acoustically so that they know what each of the objects sound like
Then, present one object at a time; to get feedback, partner should touch the object with their hand
To increase difficulty: move objects farther away
86

## Slide 87
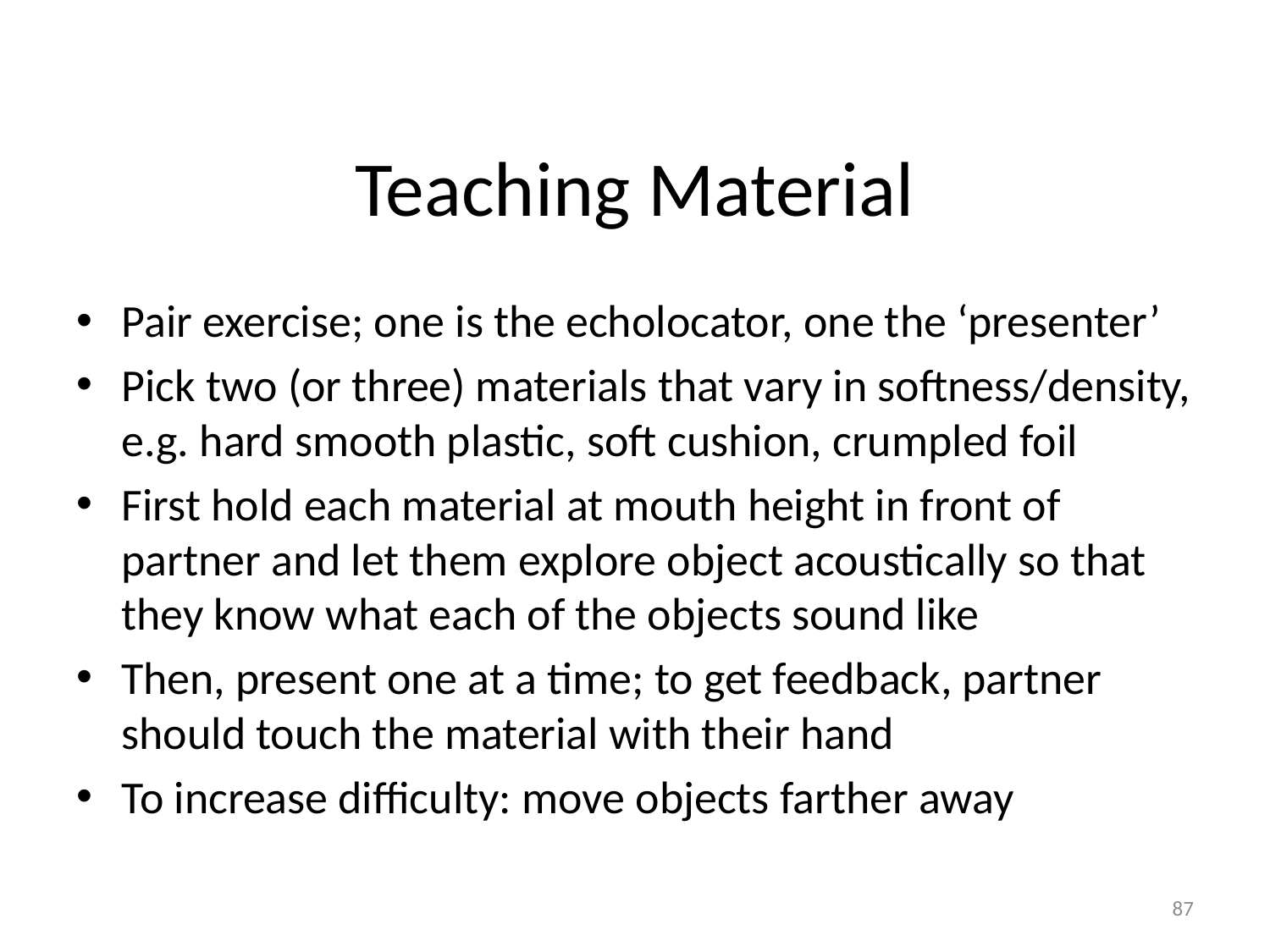

# Teaching Material
Pair exercise; one is the echolocator, one the ‘presenter’
Pick two (or three) materials that vary in softness/density, e.g. hard smooth plastic, soft cushion, crumpled foil
First hold each material at mouth height in front of partner and let them explore object acoustically so that they know what each of the objects sound like
Then, present one at a time; to get feedback, partner should touch the material with their hand
To increase difficulty: move objects farther away
87

## Slide 88
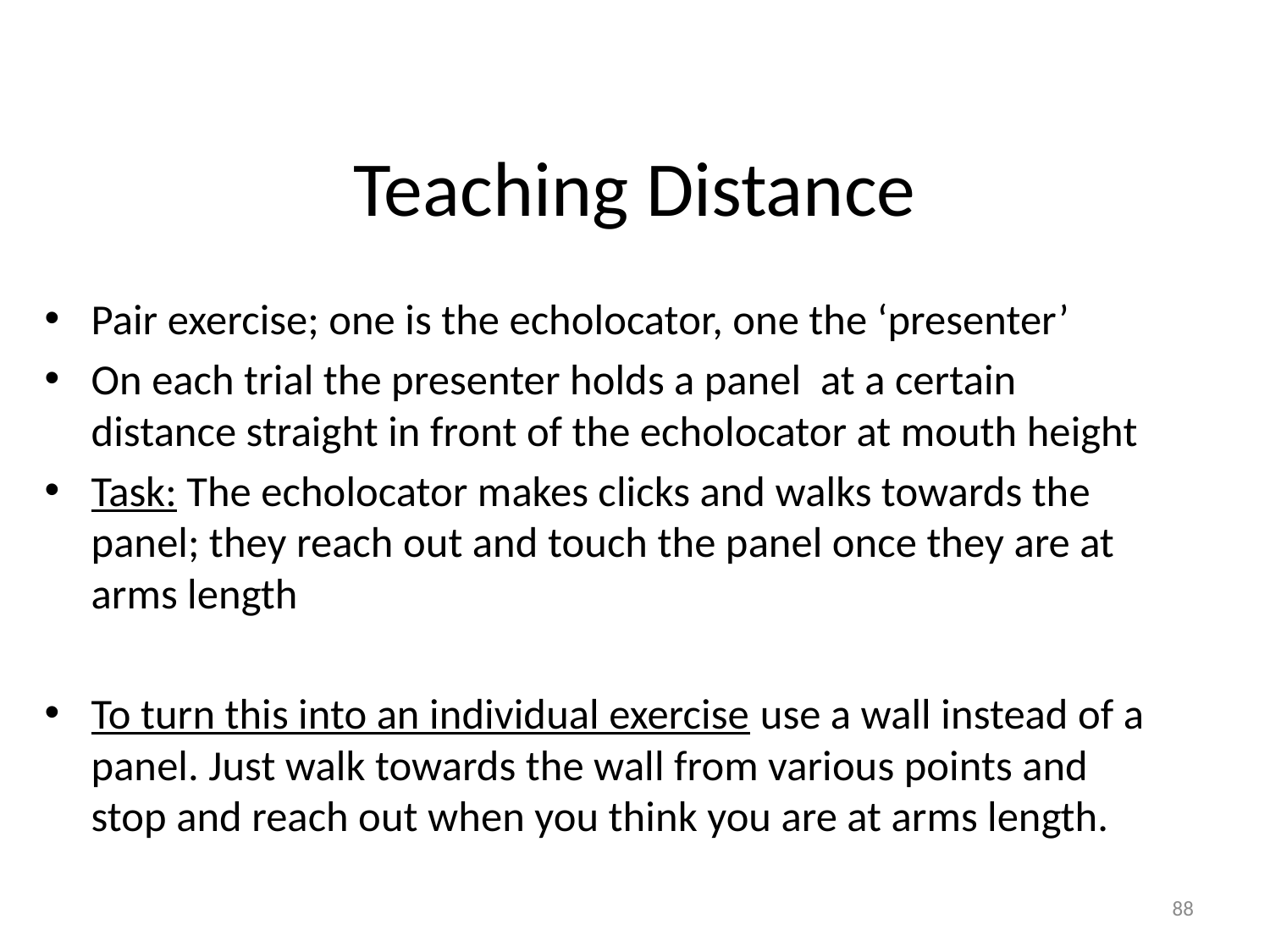

# Teaching Distance
Pair exercise; one is the echolocator, one the ‘presenter’
On each trial the presenter holds a panel at a certain distance straight in front of the echolocator at mouth height
Task: The echolocator makes clicks and walks towards the panel; they reach out and touch the panel once they are at arms length
To turn this into an individual exercise use a wall instead of a panel. Just walk towards the wall from various points and stop and reach out when you think you are at arms length.
88

## Slide 89
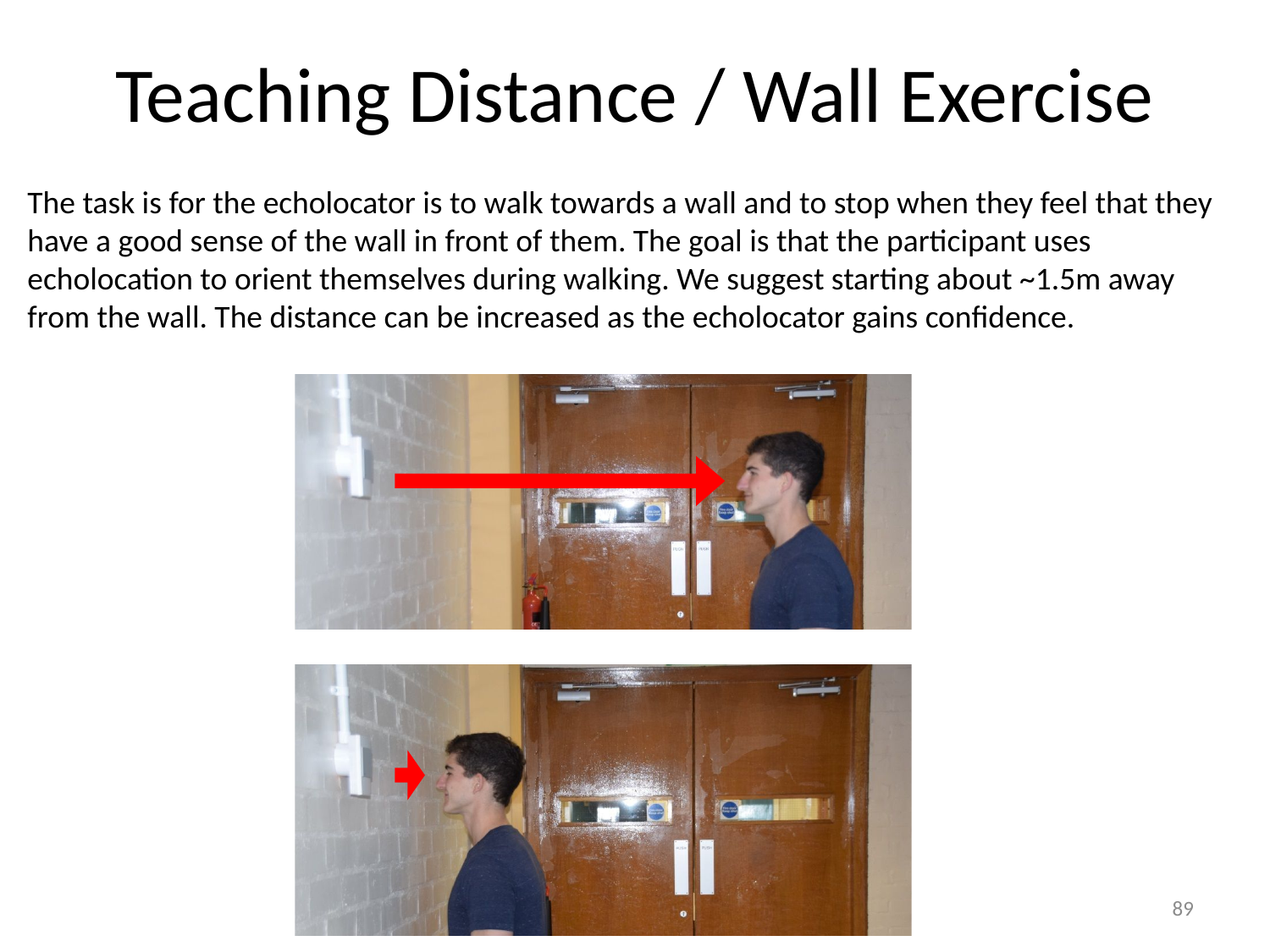

Teaching Distance / Wall Exercise
The task is for the echolocator is to walk towards a wall and to stop when they feel that they have a good sense of the wall in front of them. The goal is that the participant uses echolocation to orient themselves during walking. We suggest starting about ~1.5m away from the wall. The distance can be increased as the echolocator gains confidence.
89

## Slide 90
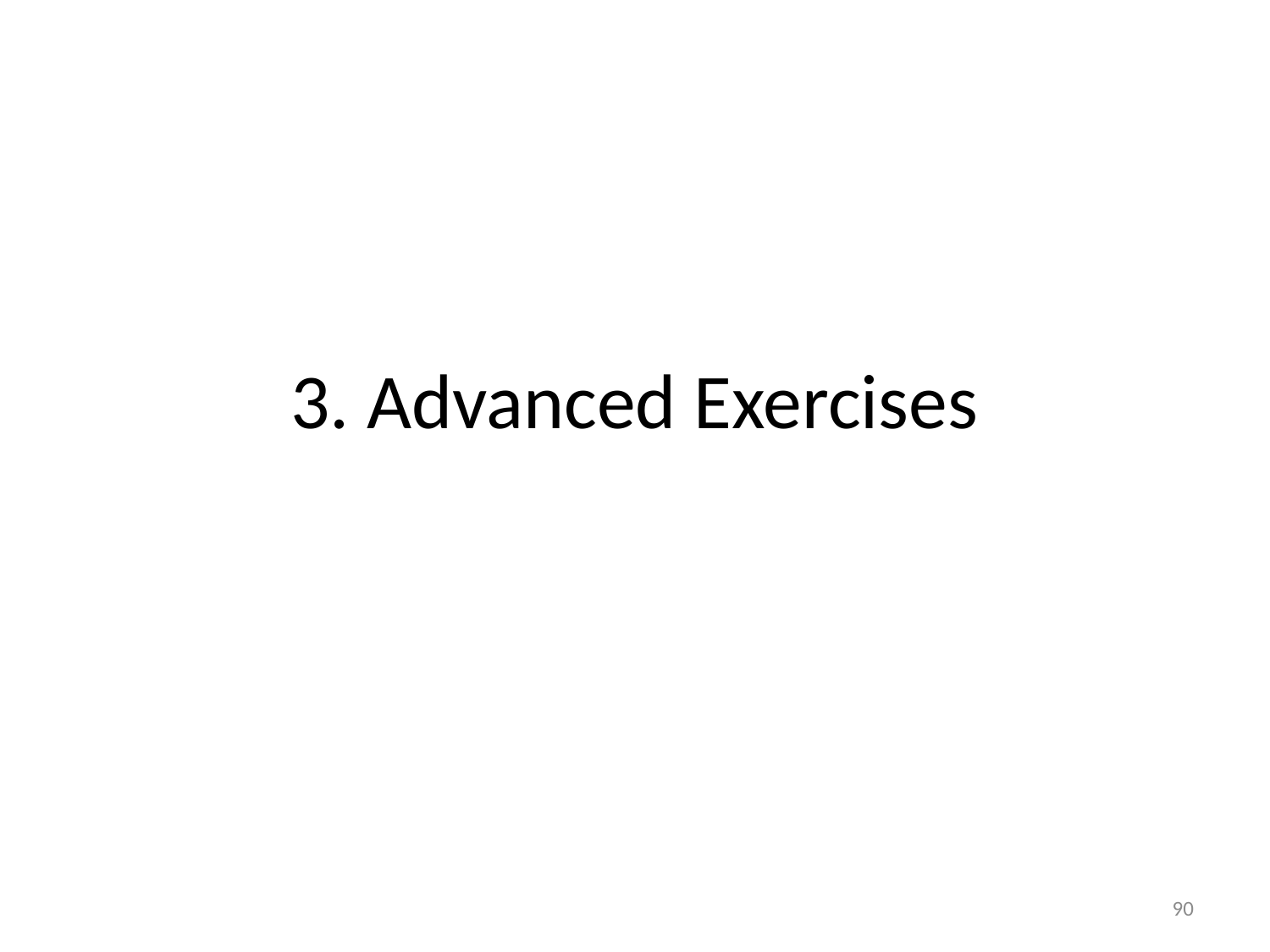

# 3. Advanced Exercises
90

## Slide 91
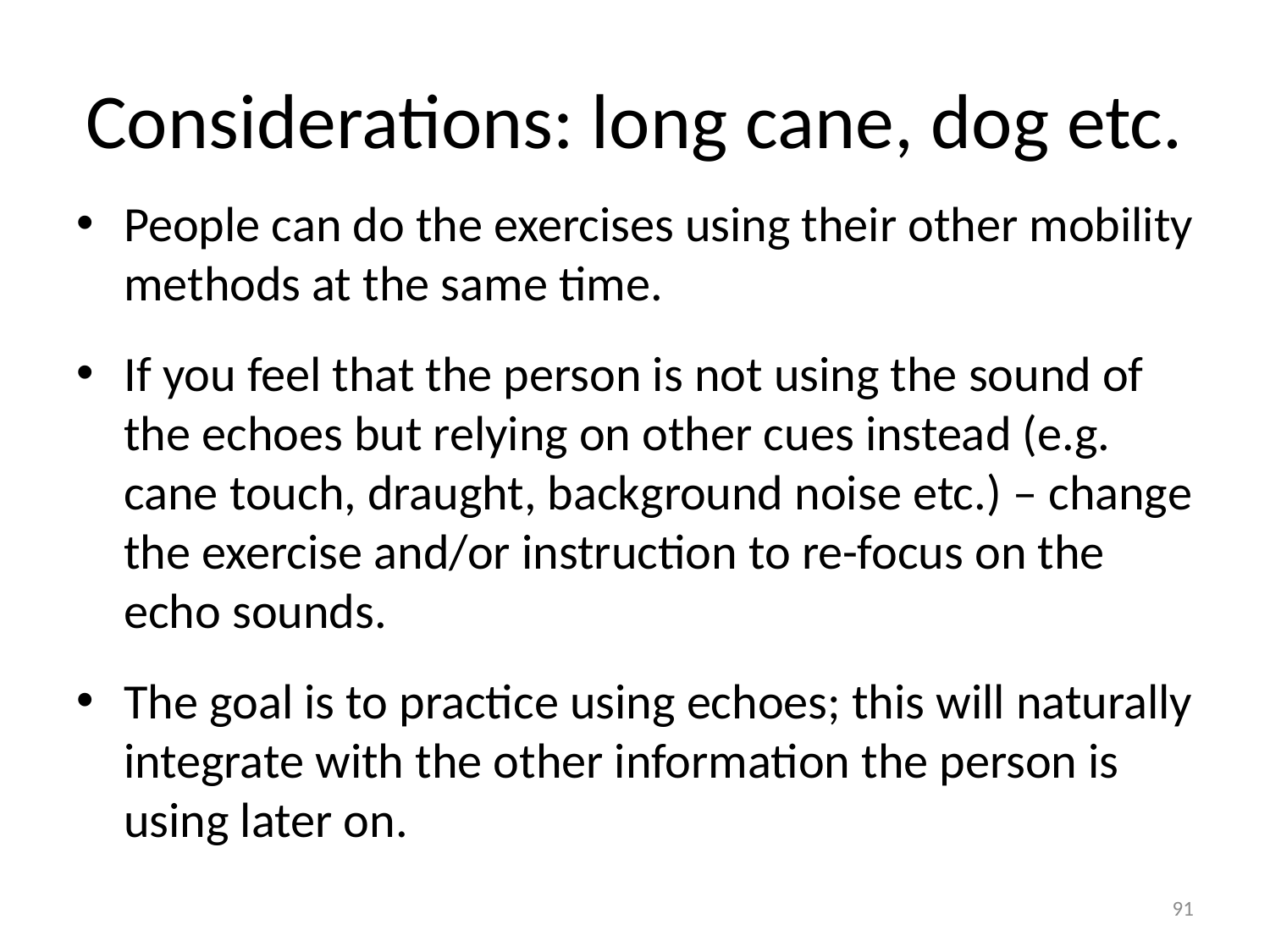

# Considerations: long cane, dog etc.
People can do the exercises using their other mobility methods at the same time.
If you feel that the person is not using the sound of the echoes but relying on other cues instead (e.g. cane touch, draught, background noise etc.) – change the exercise and/or instruction to re-focus on the echo sounds.
The goal is to practice using echoes; this will naturally integrate with the other information the person is using later on.
91

## Slide 92
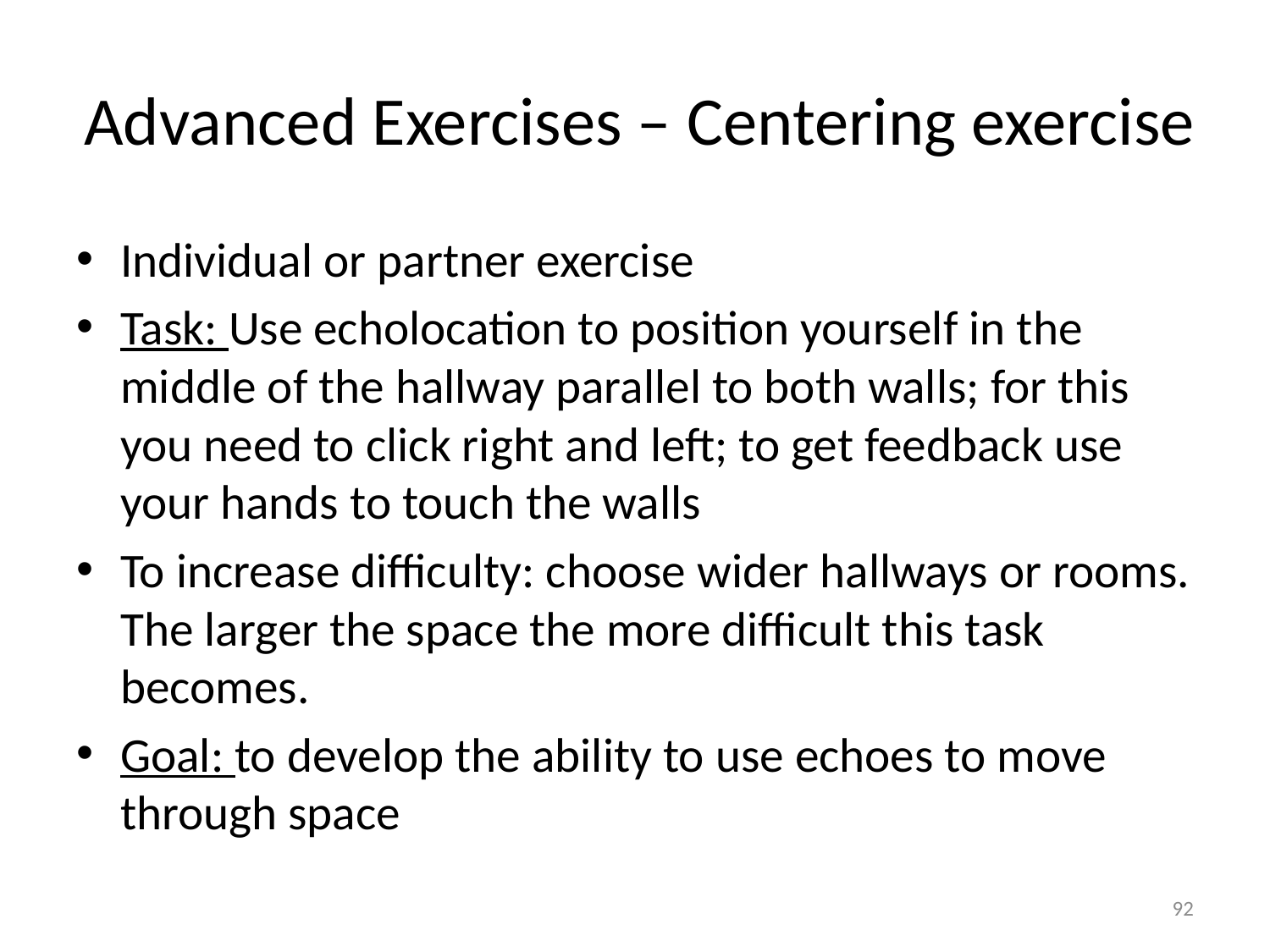

# Advanced Exercises – Centering exercise
Individual or partner exercise
Task: Use echolocation to position yourself in the middle of the hallway parallel to both walls; for this you need to click right and left; to get feedback use your hands to touch the walls
To increase difficulty: choose wider hallways or rooms. The larger the space the more difficult this task becomes.
Goal: to develop the ability to use echoes to move through space
92

## Slide 93
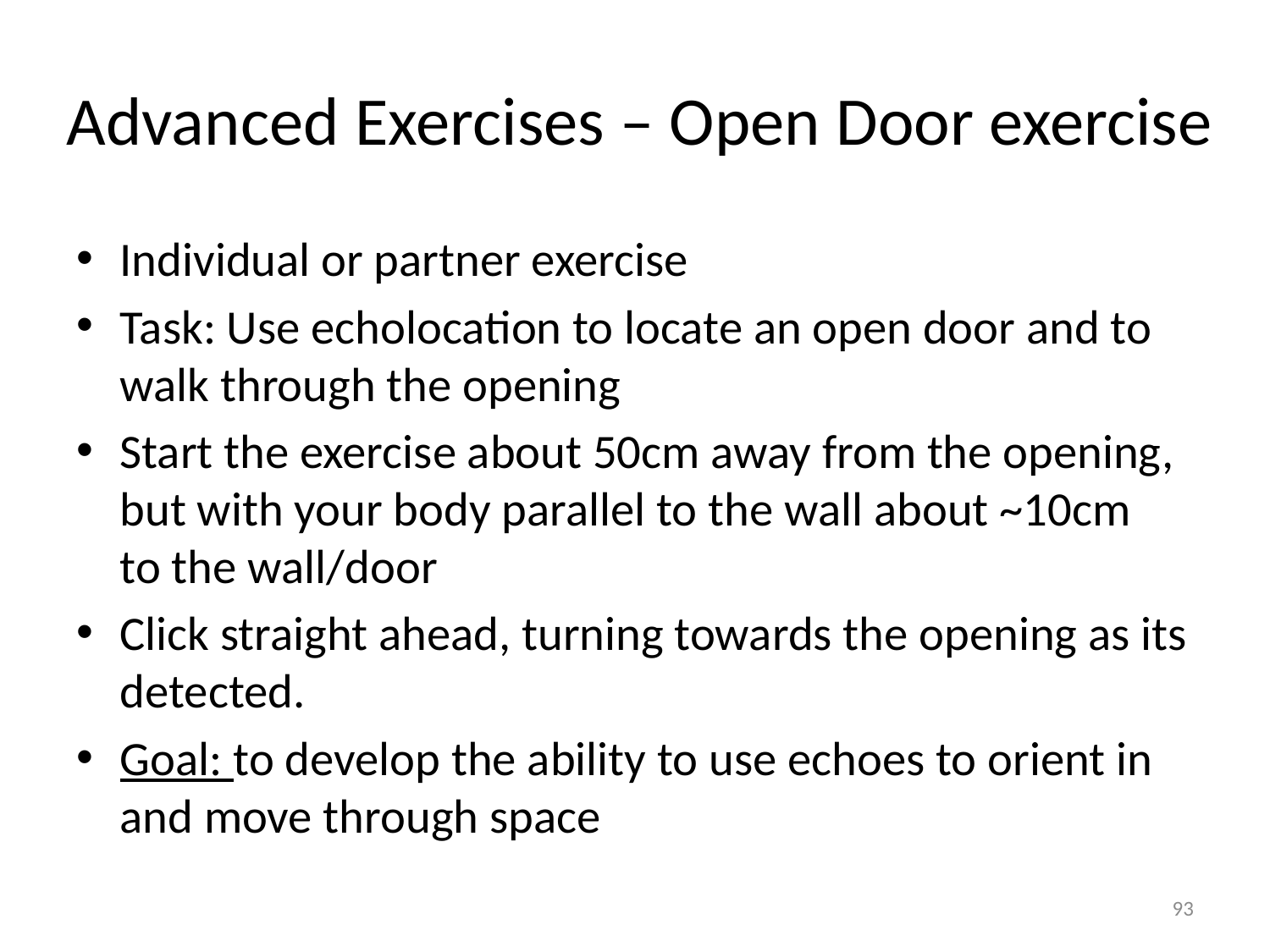

# Advanced Exercises – Open Door exercise
Individual or partner exercise
Task: Use echolocation to locate an open door and to walk through the opening
Start the exercise about 50cm away from the opening, but with your body parallel to the wall about ~10cm to the wall/door
Click straight ahead, turning towards the opening as its detected.
Goal: to develop the ability to use echoes to orient in and move through space
93

## Slide 94
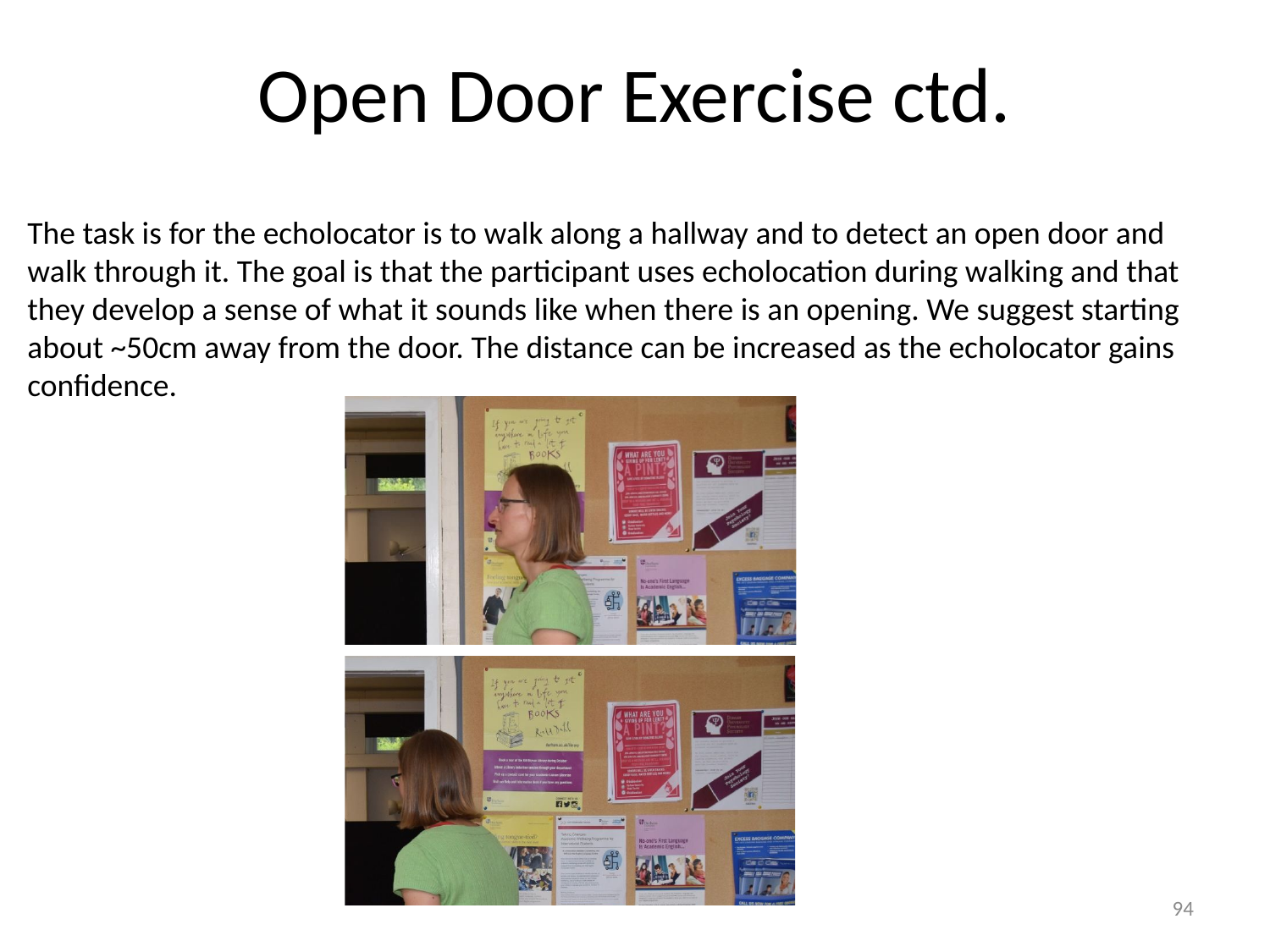

Open Door Exercise ctd.
The task is for the echolocator is to walk along a hallway and to detect an open door and walk through it. The goal is that the participant uses echolocation during walking and that they develop a sense of what it sounds like when there is an opening. We suggest starting about ~50cm away from the door. The distance can be increased as the echolocator gains confidence.
94

## Slide 95
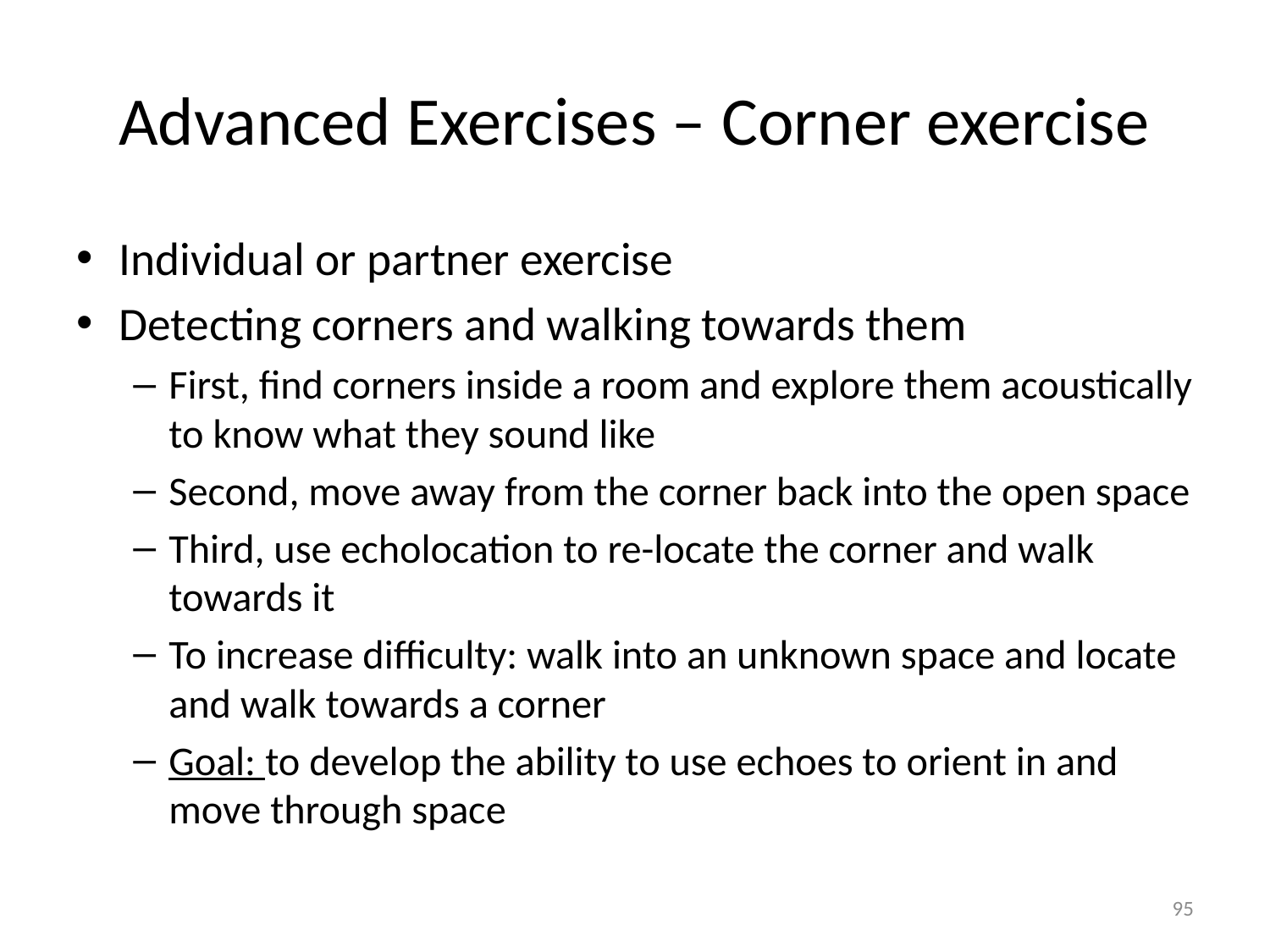

# Advanced Exercises – Corner exercise
Individual or partner exercise
Detecting corners and walking towards them
First, find corners inside a room and explore them acoustically to know what they sound like
Second, move away from the corner back into the open space
Third, use echolocation to re-locate the corner and walk towards it
To increase difficulty: walk into an unknown space and locate and walk towards a corner
Goal: to develop the ability to use echoes to orient in and move through space
95

## Slide 96
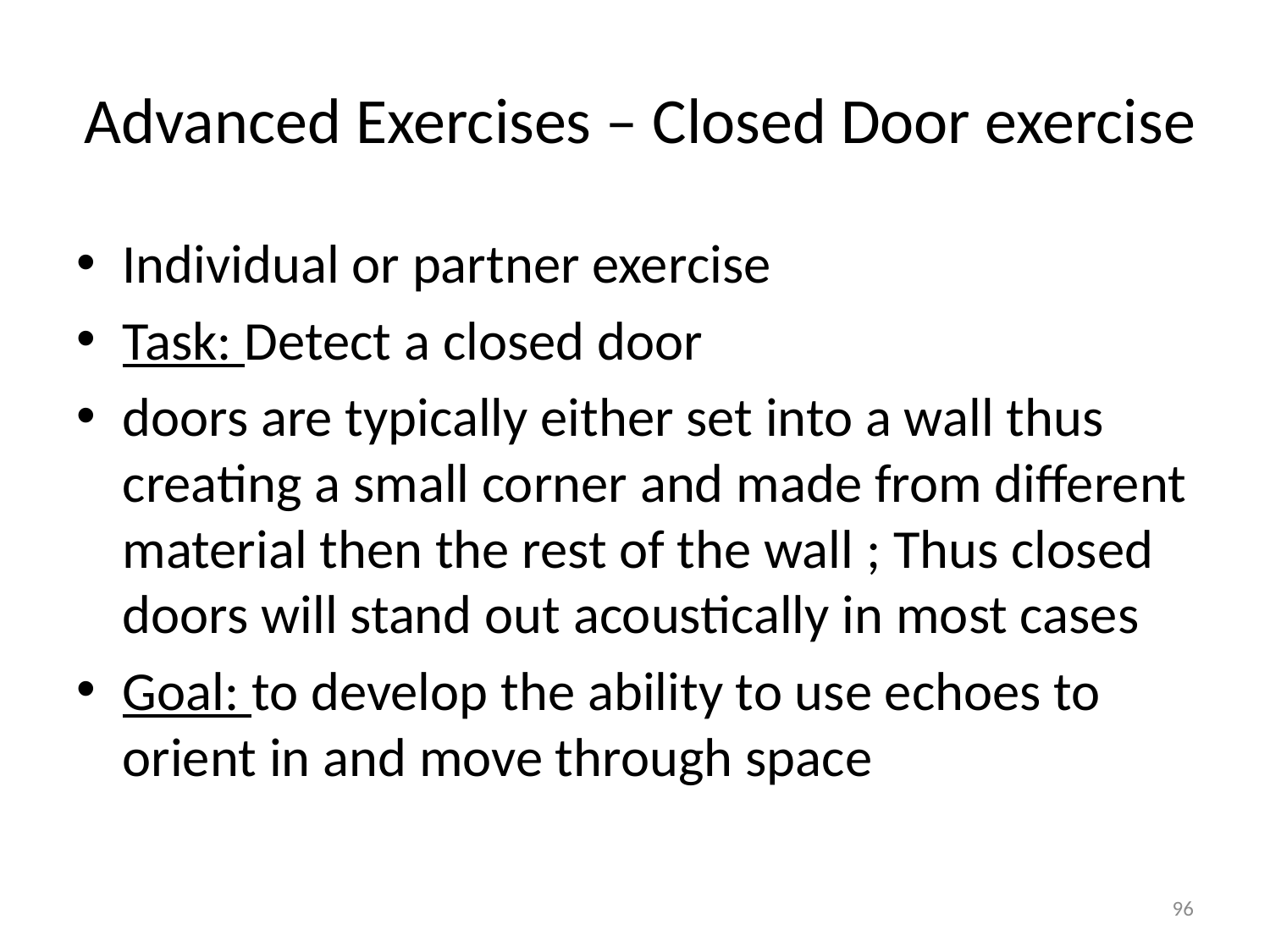

# Advanced Exercises – Closed Door exercise
Individual or partner exercise
Task: Detect a closed door
doors are typically either set into a wall thus creating a small corner and made from different material then the rest of the wall ; Thus closed doors will stand out acoustically in most cases
Goal: to develop the ability to use echoes to orient in and move through space
96

## Slide 97
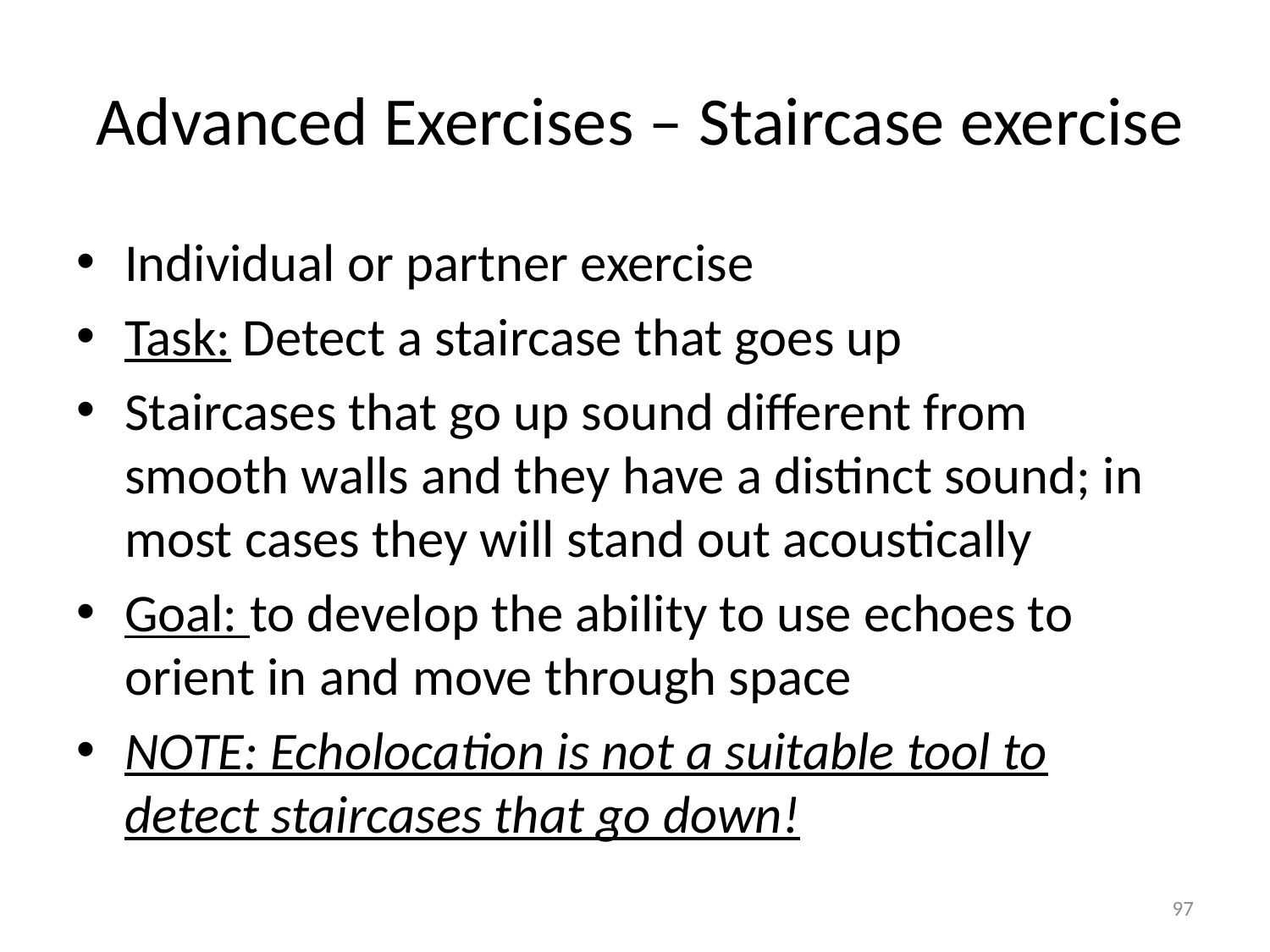

# Advanced Exercises – Staircase exercise
Individual or partner exercise
Task: Detect a staircase that goes up
Staircases that go up sound different from smooth walls and they have a distinct sound; in most cases they will stand out acoustically
Goal: to develop the ability to use echoes to orient in and move through space
NOTE: Echolocation is not a suitable tool to detect staircases that go down!
97

## Slide 98
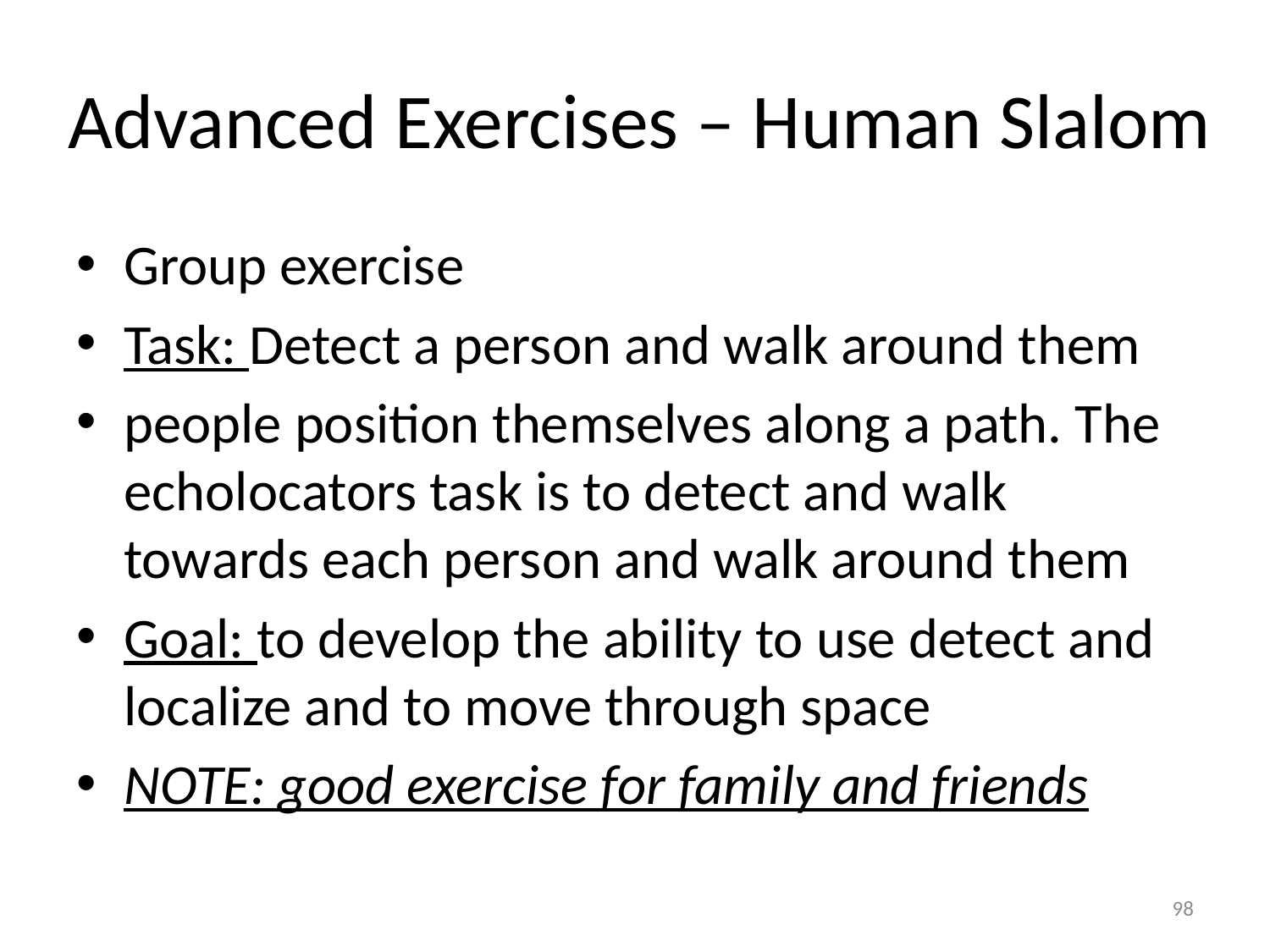

# Advanced Exercises – Human Slalom
Group exercise
Task: Detect a person and walk around them
people position themselves along a path. The echolocators task is to detect and walk towards each person and walk around them
Goal: to develop the ability to use detect and localize and to move through space
NOTE: good exercise for family and friends
98

## Slide 99
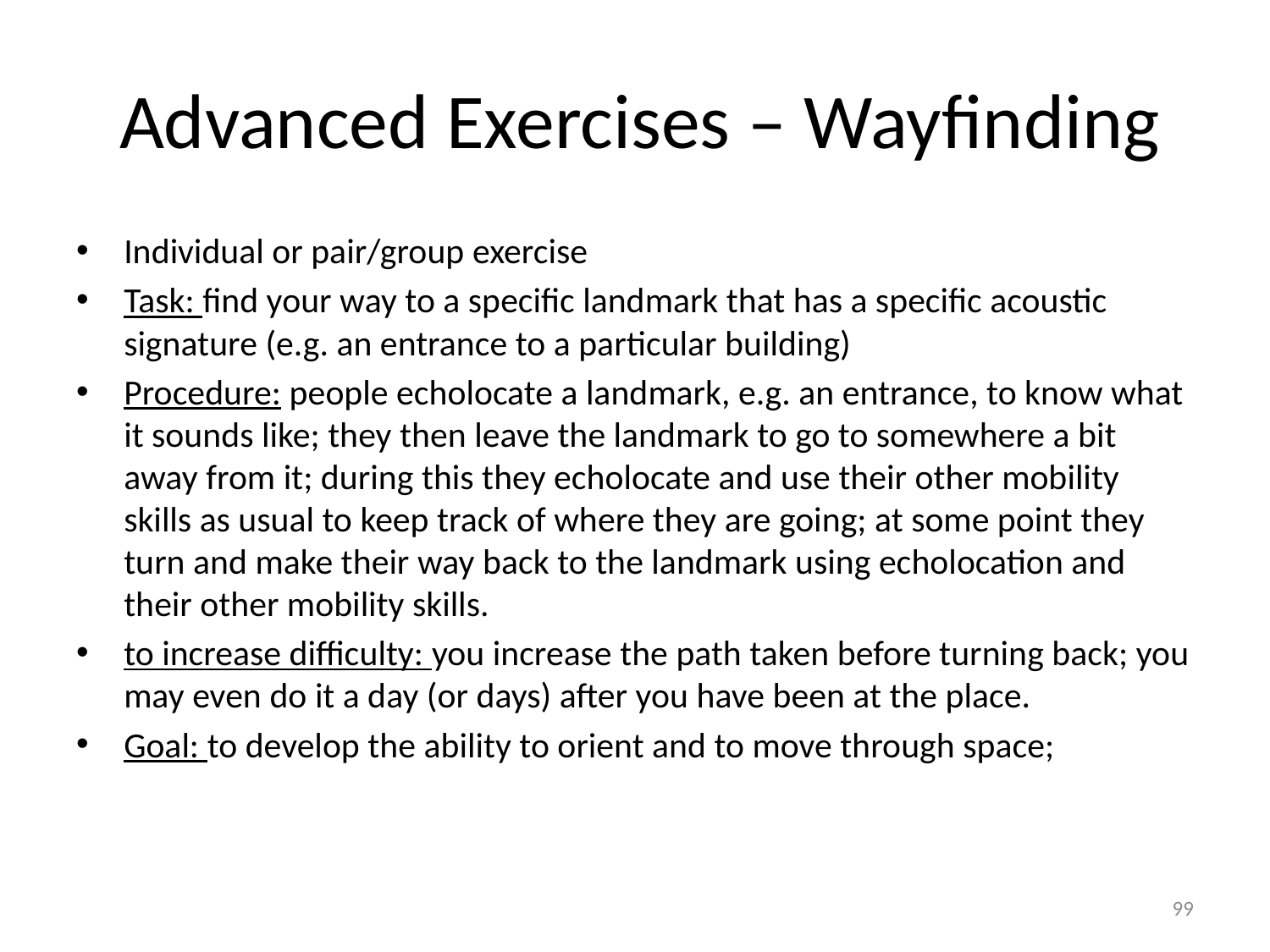

# Advanced Exercises – Wayfinding
Individual or pair/group exercise
Task: find your way to a specific landmark that has a specific acoustic signature (e.g. an entrance to a particular building)
Procedure: people echolocate a landmark, e.g. an entrance, to know what it sounds like; they then leave the landmark to go to somewhere a bit away from it; during this they echolocate and use their other mobility skills as usual to keep track of where they are going; at some point they turn and make their way back to the landmark using echolocation and their other mobility skills.
to increase difficulty: you increase the path taken before turning back; you may even do it a day (or days) after you have been at the place.
Goal: to develop the ability to orient and to move through space;
99

## Slide 100
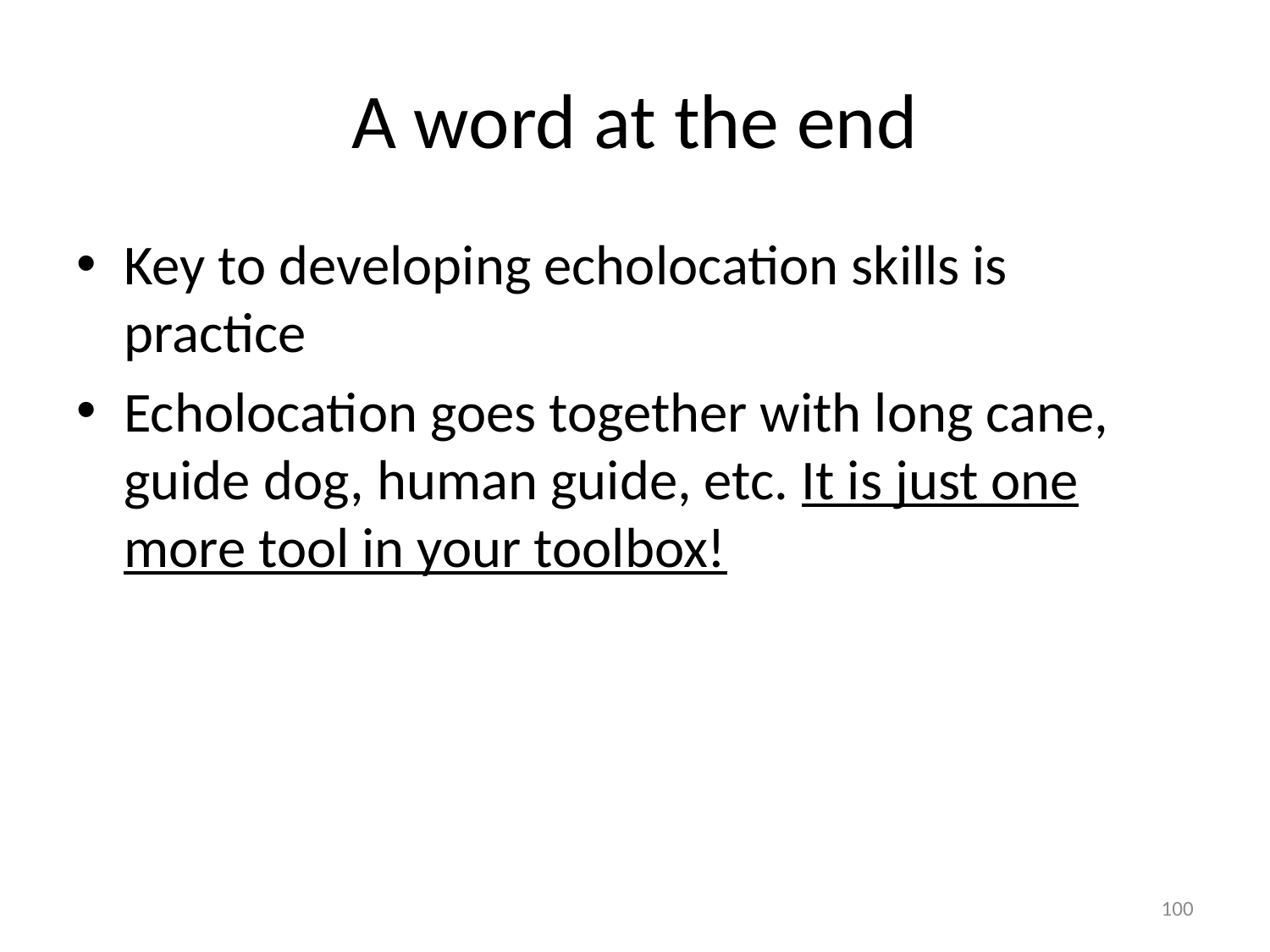

# A word at the end
Key to developing echolocation skills is practice
Echolocation goes together with long cane, guide dog, human guide, etc. It is just one more tool in your toolbox!
100

## Slide 101
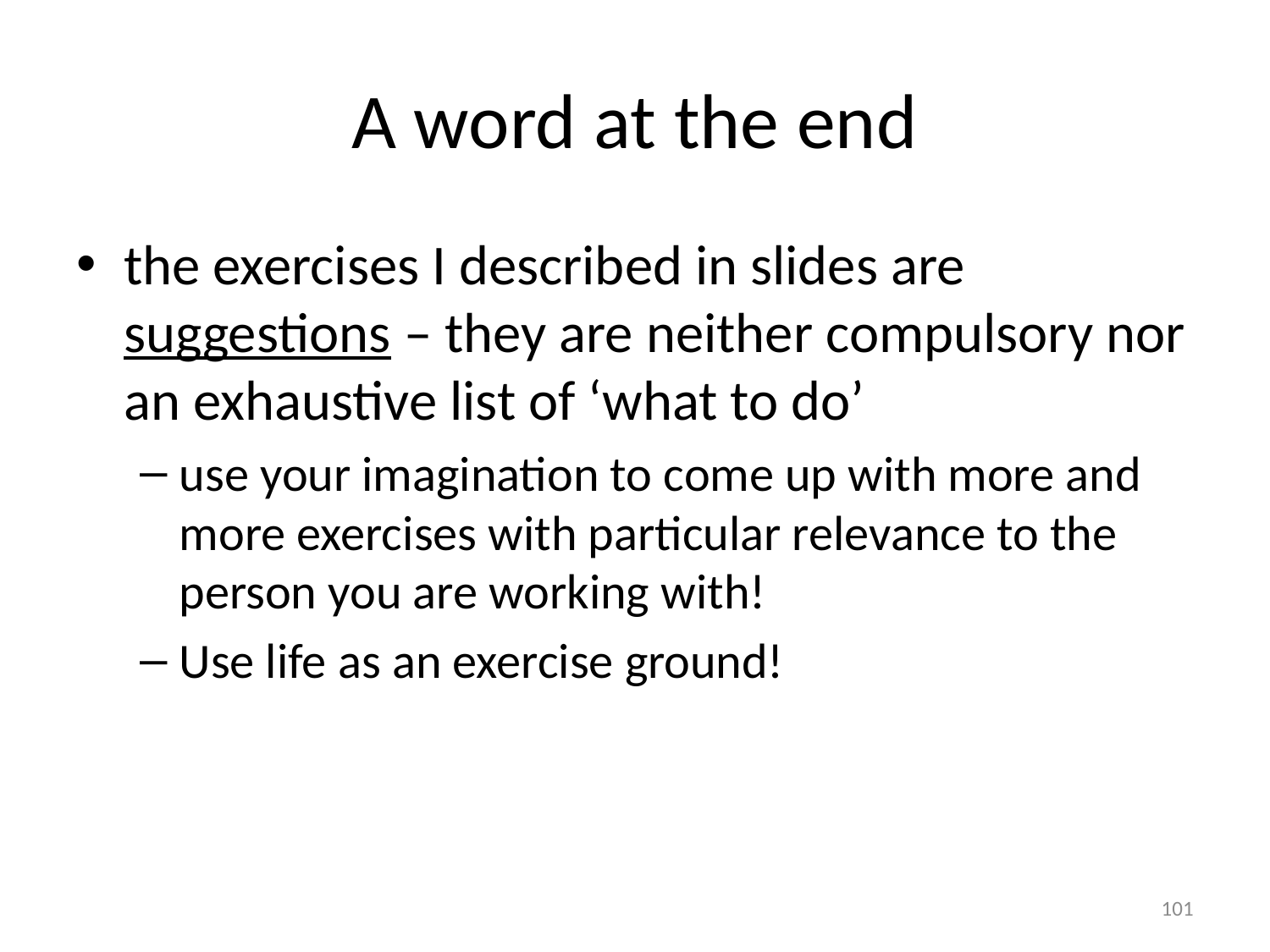

# A word at the end
the exercises I described in slides are suggestions – they are neither compulsory nor an exhaustive list of ‘what to do’
use your imagination to come up with more and more exercises with particular relevance to the person you are working with!
Use life as an exercise ground!
101
